# The Asymmetric Self‐Replicative Alkylation of *N‐*Arylaldimines Using Organolithium Reagents

**DOI:** 10.1002/chem.70998

**Published:** 2026-04-09

**Authors:** Anka Hagelschuer, Paco Visser, Ben L. Feringa

**Affiliations:** ^1^ Stratingh Institute for Chemistry University of Groningen Groningen the Netherlands

**Keywords:** amides, asymmetric amplification, autocatalysis, chirality, lithium

## Abstract

Among the possible explanations for the origin of homochirality on Earth, the propagation of a minute chiral imbalance by asymmetric amplification in chemical reactions, that is, asymmetric autocatalysis, has been widely studied since the 1950s. Despite significant efforts by the chemical community, only one experimental asymmetric autocatalytic system has been reported to date: the enantioselective addition of diisopropyl zinc to pyrimidyl aldehydes, the Soai reaction. Here, we present the rational design of a new transformation that exhibits asymmetric autoinductive behavior: the reaction of organolithium reagents with *N*‐arylaldimines toward the formation of chiral lithium amides. Although our investigations into a variety of reaction systems did not result in chiral amplification, we achieved chiral replication in the alkylation of (*E*)‐1‐(2‐methoxyphenyl)‐*N*‐phenylmethanimine. The addition of *^n^* BuLi this imine in the presence of a 20% enantiomeric excess (*ee)* solution of *N*‐(1‐(2‐methoxyphenyl)pentyl)aniline afforded the desired butylated product in 20% *ee*, for both the (*S)*‐and (*R*)‐enantiomers, representing the first example of organolithium reagents being used in asymmetric autoinductive reactions.

## Graphical abstract

Here, we report a new asymmetric autoinductive reaction; the addition of nBuLi to N‐arylaldimines to form chiral lithium amides. The reaction proceeds with full conversion and complete transfer of chirality, representing the first example of an asymmetric autoinductive reaction with organolithium reagents.

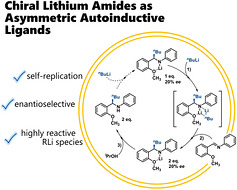

## 1 Introduction

The presence of Nature's essential building blocks as single‐handed molecules—also referred to as “signature of life” [1]—has intrigued scientists since its discovery. Efforts to understand the origin of biological homochirality date back to the early 1950s [2, 3, 4], focusing on two distinct aspects: (1) how did an initial symmetry breaking event, that is, the formation of a small imbalance between the amounts of both enantiomers occur, and (2) how was it propagated via asymmetric amplification toward a homochiral biological world [3, 5]?

One of the proposed mechanisms for the amplification of a chiral bias is the operation of chemical reactions in which a chiral product catalyzes its own formation, commonly referred to as asymmetric autocatalysis [5]. This concept was first proposed as a mathematical model by Frank in 1953, who envisioned a process where a chiral product can catalyze its own formation while simultaneously suppressing the formation of the opposite, undesired enantiomer (mutual antagonism) [6]. Although Frank's seminal work drew significant attention and inspired organic chemists to find experimental evidence for his proposed chiral amplification mechanism, it took more than four decades for the first experimental demonstration with the Soai reaction [7]. In this remarkable discovery the group highlights that the addition of diisopropylzinc to pyrimidine‐5‐carboxaldehyde derivatives proceeds with a significant asymmetric amplification in the presence of its zink‐alkoxide product; over five cycles the *ee* of the (*S*)‐product was amplified from 5% to 90%. The formation of products with a higher *ee* than that of the catalyst, described as a positive nonlinear effect, was discovered by Kagan and coworkers [8] less than ten years before the first report on the Soai reaction, and is now considered a vital aspect of asymmetric autocatalysis [9]. Following their seminal work, the Soai group has described a multitude of examples on asymmetric autocatalysis in the alkylation of pyrimidine‐5‐carboxaldehyde derivatives [10]; notably the amplification of 5 × 10%^−5^% *ee* to > 99.5% *ee* in only three cycles [11], as well as the influence of isotopic exchange [12, 13, 14], chiral additives [15, 16, 17, 18, 19, 20], and circularly polarized light [21]. Despite the tremendous attention that the Soai reaction received by the scientific community [3, 5, 10, 22, 23, 24, 25, 26, 27, 28], resulting in meticulous efforts toward the characterization of the Zn‐alkoxide autocatalyst [16, 29, 30, 31, 32, 33, 34, 35, 36, 37, 38, 39], the rational design of an analogous transformation exhibiting asymmetric autocatalysis remains an elusive target.

**FIGURE 1:**
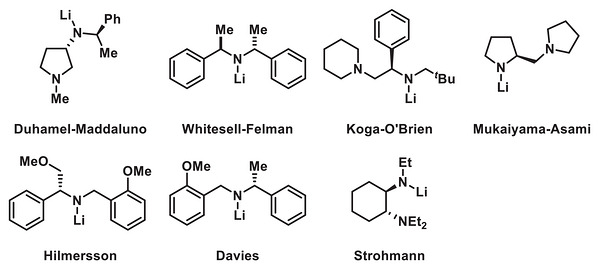
Structures of previously reported classes of CLAs.

In search of a new and distinctive transformation for chiral self‐replication of enantiomerically enriched compounds, we were intrigued by the possibility of exploiting the powerful complexation of chiral lithium amides (CLAs). These scaffolds have been widely used as powerful nucleophiles, bases, and in particular ligands in asymmetric synthesis [40, 41, 42, 43, 44, 45]. Over the past decades, a diverse range of structural examples of CLAs has been developed [44], which are part of the Duhamel–Maddaluno [46, 47, 48, 49], Whitesell–Felman [50, 51], Koga–O'Brien [52, 53], Mukaiyama–Asami [54, 55], Hilmersson [56, 57, 58, 59], Davies [60, 61], and Strohmann [62] classes of CLA which are shown in Figure 1.

The groups of Hilmersson and Harrison–Marchand have previously dedicated particular attention to the use of these scaffolds as ligands for asymmetric synthesis, as well as the spectroscopic analyses of their aggregation states [40]. Based on their studies and the variety of bi‐ and tridentate ligands previously employed in the enantioselective 1,2‐addition reactions to imines [63], we envisioned the rational design of an asymmetric autoinductive imine alkylation reaction using organolithium reagents. This concept is related to the early work of Wynberg and coworkers from our institute on the enantioselective, autoinductive alkylation of benzaldehyde mediated by a chiral metal alkoxide [64]. In contrast to autocatalytic reactions—where the product acts as a catalyst and influences the reaction rate through catalyst multiplication—in an asymmetric autoinductive reaction, the product instead functions as a ligand. As such, it induces stereoselectivity without affecting the overall reaction rate [65].

Here we describe our efforts toward the—to the best of our knowledge—first CLA self‐induced asymmetric alkylation of *N*‐aryl and *N*‐benzylaldimines with organolithium reagents. The transformation displays a profound influence of the product structure, as well as a dependence on the polarity and coordinating ability of the reaction solvent. We achieved self‐induced asymmetric induction by the reaction of *^n^* BuLi with (*E*)‐1‐(2‐methoxyphenyl)‐*N*‐phenylmethanimine in Et_2_O, resulting in chiral replication.

## 2 Project Design

**SCHEME 1:**
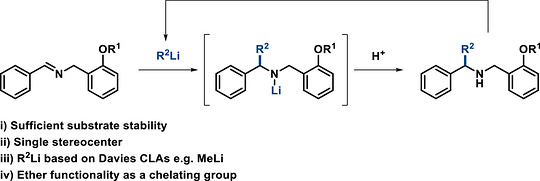
The envisioned asymmetric transformation based on our design constraints: (i) substrate stability, (ii) stereogenic structure, (iii) the R^2^ substituent, and (iv) Li coordinating ability.

As a significant number of CLAs have been previously synthesized and characterized, we considered several structural restraints as a starting point of our investigations: (1) the corresponding imine of the envisioned product should be sufficiently stable, (2) to minimize the number of possible reaction products, the product should contain only one stereocenter, (3) a substituent at the stereogenic center should be installed via addition of a readily generated organolithium reagent to the corresponding imine, and (4) the product should bear a chelating substituent to ensure structural rigidity of the lithium amide [61]. Based on these considerations, and previously reported preliminary investigations by Wynberg and coworkers on the addition of organolithium reagents to *N*‐benzylaldimines [66], we anticipated that a Davies‐type CLA should be a suitable target for our envisioned transformation (Scheme 1). Thereby, we initially studied the alkylation of *N*‐benzylaldimines toward non‐symmetrical, enantioenriched dibenzylamines.

**SCHEME 2:**
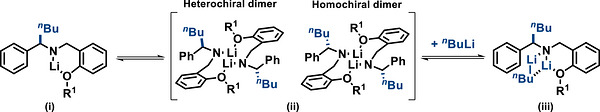
Expected complexation interactions with ^n^BuLi. (i) lithiated monomer, (ii) mutual antagonism due to the difference in stability between homo‐ and heterochiral dimers in the presence of excess ^n^BuLi, and (iii) formation of the CLA/^n^BuLi proposed to be responsible for the autoinductive transfer of the alkyl group.

Based on previous investigations by Harrison–Marchand and coworkers on the structure of Davies‐type CLAs in solution [61] and Hilmersson and Davidsson on a structurally similar Hilmersson‐type CLA [57], we hypothesized that a form of mutual antagonism might arise from the equilibrium between the lithium amide (1) with its dimers (2) in the presence of *^n^* BuLi (Scheme 2). In a non‐enantiopure (scalemic) product mixture, this would give rise to both homo‐ and heterochiral dimers. Based solely on steric interactions, we expected the heterochiral dimer to be more stable than the homochiral dimer, thus enriching the solution with the major, desired enantiomer. This enriched monomeric amide could then coordinate to the organolithium reagent to form the reactive complex (3) (Scheme 2). The equilibrium between (2) and (3) is expected to be both concentration‐ and solvent‐dependent; a shift toward (2) and the tetrameric *^n^* BuLi is observed upon addition of hydrocarbon solvents [57, 59]. However, the formation of the CLA/*^n^* BuLi‐complex (3) is crucial for the success of the asymmetric reaction [67]. A direct correlation between the concentration of the CLA/*^n^* BuLi‐complex (3) and the *ee* of the reaction has previously been reported in the butylation of *ortho*‐tolualdehyde [61]. Furthermore, complex (3) is likely to be more reactive compared to the free organolithium species [44], underscoring its potential role toward enabling the desired asymmetric autoinductive transformation.

Given the strong stoichiometric effects often observed in CLA‐mediated organolithium additions [48, 68], our experimental design primarily employed equimolar amounts of the chiral amine, as ultimately stochiometric amounts would be formed in the alkylation reaction. Nonetheless, to provide a comprehensive understanding, reactions were also carried out using sub‐stochiometric amounts of ligand as well as in the absence of ligand (see ESI Tables S1–S4). The conversion into the desired product—corrected for the initially added amount of catalyst and that of the substrate—were determined by ^1^H NMR spectroscopy versus 1,3,5‐trimethoxybenzene as an internal standard. Before every transformation in the presence of ligand, the *ee* of the initially added amine (*ee*_ini_) was determined by chiral HPLC. After the reaction, the *ee* of the crude reaction mixture (*ee*_obs_) was similar determined. The *ee* of the product, which is formed in the transformation (*ee*_prod_)—corrected for the initially added product—was calculated using Equation 1, which also accounts for the amount of added ligand (*n*_ini_), total product formed (*n*_tot_), and corrected product amount (*n*_prod_) [69].

(1) $$ee_{\mathrm{prod}}=\frac{ee_{\mathrm{obs}}\;*\;n_{\mathrm{tot}}-ee_{\mathrm{ini}}\;*\;n_{\mathrm{ini}}}{n_{\mathrm{prod}}}$$

Equation 1: Equation for the correction of the enantiomeric excess of the crude reaction mixture for the presence of the initially added product.

## 3 Results and Discussion

### 3.1 Self‐Replicative Alkylation of *N*‐Benzylaldimines

We started our investigations with the alkylation of (*E*)‐*N*‐(2‐alkoxybenzyl)‐1‐phenylmethanimine **1** (Table 1). The envisioned amine products were synthesized by reductive amination of (*S*)‐ and rac‐1‐phenylethylamine with the corresponding aldehydes, while the analogous imines were obtained from benzaldehyde and the respective 2‐alkoxybenzylamines (see Scheme S1).

**TABLE 1:** Alkylation of (*E*)‐*N*‐(2‐alkoxybenzyl)‐1‐phenylmethanimine.

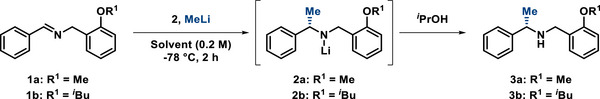			
Entry^a^	Solvent	*R* ^1^	Conv. 1 (3) (%)^b^
1	Toluene	Me	24–34 (< 1)
2	Et_2_O		27–41 (3–7)
3	THF		60–71 (< 1–13)
4	Toluene	*^i^* Bu	16–24 (< 1)
5	Et_2_O		13–24 (< 1–3)
6	THF		53–54 (< 1/7)

^a^ Conditions: To a solution of **3a** or **3b** (1 equiv., 0.2 mmol) at 0°C in the dry solvent was added the MeLi (0.2 equiv. excess relative to the amount of **1** and **3** used in the reaction; 0.6 mL total volume). After 15 min, the reaction mixture was cooled to −78°C, and a solution of **1a** or **1b** (1 equiv., 0.2 mmol) in the dry solvent (0.4 mL) was added dropwise. The reaction was quenched by addition of *^i^* PrOH after 2 h, and the reaction mixture was concentrated in vacuo. All experiments were carried out in duplicate.

^b^ Determined by ^1^H NMR spectroscopy vs. 1,3,5‐trimethoxybenzene as internal standard (^1^H NMR yield of **3** was corrected for the amount of catalyst).

We performed the reactions of (*E*)‐*N*‐(2‐methoxybenzyl)‐1‐phenylmethanimine **1a** and (*E*)‐*N*‐(2‐isobutoxybenzyl)‐1‐phenylmethanimine **1b** with MeLi (0.2 equiv. excess relative to compounds **1** and **3**) in various solvents (Table 1), using stoichiometric amounts of ligands **2a** and **2b**, which were generated by deprotonation of the corresponding enantiomerically pure (*S*)‐amines **3a** and **3b**. Unless stated otherwise, all reaction mixtures obtained in the scope of the paper were colorless to pale yellow homogenous solution prior to quenching. Although moderate to high substrate conversion was observed in toluene, Et_2_O and THF for both **1a** and **1b**, the selectivity into the desired products was fluctuating and non‐reproducible. We also performed the reaction with sub‐stoichiometric amounts (10 mol%) and in the absence of ligand (see ESI Table S1), obtaining similar results.

The addition of **1a** or **1b** to the solution of methyl lithium and the CLA in all cases resulted in the formation of a deeply colored red–purple solution, often associated with the formation of the delocalized 2‐azaallyllithium intermediate [70]. Our observation of the fluctuating substrate conversion, without any significant desired product formation, might thereby be explained by the deprotonation of the starting material to the corresponding azaallyllithium, followed by undesired, non‐productive side reactions [71]. Based on the apparent incompatibility of *N*‐benzylidenebenzylamines with significantly basic organolithium reagents, we next investigated the alkylation of *N*‐phenylaldimines, further restricting the possibility for deprotolithiation‐induced side‐reactivity.

### 3.2 Toward the Self‐Replicative Alkylation of *N*‐Arylaldimines

#### 3.2.1 Alkylation of (*E*)‐*N*‐benzylidene‐2‐Methoxy Imines

**SCHEME 3:**
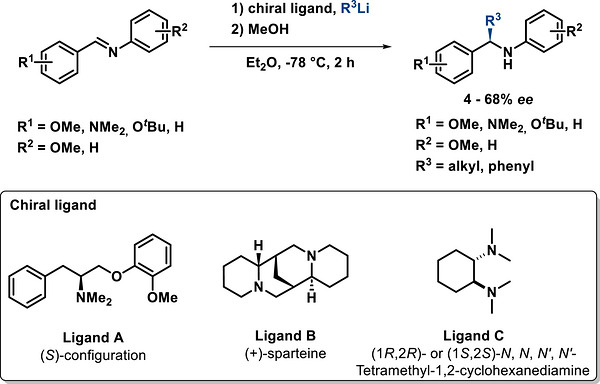
Enantioselective alkylation of *N*‐arylaldimines with R^3^Li in presence of chiral ligands.

To circumvent the undesired side reaction caused by the formation of the 2‐azaallylanion, we considered performing the alkylation of *N*‐arylaldimines, formally removing the benzylic methylene. All enantiomerically enriched *N*‐benzylanilines were synthesized following Scheme 3 (for further information, also for the *ee* of the ligands, see ESI Scheme S3) either using tridentate **ligand A** [72], (+)‐sparteine (**ligand B**) [63], or a chiral diamine (**ligand C**) [73]. The required imines were synthesized by condensation reactions of the corresponding aldehyde and amine (ESI Scheme S2).

The absolute configurations of the enriched chiral amine products were assigned by comparison to the literature following the reaction of (*E*)‐*N*‐benzylidene‐4‐methoxyaniline with organolithium reagents. In this precedent, addition of MeLi in the presence of **Ligand A** afforded the (R)‐configured product [72]. The desired alkylated arylamines were general obtained in satisfactory yields, although with low *ee* (ESI Scheme S3). However, the ability of the products to act as chiral ligands in an autoinductive manner should, in theory, not depend on the *ee*, if a scalemic mixture is used (vide supra). Based on this rational, we proceeded with our studies on the alkylation of *N*‐arylaldimines.

We first investigated the reaction of MeLi with aldimine **4** (Table 2). To our satisfaction, the use of *N*‐arylaldimines as the reaction substrate effectively avoided the previously observed undesired side reaction (Table 1), and a high selectivity for the alkylation product was generally observed. In Et_2_O, the reaction in the absence of **5a** afforded 80% conversion of **4** with excellent selectivity toward **6a** (Table S2, Entry 1), while a slightly decreased conversion of 78% was observed using 10 mol% CLA (Table S2, Entry 2).

**TABLE 2:** Addition of RLi to (*E*)‐*N*‐(2‐methoxyphenyl)‐1‐phenylmethanimine.

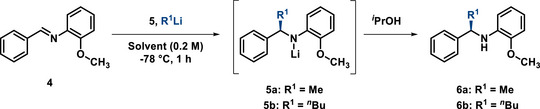						
Entry^a^	Solvent	R^1^Li	Ligand	Conv. 4 (6) (%)^b^	*ee*_ini_ (%)^c^	*ee*_prod_ (%)^c^
1	Et_2_O	Me	**5a**	50 (50)	11	< 1
2	Toluene			64 (36)	12	< 1
3	Et_2_O	*^n^* Bu	**5b**	> 99(> 99)	4	< 1
4^d^	Toluene			n.d.	n.d.	n.d.

^a^ Conditions: To a solution of **6** (0.2 mmol, 1 equiv.) at −78°C in the dry solvent was added the organolithium reagent (0.2 equiv. excess relative to the theoretically total amount of product in the reaction; 0.6 mL total volume). After 30 min, a solution of **4** (0.2 mmol, 1 equiv.) in the dry solvent (0.4 mL) was added dropwise. The reaction was quenched by addition of *^i^* PrOH after 1 h, and the reaction mixture was concentrated in vacuo.

^b^ Determined by ^1^H NMR spectroscopy vs. 1,3,5‐trimethoxybenzene as internal standard (^1^H NMR yield of **6** was corrected for the amount of catalyst).

^c^ Determined by chiral HPLC. *ee*_prod_ determined using Equation 1.

^d^ The lithium amide solution was too viscous to stir.

Surprisingly, only 50% conversion **4** and negligible asymmetric induction was observed in the presence of 1 equiv. **5a** (Table 2, Entry 1), implying that the desired complexation patterns (Scheme 2) are not obtained in the MeLi‐product system. The asymmetric induction in both cases was negligible, further suggesting an unfavorable complexation. Performing the reaction in toluene (Table 2, Entry 2; Table S2, Entries 3 and 4) gave similar results; imperceptible asymmetric induction was observed, and addition of equimolar amounts of ligand significantly diminished the conversion of **4** (Table 2, Entry 2) by more than 10% compared to nonequimolar amounts. In addition, poor selectivity was observed, which might be attributed to the lower solubility of CLAs in nonpolar solvents in comparison to Lewis basic solvents.

We next turned our attention to the reaction of **4** with *^n^* BuLi. High reactivity of the alkylation reaction was observed in both the reaction without ligand and 10 mol% of ligand (see ESI, Table S2, Entries 5–8) as well as in the presence of equimolar amounts of **5b** (Table 2, Entry 3). The transformation in Et_2_O showed some moderate chiral induction (Table 2, Entry 3; Table S2, Entry 6), although importantly, the values of both *ee*_ini_ and *ee*_prod_ can be considered to lie within the error margin of chiral HPLC [74]. The reaction in the presence of 1 equiv. **5b** in toluene did not proceed as the CLA solution became too viscous to stir (Table 2, Entry 4). Based on the obtained results and the poor *ee* observed in the synthesis of **5a** and **5b**, we hypothesized that **4** was strongly complexing the organolithium reagents in a five‐membered fashion, resulting in the intramolecular delivery of the alkyllithium species without facial bias. To improve the chiral induction, we hypothesized that altering the product design to result in a six‐membered chelate instead of a five‐membered Li‐complex, might result in a coordinating effect more comparable to that of similar, previously described CLAs (Figure 1, Koga–Hilmersson and Davies)

#### 3.2.2 Alkylation of *N*‐(2‐Alkoxy)‐ and *N*‐(2‐Dimethylamino)Benzylideneanilines

The desired amines were again synthesized following Scheme 3 (detailed results see ESI, Scheme S3). We were pleased to observe that the asymmetric alkylation of (*E*)‐1‐(2‐methoxyphenyl)‐*N*‐phenylmethanimine with MeLi afforded *(R)‐* **9a** with up to 48% *ee*, while the reaction with *^n^* BuLi gave *(R)*‐**9b** in up to 56% *ee* (ESI, Scheme S3). Depending on the synthetic method used for this reaction, the *ee* for the alkylated *N*‐arylaldimine can differ significantly, as in the case for **9b**, the *ee* can range from 13% to 56%, explaining the distinct initial enantiomeric excesses used in the following reactions. The only ligand that enabled asymmetric formation of **9d** was the chiral diamine (ligand C, Scheme 3), which, in its (*R,R*)‐diastereomeric form, was also used to form (*S*)‐**9b** in 20% *ee*. Unfortunately, we were unable to obtain any of the ligands enantiopure, neither by asymmetric synthesis nor by derivatization and separation of the diastereomers.

Disappointingly, the reaction of **7** with MeLi in Et_2_O did not show a significant influence of the ligand loading. Although the conversion of the substrate increased with equimolar amounts of ligand, the selectivity into **9a** remained relatively constant (Table 3, Entry 1; Table S3, Entries and 2). Negligible chiral induction was observed in presence of catalytic amounts (Table S3, Entry 2), while in the presence of 1 equiv. of ligand, a small amount of the (*S*)‐enantiomer was formed (Table 3, Entry 1). Performing the reaction in toluene yielded very similar results in terms of conversion; however, a slight increase in the chiral induction was observed (Table 3, Entry 2; Table S3, Entries 3 and 4).

**TABLE 3:** Addition of RLi to (*E*)‐1‐(2‐methoxyphenyl)‐*N*‐phenylmethanimine.

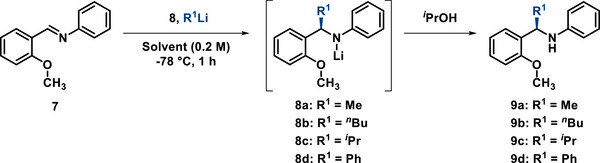						
Entry^a^	Solvent	*R* ^1^Li	Ligand	Conv. 7(9) (%)^b^	*ee*_ini_ (%)^c^	*ee*_prod_ (%)^c^
1	Et_2_O	Me	8a	91 (73)	48	−2^e^
2	Toluene			96 (79)	48	4
3	Et_2_O	*^n^* Bu	8b	99 (94)	56	41
4				91 (91)	16	11
5^d^				> 99(> 99)	13	13
6^d^				> 99(> 99)	−20^e^	−20^e^
7	Toluene			>99(94)	56	16
8	THF			>99(99)	56	−4^e^
9	Et_2_O	*^i^* Pr	8c	>99(>99)	−55^e^	−43^e^
10		Ph	8d	98(97)	28	5

^a^ Conditions: To a solution of **9** (0.2 mmol, 1 equiv.) at −78°C in the dry solvent was added the organolithium reagent (0.2 equiv. excess relative to the theoretically total amount of product in the reaction; 0.6 mL total volume). After 30 min, a solution of **7** (0.2 mmol, 1 equiv.) in the dry solvent (0.4 mL) was added dropwise. The reaction was quenched by addition of *^i^* PrOH after 1 h, and the reaction mixture was concentrated in vacuo.

^b^ Determined by ^1^H NMR spectroscopy vs. 1,3,5‐trimethoxybenzene as internal standard (^1^H NMR yield of **9** was corrected for the amount of catalyst).

^c^ Determined by chiral HPLC, *ee*_prod_ determined using Equation 1.

^d^ use of 2.5 M *^n^* BuLi solution.

^e^ Use and/or formation of the (*S*)‐enantiomer.

Surprisingly, changing the organolithium species from MeLi to *^n^* BuLi resulted in a significant increase in the chiral induction. As observed in previous examples, the amount of ligand did not significantly affect the conversion into **9b**, but unlike the MeLi case, it did have a notable effect on the chiral induction (Table 3, Entries 3–6; Table S3 Entries 5–8). In Et_2_O, a moderate chiral induction was observed in the presence of 10 mol% **8b** (Table S3, Entries 7 and 8), whereas a significant transfer of chirality was achieved in the presence of 1 equiv. of ligand (Table 3, Entries 3–6). Initially, the reactions were performed using a 1.6 M solution of *^n^* BuLi, yielding an *ee*_prod_ of 41% (from 56%, Table 3, Entry 3) and 11% (from 16%, Table 3, Entry 4), respectively. Although a minor loss of chirality was observed in the reaction mixture, these preliminary results do not indicate a non‐linear effect, as the relative chiral induction was identical in both cases, despite the ligands having different *ee* values (detailed calculations see ESI). Given the substantial influence of hydrocarbon solvents on the equilibrium between the tetrameric *^n^* BuLi/dimeric CLA (2) and the active CLA/*^n^* BuLi‐complex (3) (see Scheme 2), we hypothesized that the *ee* could be improved by using a more concentrated 2.5 M solution of *^n^* BuLi. Indeed, by effectively decreasing the hexane concentration in the solvent mixture during the formation of the CLA/*^n^* BuLi complex (7/8 to 3/7), we were pleased to observe that the enantiomeric ratio of the product matched that of the initially added ligand, for both the (*R*)‐ and (*S*)‐enantiomer (Table 3, Entries 5 and 6), representing a genuine autoinduction behavior. In toluene, negligible chiral transfer was observed in the presence of 10 mol% **8b** (Table S3 Entry 9), while increasing ligand loading to 1 equiv. resulted in a moderate chiral induction (Table 3, Entry 7). Again, this might be explained by the non‐coordinating properties of toluene, shifting the equilibrium toward the dimeric CLA and *^n^* BuLi [59]. To our surprise, minimal chiral induction toward the (*S*)‐enantiomer was observed in THF, implying that Et_2_O plays a key role in stabilizing the transition state complex.

With the optimal solvent conditions identified, we aimed to expand the scope of organolithium reagents. The use of *^i^* PrLi resulted in a good chiral induction (Table 3, Entry 9), whereas PhLi afforded only minimal chiral induction (Table 3, Entry 10), highlighting a strong dependence on the nature of the organolithium reagent. The negligible chiral induction with MeLi might be explained by its strong coordination to its own cluster, which can only be deaggregated to a dimeric species in the presence of strong chelating diamines [75]. As a result, the reaction likely proceeds via the non‐coordinated MeLi instead of the CLA/MeLi complex. In contrast, the deaggregation of *^i^* PrLi in the presence of sparteine into a heterodimer similar to compound (3) (Scheme 2) has been described by Strohmann and coworkers [76] suggesting a similar behavior for the CLA/*^n^* BuLi complex used here and likely explaining the high chiral induction. Assuming a similar solvent dependency, the chiral induction achievable with *^i^* PrLi is probably limited by its commercial form; it is only available as a 0.7 M solution in pentane, which increases the ratio of pentane to Et_2_O in the reaction mixture. PhLi, in contrast, forms a strong tetrameric complex with sparteine [77], implying that in the presence of our CLA, the reaction is mainly catalyzed by free PhLi in solution, accounting for the low chiral induction observed.

With the best conditions in hand for the asymmetric autoinduction, (2.5 M solution of *^n^* BuLi, stochiometric amounts of ligand, Table 3, Entry 6), we studied the effect of different chelating groups on the stereochemical outcome of the reaction (Table 4 and Table S4). Based on the previous results, these reactions were only performed with *^n^* BuLi in Et_2_O, as this combination gave the best results in terms of both chiral induction and conversion. By exchanging the methoxy substituent for a dimethylamino functionality, we obtained a structure more reminiscent of the Duhamel–Maddaluno [46, 47, 48, 49], Koga–O'Brien [52, 53], and Mukaiyama–Asami CLAs [78]. Although we were initially concerned that the strongly coordinating nature of **10a** might result in *^n^* BuLi complexation, followed by intramolecular alkylation, diminished substrate conversion was observed in comparison to the alkylation of **7**, both in the absence and presence 10 mol% ligand (Table S4, Entries 1 and 2). Surprisingly, increasing the amount of **11a** resulted in a significantly reduced conversion into **12a** (Table 4, Entry 1). While poor chiral induction was observed in the presence of 10 mol% of ligand (Table S4, Entry 2), formation of the (*S*)‐enantiomer was unexpectedly observed upon increasing the amount of (*R*)‐**11a** to 1 equiv. (Table 4, Entry 1).

**TABLE 4:** Addition of ^*n*^BuLi to (*E*)‐*N*,*N*‐dimethyl‐2‐((phenylimino)methyl)aniline.

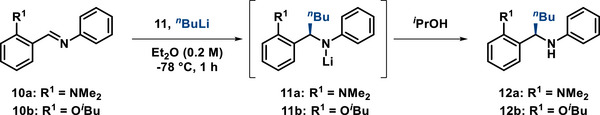					
Entry^a^	*R* ^1^	Ligand	Conv. 10 (12) (%)^b^	*ee*_ini_ (%)^c^	*ee*_prod_ (%)^c^
1	NMe_2_	11a	60 (52)	58	−9^d^
2	O*^i^* Bu	11b	59 (59)	33	−4^d^

^a^ Conditions: To a solution of **12** (0.2 mmol, 1 equiv.) at −78°C in the dry Et_2_O was added *^n^* BuLi (0.2 equiv. excess relative to the theoretically total amount of product in the reaction; 0.6 mL total volume). After 30 min, a solution of **10** (0.2 mmol) in the dry Et_2_O (0.4 mL) was added dropwise. The reaction was quenched by addition of *^i^* PrOH after 1 h, and the reaction mixture was concentrated in vacuo.

^b^ Determined by ^1^H NMR spectroscopy vs. 1,3,5‐trimethoxybenzene as internal standard (^1^H NMR yield of **12** was corrected for the amount of catalyst).

^c^ Determined using chiral HPLC, *ee*_prod_ determined using Equation 1.

^d^ Formation of the (*S*)‐enantiomer.

Having established that a stronger substrate complexation negatively affects the enantioselectivity of the transformation, we hypothesized that the use of a more sterically encumbered product could form a catalytic aggregate, which exhibits a higher facial selectivity for the alkylation reaction. We hence investigated the transformation of isobutoxy‐substituted aldimine **10b**. The positioning of an isobutoxy functionality in close proximity to the C = N bond resulted in diminished conversion of **10b** in all reactions (Table 4, Entry 2; Table S4, Entries 3 and 4). Furthermore, the increased steric bulk of the product (i.e., the anticipated chiral ligand) seemed to disrupt the complexation of **12b**, largely eliminating any the chiral induction in this transformation (Table 4, Entry 2). Thus, the only conditions that show an asymmetric autoinductive behavior are ligand **9b** in presence of 2.5 M solution of *^n^* BuLi.

### 3.3 Further Efforts to Improve the Stereoselective Outcome of the Reaction

A temperature dependence for the ligand‐mediated alkylation of imines [73, 79] and aldehydes [67] with RLi has been previously reported. In general, the enantioselectivity increases with decreasing temperatures while the reactivity of *^n^* Buli increases with elevated temperatures [80].

**SCHEME 4:**
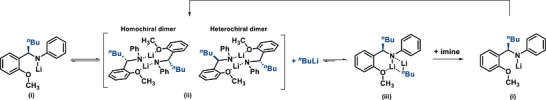
Envisioned reaction mechanism for the CLA‐mediated addition of ^*n*^BuLi to (*E*)‐1‐(2‐methoxyphenyl)‐*N*‐phenylmethanimine at lower temperatures.

As the reaction is an equilibrium between the unreactive dimers (2) plus uncomplexed *^n^* BuLi and complex (3) (Scheme 4), we hypothesized that by lowering the temperature, the equilibrium could be shifted more toward the reactive CLA/*^n^* BuLi complex (3), affording the desired lithiated product in an enantioselective fashion. The resulting product could then re‐enter the equilibrium and form complex (3) with the remaining unreacted *^n^* BuLi, leading to an enrichment of the desired enantiomer.

We were pleased to observe that by decreasing the reaction temperature to −95°C, chiral amplification could be obtained (Table 5, Entries 1 and 2). Unfortunately, the stereoselectivity and conversion into the desired product was fluctuating, and the formation of a red solution was occasionally observed. To our surprise however, we observed the same chiral induction at −50°C (Table 5, Entry 4) as we observed under the standard reaction conditions, highlighting that the ligand‐mediated asymmetric addition pathway is still prevalent at this temperatures and that the transformation is robust with regard to the reaction temperature.

**TABLE 5:** Addition of ^*n*^BuLi to (*E*)‐1‐(2‐methoxyphenyl)‐*N*‐phenylmethanimine at different temperatures.

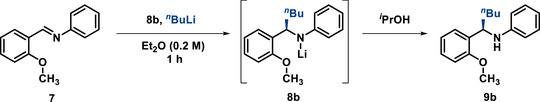				
Entry^a^	Temperature (°C)	Conv. 7 (9b) (%)^b^	*ee*_ini_ (%)^c^	*ee*_prod_ (%)^c^
1	−95	> 99(> 99)	12	16
2		> 99(> 99)	10	15
3		75(75)	13	13
4	−50	> 99(> 99)	13	13

^a^ Conditions: To a solution of **9b** (0.2 mmol, 1 equiv.) at the given temperature in anhydrous Et_2_O was added *^n^* BuLi (0.2 equiv. excess relative to the theoretically total amount of product in the reaction; 0.6 mL total volume). After 30 min, a solution of **7** (0.2 mmol, 1 equiv.) in anhydrous Et_2_O (0.4 mL) was added dropwise. The reaction was quenched by addition of *^i^* PrOH after 1 h, and the reaction mixture was concentrated in vacuo.

^b^ Determined by ^1^H NMR spectroscopy vs. 1,3,5‐trimethoxybenzene as external standard (^1^H NMR yield of **9b** was corrected for the amount of catalyst).

^c^ determined by chiral HPLC, *ee*_prod_ determined using Equation 1.

Given the positive effect of lithium salts on the enantioselectivity in CLA‐assisted deprotonation of aldehydes [81, 82], we set out to investigate the effect of different Li salts on the stereoselective outcome of the reaction. Interestingly, a significant decrease in *ee* and reactivity was observed. (see Table S6, ESI)

### 3.4 Iterative Addition

Having established a system which exhibits autoinductive chiral replication, we investigated the possibility of performing an iterative addition sequence aiming to produce increased amounts of ligand effectively from a small amount (Table 6). Instead of quenching the reaction after 1 h, additional *^n^* BuLi was added to the reaction mixture to regenerate the CLA/*^n^* BuLi complex, which could then react with freshly added imine (pathway a). Ideally, each iterative addition would double the amount of ligand obtained, without compromising the enantiomeric excess.

**TABLE 6:** Iterative addition of ^*n*^BuLi to (*E*)‐1‐(2‐methoxyphenyl)‐*N*‐phenylmethanimine.

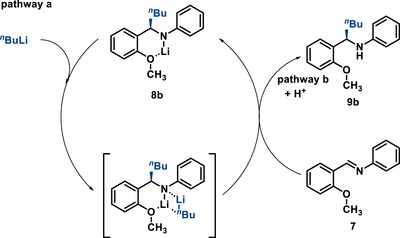				
Entry^a^	Conv. 7 (%)	*ee*_ini_ (%)^b^	*ee_obs_* (%)^b^	*Ø*_chiral,obs_ ^c^
1a	> 99	14	14	1
1b	> 99	14	10	0.8
1c	> 99	10	8	0.8
2^d^	> 99	11	8	0.7
3a	> 99	10	10	1
3b	> 99	10	9	0.9
4a	> 99	−16^e^	−16^e^	1
4b	> 99	−16^e^	−14^e^	0.9
4c^f^	95	−14^e^	−10^e^	0.7

^a^ Conditions: To a solution of **9b** (0.2 mmol1 equiv.) at −78°C in anhydrous Et_2_O was added *^n^* BuLi (0.2 equiv. excess relative to the theoretically total amount of product in the reaction; 0.6 mL total volume). After 30 min, a solution of **7** (0.2 mmol, 1 equiv.) in anhydrous Et_2_O (0.4 mL) was added dropwise. The reaction was stirred for 1 h, and fresh *^n^* BuLi (0.2 equiv. excess relative to the theoretically total amount of product in the reaction) was added. After 30 min, a solution of **7** (1 equiv. relative to the theoretically total amount of product) in anhydrous Et_2_O was added, and the reaction was stirred for 1 h. This procedure was performed 2 times in total. Afterward, the reaction was quenched by the addition of *^i^* PrOH.

^b^ Determined by chiral HPLC, *ee*_prod_ determined using Equation 1.

^c^ Determined using Equation S1, *ee*_obs_ was used instead of *ee*_prod._

^d^ No aliquots were taken in between.

^e^ Opposite enantiomer.

^f^ Reaction turned red.

Instead of focusing on the enantiomeric excess of the product (*ee*_prod_), we now monitored the enantiomeric excess of the reaction solution (*ee*_obs_) as well as its chiral induction factor (*Ø*_chiral, obs_) (calculation see ESI), as this directly reflects the *ee* of the ligand present during each iterative addition cycle. Initially, we attempted to continue the reaction by adding new *^n^* BuLi to the solution containing intermediate **8b** (Table 6). Under these conditions, however, a decrease in enantiomeric excess was observed after both the first (Table 6, Entry 1b) and second (Table 6, Entry 1c) iterative addition. Starting from an initial value of 14% ee, the *ee*_obs_ dropped to 10% after the first addition and to 8% after the second, corresponding to a *Ø*_chiral, obs_ of 0.8 in both cases. A similar result was obtained when the reaction was performed without taking aliquots between additions and quenched after two iterations (Table 6, Entry 2).

Given the strong dependence of the stereochemical outcome on the solvent composition, we explored whether diluting the reaction mixture containing intermediate **8b** with Et_2_O prior to adding *^n^* BuLi could improve the overall *ee*. Upon dilution of the reaction mixture with Et_2_O prior to initiating a new iterative addition cycle (Table 6, Entries 3a and b, 4a–c), better chiral induction was observed as compared to Entry 1. As expected, the initial addition of the chiral ligand to the imine led to chiral replication, followed by a modest decrease in *ee*_obs_ from 10% to 9% or 16% to 14%, respectively (*Ø*_chiral, obs_ = 0.9), after the first iteration. During the second addition, however, the *ee*_obs_ dropped more significantly (from 14% to 10%, Entry 4c, Table 6). Notably, a color change from pale yellow to red occurred during this cycle. The reaction color is generally a reliable indicator of the reaction's progress: pale yellow corresponds to full conversion and effective chiral replication, whereas a transition to red is associated with reduced conversion and diminished *ee*. Formation of a red solution is not only seen during the iterative additions but also during quenching when *^i^* PrOH is not added under inert conditions. Under such conditions, the solution turns red, suggesting an undefined, oxidative pathway. In the context of the iterative addition, this implies that maintaining strict inert conditions is challenging, yet needed, especially because new solvent and *^n^* BuLi must be introduced repeatedly. Partial quenching or degradation of the CLA/*^n^* BuLi complex into amine **9** (pathway b) would reduce its capacity to add to the imine in an enantioselective manner, leading to the observed decrease in *ee*. Apart from the high sensitivity of the system and its accompanying minor loss in enantioselectivity, the iterative addition is an elegant method for rapidly multiplying the amount of ligand. Starting with 1 equiv. of ligand, a theoretical amount of 4 equiv. of ligand can be obtained within one iterative addition cycle without overly compromising enantioselectivity.

## 4 Conclusion

In summary, we have developed a new transformation exhibiting asymmetric autoinduction in the alkylation of *N*‐arylaldimines with organolithium reagents. While *N*‐benzylaldimines proved unsuitable as ligands in the transformation, likely due to the formation of azaalylanions in the presence of highly basic organolithium reagents, the use of *N*‐arylaldimines showed significant potential. Remarkably, expanding the intramolecular coordination from a five‐ to a six‐membered ring dramatically enhanced enantioselectivity, as observed in the reaction of (*E*)‐1‐(2‐isomethoxyphenyl)‐*N*‐phenylmethanimine with a 2.5 M solution of *^n^* BuLi. We hypothesize that the success of the reaction depends on the presence of the CLA/RLi complex in solution, whose formation is favored in absence of non‐coordinating solvents and RLi reagents that readily deaggregate. Furthermore, we found that increasing the coordinating ability of the product, or its steric bulk was detrimental to the chiral induction. The product's ability to function as a chiral ligand in an iterative protocol shows high sensitivity, a common phenomenon in the context of organolithium reagents. Still, it can be used in an iterative addition, effectively increasing the amount of ligand by 4 (or more) with just a minor drop in enantioselectivity. Overall, and to the best of our knowledge, this represents the first example of an autoinductive transformation with chiral replication specifically employing CLAs.

## Conflicts of Interest

The authors declare no conflicts of interest.

## Supporting information




**Supporting File 1**: The authors have cited additional references within the Supporting Information [72, 83, 84, 85, 86, 87, 88, 89, 90, 91, 92, 93, 94].
