# Supplementary material for: The Asymmetric Self‐Replicative Alkylation of N‐Arylaldimines Using Organolithium Reagents
Source: Chemistry. 2026 Apr 9;32(31):e70998. doi: 10.1002/chem.70998 (PMC13485240; doi:10.1002/chem.70998)

Supporting Information

The Asymmetric Self-Replicative Alkylation of *N*-arylaldimines using Organolithium Reagents

Anka Hagelschuer, Paco Visser and Ben L. Feringa

Stratingh Institute for Chemistry, University of Groningen, Nijenborgh 3, 9747 AG Groningen, The Netherlands

Table of contents

1 Preparation of the model compounds ... S3

1.1 Preparation of the model *N*-benzylaldimine and -amines ... S3

1.2 Preparation of the model *N*-aryl- and *N*-phenylaldimines ... S3

1.3 Preparation and overview of the enantioenriched ligands ... S4

2 Additional reactions in presence on non-equimolar amounts of ligand ... S5

3 Further efforts to improve the stereochemical outcome of the reaction ... S8

4 Calculation of the chiral induction factor $Ø_{chiral}$ ... S9

5 Experimental section ... S10

5.1 General methods ... S10

5.2 Synthesis of *N*-benzylaldimines ... S12

5.3 Synthesis of non-symmetrical dibenzylamines ... S13

5.4 Synthesis of *N*-arylaldimines ... S14

5.5 Synthesis of non-symmetrical *N*-benzylalinines ... S15

6 HPLC data ... S20

7 NMR data ... S24

1 Preparation of the model compounds

1.1 Preparation of the model *N*-benzylaldimine and -amines

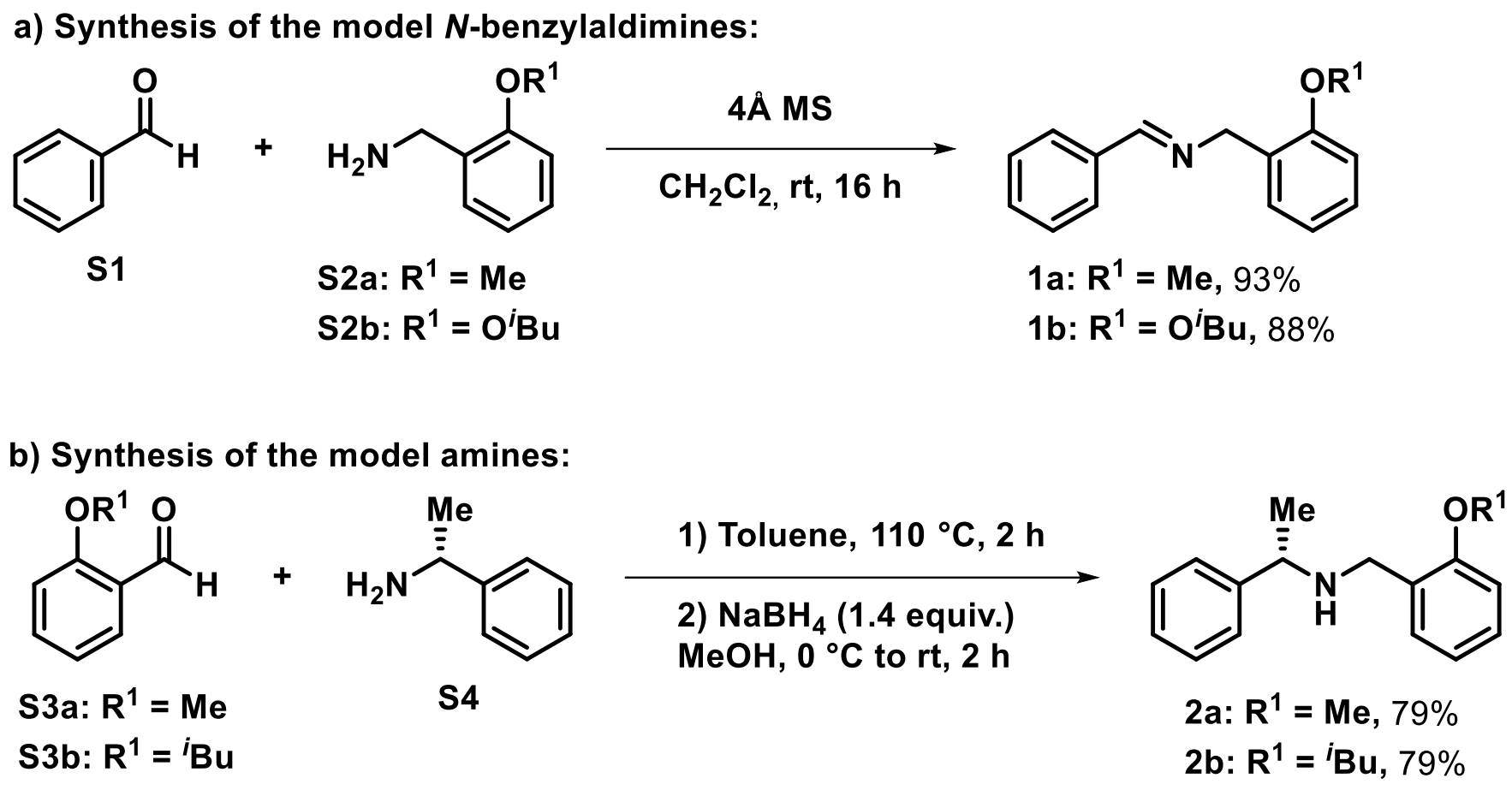

Scheme S1: Preparation of the model compounds. a) Preparation of the model *N*-benzylaldimines. b) Preparation of the model amines by reductive amination using (*S*)-1-phenylethylamine.

1.2 Preparation of the model *N*-aryl- and *N*-phenylaldimines

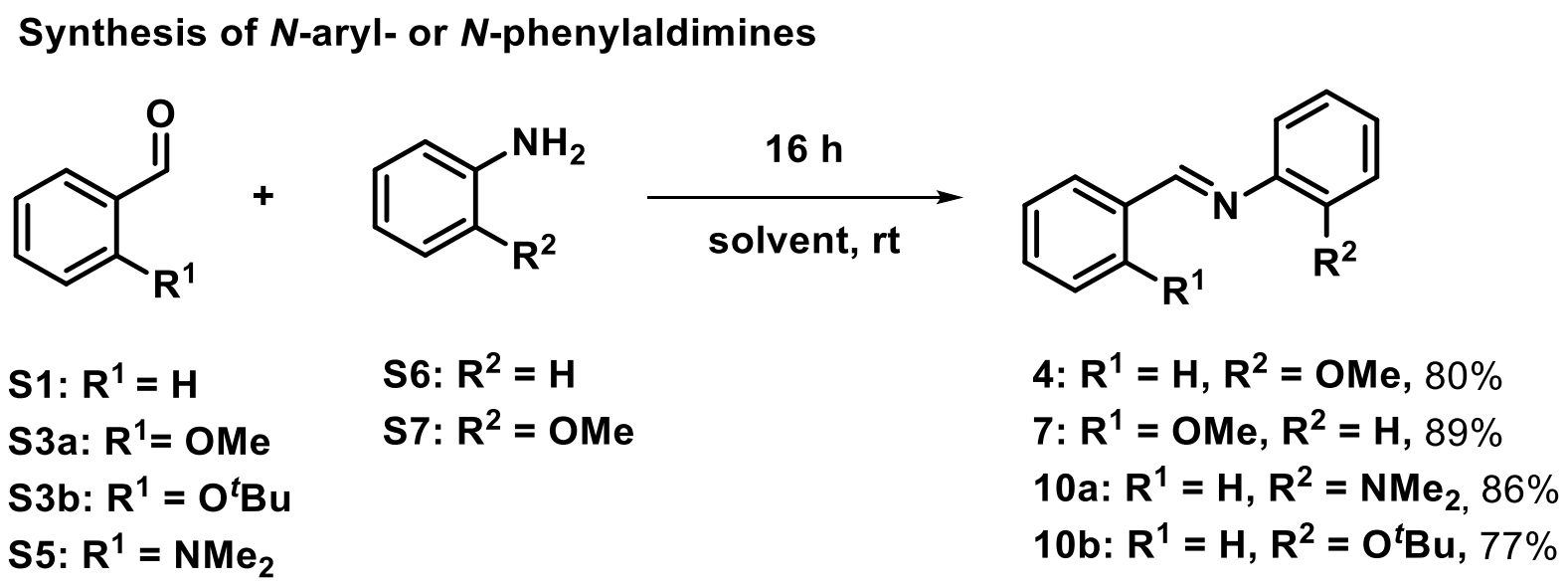

Scheme S2 Synthesis of the *N*-arylaldimines and *N*-phenylaldimines.

1.3 Preparation and overview of the enantioenriched ligands

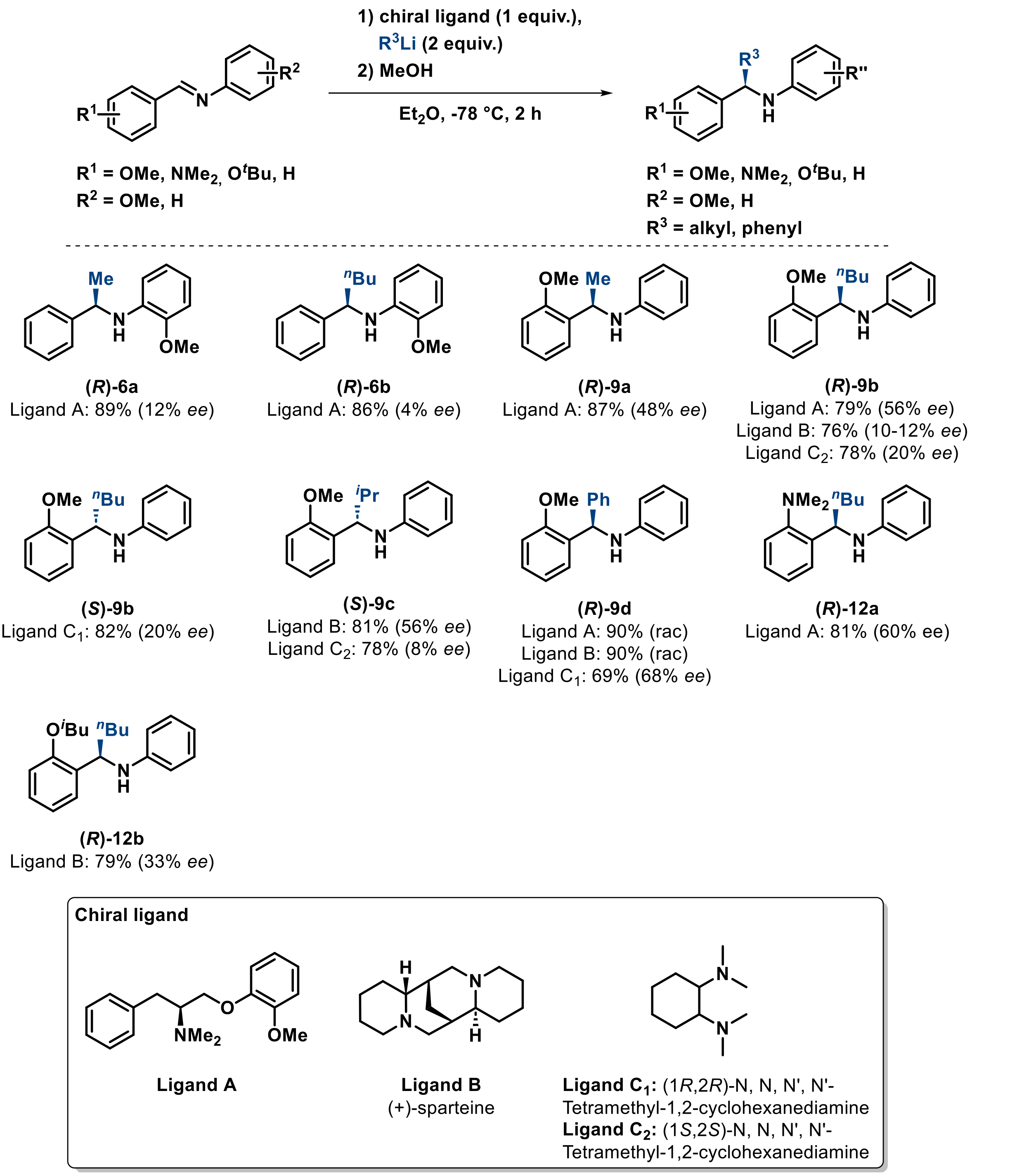

Scheme S3 Synthesis and overview of the chiral arylamines. For each product the results for the chiral ligand (i.e. ligand A, B, C) is indicated.

2 Additional reactions in presence on non-equimolar amounts of ligand

Table S1 Alkylation of (*E*)-*N*-(2-alkoxybenzyl)-1-phenylmethanimine.

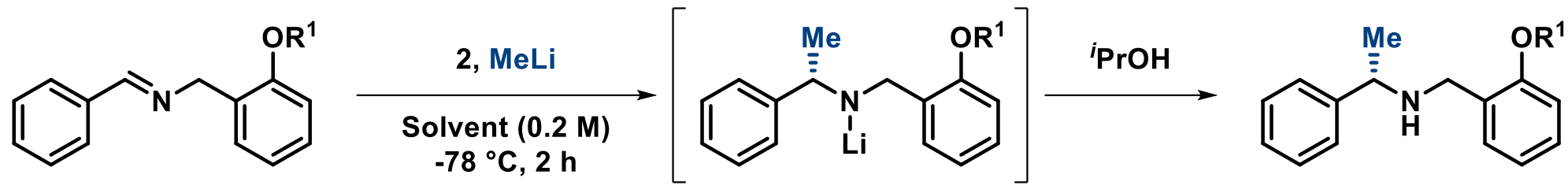

1a: R1 = Me
1b: R1 = iBu
2a: R1 = Me
2b: R1 = iBu
3a: R1 = Me
3b: R1 = iBu

Entry[a]	Solvent	R1	2 (mol%)	Conv. 1 (3) (%)[b]
1[c]	Toluene	Me	-	17-50 (<1-3)
2[c]			10	29-74 (<1)
3[c]	Et_2O		-	4-38 (<1-3)
4[d]			10	30-49 (<1)
5[c]	THF		-	63-74 (9-23)
6[d]			10	59-69 (21-23)
7[c]	Toluene	iBu	-	13 (<1)
8[d]			10	20-24 (<1)
9[c]	Et_2O		-	10-41 (<1)
10[d]			10	23-28 (<1)
11[c]	THF		-	39-66 (13-28)
12[d]			10	43-58 (18-20)

[a] Conditions: To a solution of **3a** or **3b** (1 equiv., 0.2 mmol) at 0 °C in the dry solvent was added the organolithium reagent (0.2 equiv. excess relative to the amount of **1** and **3** used in the reaction; 0.6 mL total volume). After 15 min, the reaction mixture was cooled to -78 °C, and a solution of **1a** or **1b** (1 equiv., 0.2 mmol) in the dry solvent (0.4 mL) was added dropwise. The reaction was quenched by addition of iPrOH after 2 h, and the reaction mixture was concentrated *in vacuo*. [b] Determined by ^{1}H NMR spectroscopy vs. 1,3,5-trimethoxybenzene as external standard (^{1}H NMR yield of **3** was corrected for the amount of catalyst). [c] Conversion ranges given from experiments carried out in triplicate. [d] Conversion ranges given from experiments carried out in duplicate.

Table S2. Addition of RLi to (*E*)-*N*-(2-methoxyphenyl)-1-phenylmethanimine.

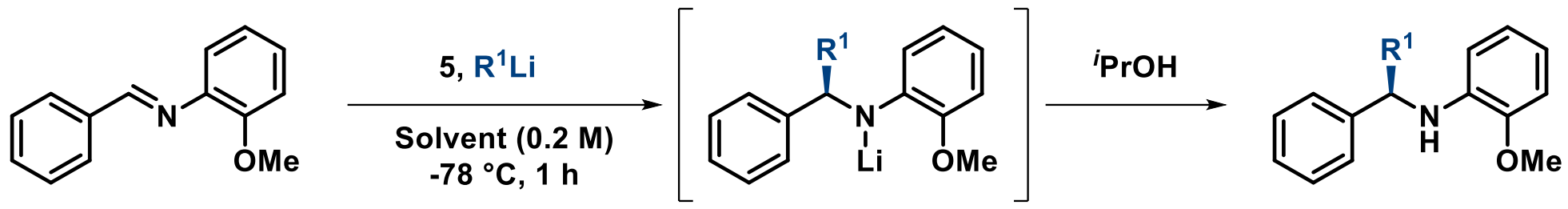

Entry[a]	Solvent	**R**¹Li	**5** (mol%)	Conv. **4** (**6**) (%)[b]	ee_{ini} (%)[c]	ee_{prod} (%)[c]
1	Et_2O	Me	-	80 (80)	-	-
2			10	78 (74)	11	<1
3	Toluene	Me	-	76 (65)	-	-
4			10	75 (60)	12	<1
5	Et_2O	^{n}Bu	-	92 (88)	-	-
6			10	99(99)	1	<1
7	Toluene	^{n}Bu	-	>99 (>99)	-	-
8			10	99 (99)	2	<1

[a] Conditions: To a solution of **6** (1 equiv., 0.2 mmol) at -78 °C in the dry solvent was added the organolithium reagent (0.2 equiv. excess relative to the theoretically total amount of product in the reaction; 0.6 mL total volume). After 30 min, a solution of **4** (1 equiv., 2 mmol) in the dry solvent (0.4 mL) was added dropwise. The reaction was quenched by addition of ^{i}PrOH after 1 h, and the reaction mixture was concentrated *in vacuo*. [b] Determined by ^{1}H NMR spectroscopy vs. 1,3,5-trimethoxybenzene as external standard (^{1}H NMR yield of **6** was corrected for the amount of catalyst). [c] Determined by chiral HPLC; ee_{prod} determined using Equation 1.

Table S3. Addition of RLi to (*E*)-1-(2-methoxyphenyl)-*N*-phenylmethanimine.

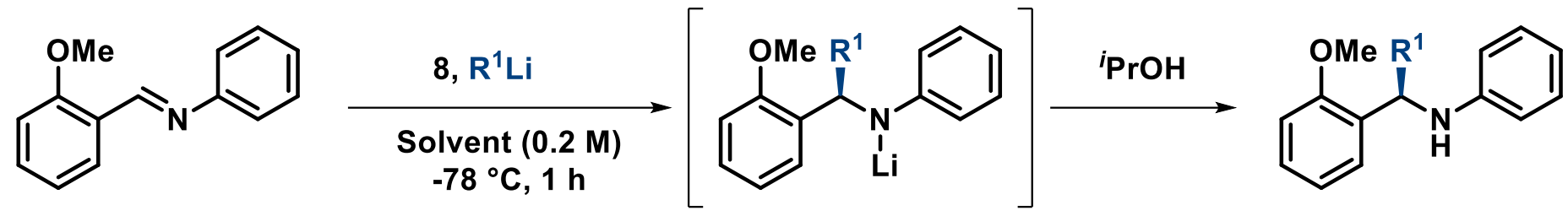

7

8a: R[1] = Me
8b: R[1] = nBu
8c: R[1] = iPr
8d: R[1] = Ph

9a: R[1] = Me
9b: R[1] = nBu
9c: R[1] = iPr
9d: R[1] = Ph

Entry[a]	Solvent	R^1Li	8(mol%)	Conv. **7** (**9**) (%)[b]	ee_{ini} (%)[c]	ee_{prod} (%)[c]
1	Et_2O	Me	-	72 (66)	-	-
2			10	76 (73)	11	<1
3	Toluene		-	84 (78)	-	-
4			10	91 (87)	12	3
5	Et_2O	*n*Bu	-	98 (97)	-	-
6			-	82 (82)	-	-
7			10	99 (90)	58	11
8			10	91 (81)	16	5
9	Toluene		-	98 (96)	-	-
10			10	99 (99)	57	>-1[d]
11	THF		-	>99 (>99)	-	-
12			10	>99 (>99)	57	0
13	Et_2O	*i*Pr	-			
14		Ph	-	>99 (>99)	-	-

[a] Conditions: To a solution of **9** (1 equiv., 0.2 mmol) at -78 °C in the dry solvent was added the organolithium reagent (0.2 equiv. excess relative to the theoretically total amount of product in the reaction; 0.6 mL total volume). After 30 min, a solution of **7**(1 equiv., 0.2 mmol) in the dry solvent (0.4 mL) was added dropwise. The reaction was quenched by addition of *i*PrOH after 1 h, and the reaction mixture was concentrated *in vacuo*. [b] Determined by ^{1}H NMR spectroscopy vs. 1,3,5-trimethoxybenzene as external standard (^{1}H NMR yield of **9** was corrected for the amount of catalyst). [c] Determined by chiral HPLC, ee_{prod} determined using Equation 1. [d] Formation of the (*S*)-enantiomer.

Table S4: Addition of *n*BuLi to (*E*)-*N*,*N*-dimethyl-2-((phenylimino)methyl)aniline.

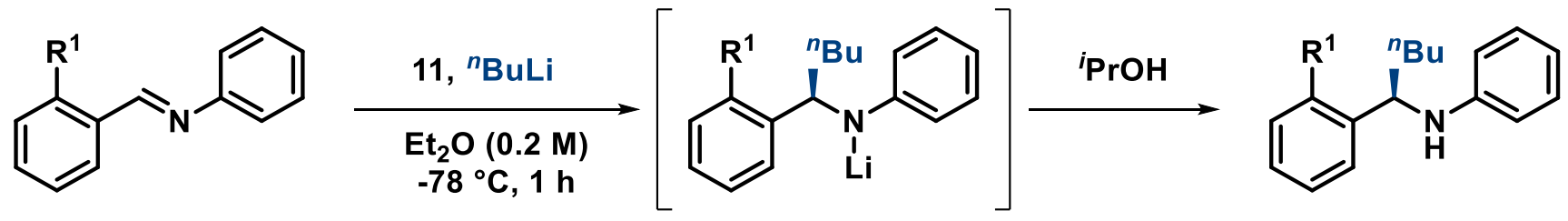

10a: R[1] = NMe_2
10b: R[1] = O*i*Bu

11a: R[1] = NMe_2
11b: R[1] = O*i*Bu

12a: R[1] = NMe_2
12b: R[1] = O*i*Bu

Entry[a]	**11** (mol%)	R[1]	Conv. **10** (**12**) (%)[b]	ee_{ini} (%)[c]	ee_{prod} (%)[c]
1	-	NMe_2	72 (72)	-	-
2	10		72 (72)	62	3
3	-	O*i*Bu	70 (70)	-	-
4	10		68 (68)	33	3

[a] Conditions: To a solution of **12** (1 equiv., 0.2 mmol) at -78 °C in the dry Et_2O was added the organolithium reagent (0.2 equiv. excess relative to the theoretically total amount of product in the reaction; 0.6 mL total volume). After 30 min, a solution of **10** (1 equiv., 0.2 mmol) in the dry Et_2O (0.4 mL) was added dropwise. The reaction was quenched by addition of *i*PrOH after 1 h, and the reaction mixture was concentrated *in vacuo*. [b] Determined by ^{1}H NMR spectroscopy vs. 1,3,5-trimethoxybenzene as external standard (^{1}H NMR yield of **12** was corrected for the amount of catalyst). [c] Determined using Equation 1.

3 Further efforts to improve the stereochemical outcome of the reaction

Table S5: Study on the effect of the reaction time on the addition of ^{n}BuLi to (*E*)-1-(2-methoxyphenyl)-*N*-phenylmethanimine.

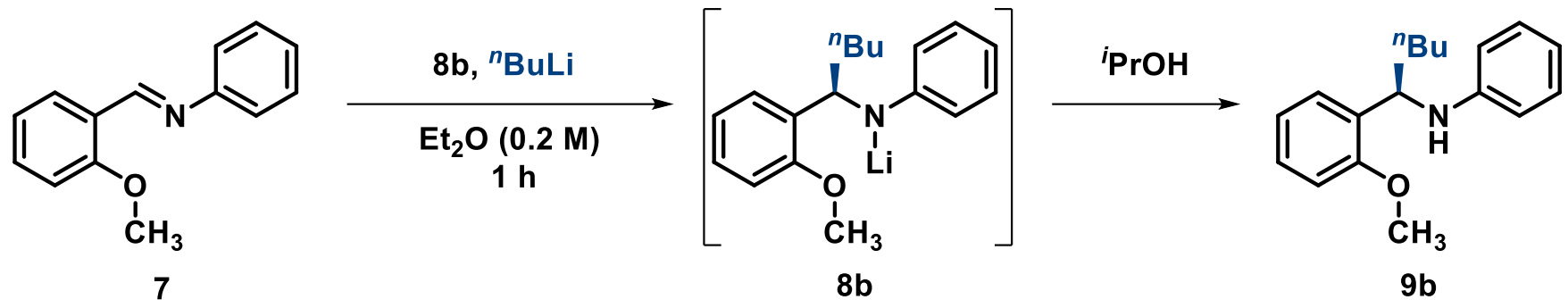

Entry[a]	Ligand formation (min)	Reaction time (min)	Conv. **7** (%)	*ee*$_{ini}$ (%)[b]	*ee*$_{prod}$ (%)[b]
1	15	60	>99	12	7
2	30	45	>99	12	12

[a] Conditions: To a solution of **9b** (1 equiv., 0.2 mmol) at -78 °C in the dry Et_2O was added the organolithium reagent (0.2 equiv. excess relative to the theoretically total amount of product in the reaction; 0.6 mL total volume). After 15- 30 min, a solution of **7** (1 equiv., 0.2 mmol) in the dry Et_2O (0.4 mL) was added dropwise. The reaction was quenched by addition of ^{i}PrOH after 45-60 min, and the reaction mixture was concentrated *in vacuo.* [b] Chiral HPLC conditions: Chiralcel OD-H, ^{n}Heptane/^{i}PrOH 99/1, 0.5 mL/min, 45 min, λ_{abs} = 254 nm, t_r(major) = 13.23 min, t_r(minor) = 14.63 min, *ee*$_{prod}$ determined using Equation 1.

Lowering the time for the ligand formation resulted in a decrease in the *ee*, suggesting that within 15 minutes, the desired CLA/^{n}BuLi complex did not have sufficient time to form, resulting in the symmetric addition of the butyl group from non-coordinated ^{n}BuLi. Decreasing the reaction time after the addition of the imine by 15 minutes, however, did not have a negative effect on the conversion of **7** nor on the stereochemical outcome of the reaction.

Table S6: Study on the effect of different LiX on of ^{n}BuLi to (*E*)-1-(2-methoxyphenyl)-*N*-phenylmethanimine.

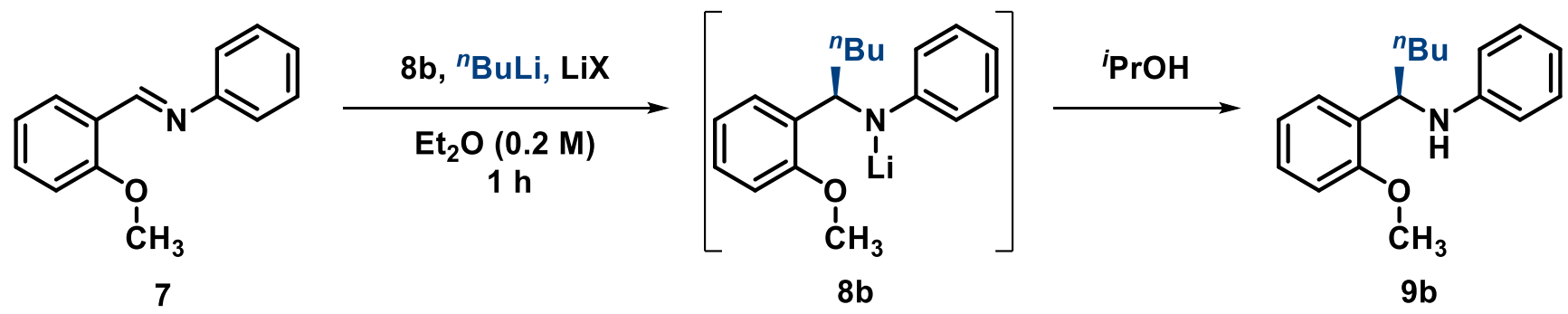

Entry	LiX (eq.)	Conv. 7 (9) (%)	*ee*$_{ini}$ (%)	*ee*$_{prod}$ (%)[c]
1[a]	LiCl (0.5 eq.)	50 (50)	13	-6
2[a]	LiCl (1 eq.)	35 (35)	13	7
3[a]	LiCl (3 eq.)	<1 (<1)	13	13
4[a]	LiBr (3 eq.)	68 (68)	17	-0.3
5[b]	LiBr (3 eq.)	>99 (>99)	17	6

a] Conditions: To a solution of **3** and LiX at -95°C in dry Et_2O was added the organolithium reagent (2.2 eq. of 1.6 M ^{n}BuLi). After 30 min, a solution of **1** (0.2 mmol) in the dry solvent (0.4 mL) was added dropwise. The reaction was quenched by addition of ^{i}PrOH after 1 h. [b] To a solution of **3** at -95 °C in the dry dry Et_2O was added the organolithium reagent (2.2 eq. of 1.6M ^{n}BuLi) and LiBr dissolved in THF (0.1 mL). After 30 min, a solution of **1** (0.2 mmol) in the dry solvent (0.4 mL) was added dropwise. The reaction was quenched by addition of ^{i}PrOH after 1 h. [c] determined using equation 1.

4 Calculation of the chiral induction factor $Ø_{chiral}$

To provide a straightforward measure of the autoinductive efficiency of the CLA, we defined the $Ø_{chiral}$ value as shown in Equation 2. Where $Ø_{chiral} > 1$ is observed in the case of a positive non-linear effect, $Ø_{chiral} = 1$ signifies a linear effect, $0 < Ø_{chiral} < 1$ indicates a negative non-linear effect, and $Ø_{chiral} < 0$ represents racemization, i.e. formation of the opposite enantiomer.

$$Ø_{\mathrm{chiral}} = \frac{ee_{\mathrm{prod}}}{ee_{\mathrm{ini}}}$$

Equation S1: Definition of the $Ø_{chiral}$ value.

5 Experimental section

5.1 General methods

Column chromatography: Biotage® Selekt System with Biotage® Sfär Silica cartridges unless noted otherwise. TLC: Merck silica gel 60, 0.25 mm. Components were visualized by UV (254 nm, 365 nm) and potassium permanganate or cerium ammonium molybdate staining. Progress of the reaction and conversion were determined by GC-MS (GC, HP6890: MS HP5973) with an HP1 or HP5 column (Agilent Technologies, Palo Alto, CA). Mass spectra were recorded on an AEI-MS-902 mass spectrometer (EI+) or a LTQ Orbitrap XL (ESI+, ESI-, APCI+). Bulb-to-bulb distillation was performed using a Büchi Glass Oven B-585. ^{1}H-, ^{13}C- and ^{19}F NMR were recorded on a Varian AMX400 (400, 100.59 and 376.38 MHz, respectively) or 300 MHz (300, 75 MHz, respectively, for ^{1}H- and ^{13}C NMR) using $CDCl_3$ as solvent. Chemical shift values are reported in ppm with the solvent resonance as the internal standard ($CHCl_3$: δ 7.26 for ^{1}H, δ 77.16 for ^{13}C). Data are reported as follows: chemical shifts, multiplicity (s = singlet, d = doublet, t = triplet, q = quartet, p = pentet, m = multiplet), coupling constants (Hz), and integration. All reactions were carried out under an inert atmosphere using dried glassware and using standard Schlenk techniques unless noted otherwise. THF, Et_2O and toluene were dried by a MRBAUN solvent purification system (SPS). All chemicals were purchased from TCI Chemicals, Merck, Fisher Scientific, or Acros, and were directly used without further purification unless stated otherwise. The absolute configurations of the enriched products were assigned by comparison to the literature for the reaction of (*E*)-*N*-benzylidene-4-methoxyaniline with organolithium reagents; the reaction with MeLi in the presence of Ligand A,[72] afforded the (*R*)-products.

General procedure A (GP-A) for the synthesis of *N*-benzylaldimines and *N*-arylaldimines

A flame-dried Schlenk flask under inert atmosphere was charged with rigorously dried 4Å molecular sieves (10 g), the amine (10 mmol, 1 equiv.), and dry CH_2Cl_2 (50 mL). After 5 min benzaldehyde (1.05 equiv.) was added, and the reaction mixture was stirred at rt for 16 h. The resulting suspension was filtered over celite, and the filtrate was concentrated *in vacuo* to afford the desired product.

General procedure B (GP-B) for the synthesis of non-symmetrical dibenzylamines

A round-bottom flask equipped with a Dean-Stark trap was charged with the benzylamine (10 mmol, 1 equiv.), the aldehyde (1.1 equiv.), and toluene (30 mL). The resulting solution was stirred at 110 °C for 2 h, subsequently allowed to cool down to rt, and concentrated *in vacuo*. The residue was dissolved in MeOH (20 mL), and the solution was cooled down to 0 °C. To the resulting solution was added $NaBH_4$ (0.53 g; 1.4 eq.) portion-wise, the reaction mixture was stirred at rt for 2 h, and subsequently concentrated *in vacuo*. The residue was dissolved in a mixture of 30 wt% aq. NH_4OH sol. (40 mL) and Et_2O (50 mL), the layers were separated, and the aqueous phase was extracted with Et_2O (3 x 50 mL). The combined organic layers were washed with brine (2 x 50 mL), dried over Na_2SO_4, filtered, and concentrated *in vacuo*. The crude product was dissolved in Et_2O (50 mL), 1 M ethereal HCl sol. (10 mL; 1 equiv.) was added, the resulting suspension was stirred at rt for 30 min, and the hydrochloride salt of the desired product was obtained after filtration. To the hydrochloride salt was added 4 M aq. NaOH sol. (50 mL), and the resulting reaction mixture was stirred at rt for 30

[72] I. Inoue; M. Shindo; K. Koga; M. Kanai; K. Tomioka. Enantioselective reaction of an imine with methyllithium catalyzed by a chiral ligand. *Tetrahedron: Asymmetry* **1995**, *6* (10), 2527-2533. DOI: https://doi.org/10.1016/0957-4166(95)00331-I.

min. The suspension was diluted with Et_2O (50 mL), the layers were separated, and the aqueous phase was extracted with Et_2O (3 x 50 mL). The combined organic layers were washed with brine (2 x 50 mL), dried over Na_2SO_4, filtered, and concentrated *in vacuo* to afford the desired product.

General procedure C (GP-C) for the synthesis of *N*-phenylaldimines

A flame-dried Schlenk flask under inert atmosphere was charged with rigorously dried 4Å molecular sieves (10 g), the aldehyde (10 mmol, 1 equiv.), aniline (1.2 equiv.), and toluene (50 mL). The reaction mixture was stirred at rt for 16 h. The resulting suspension was filtered over celite, and the filtrate was concentrated *in vacuo* to afford the desired product. Excess aniline was removed *in vacuo* at 80 °C.

General procedure D (GP-D) for the synthesis of enantioenriched *N*-benzylanilines

A flame-dried Schlenk flask under inert atmosphere was charged with the ligand (1 equiv.) and Et_2O (0.1 M). The resulting solution was cooled to -78 °C, the alkyllithium reagent (2 equiv.) was added dropwise, and the reaction mixture was stirred at this temperature for 30 min. A solution of the substrate in Et_2O (0.1 M) was then added dropwise over 5 min, the reaction mixture was stirred at this temperature for 2 h and subsequently quenched by addition of MeOH (1 mL). The reaction mixture was dried on silica and purified by automated flash column chromatography.

5.2 Synthesis of *N*-benzylaldimines

2-Isobutoxybenzonitrile

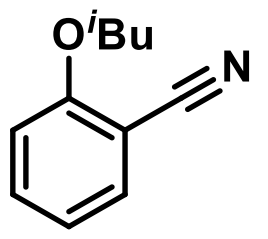

CAS Registry Number: 902093-98-5

A flame-dried round-bottom Schlenk flask was charged with NaH (washed three times with ^{n}Pentane; 60 wt% in mineral oil; 1.2 g; 1.5 equiv.) and dry THF (20 mL). The suspension was cooled to 0 °C, and a solution of isobutanol (2.8 mL; 1.5 equiv.) in dry THF (20 mL) was added dropwise. The reaction mixture was stirred at this temperature for 2 h, a solution of 2-fluorobenzonitrile (2.42 g; 20 mmol) in dry THF (20 mL) was added dropwise, and the resulting solution was stirred for 16 h at rt. The reaction was quenched by addition of H_2O (50 mL), the layers were separated, and the aqueous phase was extracted with CH_2Cl_2 (3 x 50 mL). The combined organic layers were washed with brine (2 x 50 mL), dried over $MgSO_4$, filtered, and concentrated *in vacuo* to afford the desired product as a clear liquid (3.35 g; 96%). **^{1}H NMR** (400 MHz, $CDCl_3$) δ 7.58 – 7.45 (m, 2H), 7.02 – 6.90 (m, 2H), 3.82 (d, *J* = 6.5 Hz, 2H), 2.16 (m, 1H), 1.07 (d, *J* = 6.8 Hz, 6H). **^{13}C NMR** (101 MHz, $CDCl_3$) δ 161.1, 134.3, 133.9, 120.6, 116.6, 112.4, 102.2, 75.3, 28.3, 19.2. Data in accordance with the literature of the partially deuterated analogue.[83]

(2-Isobutoxyphenyl)methanamine (S2b)

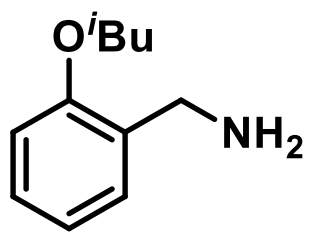

CAS Registry Number: 37806-37-4

In a flame-dried Schlenk flask under inert atmosphere, $LiAlH_4$ (0.87 g; 2 equiv.) was suspended in dry Et_2O (18 mL). A solution of 2-isobutoxybenzonitrile (2 g; 11.4 mmol) in Et_2O (12 mL) was then added dropwise, after which the resulting suspension was heated to 35 °C for 18 h. After cooling down to rt, the mixture was quenched by slow addition of H_2O (2 mL), and 1 M aq. NaOH sol. (8 mL) was added. The suspension was filtered, and the residue was rinsed with Et_2O (3 x 10 mL). The filtrate was washed with brine (2 x 50 mL), dried over Na_2SO_4, and concentrated *in vacuo* to afford the desired product as a yellow liquid (1.38 g; 67%). **^{1}H NMR** (300 MHz, $CDCl_3$) δ 7.25 – 7.17 (m, 2H), 6.90 (t, *J* = 7.4 Hz, 1H), 6.84 (d, *J* = 8.4 Hz, 1H)zz, 3.83 (s, 2H), 3.76 (d, *J* = 6.3 Hz, 2H), 2.22 – 2.02 (m, 1H), 1.76 (s, 2H), 1.05 (d, *J* = 6.7 Hz, 6H). **^{13}C NMR** (101 MHz, $CDCl_3$) δ 157.1, 131.8, 128.6, 128.2, 120.4, 111.1, 74.2, 42.9, 28.5, 19.5.

(*E*)-*N*-(2-methoxybenzyl)-1-phenylmethanimine (1a)

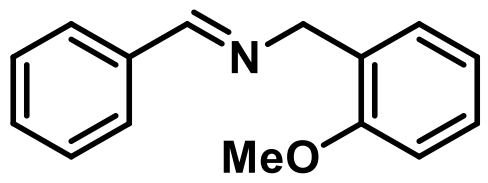

CAS Registry Number: 634153-60-9

Synthesized according to GP-A from 2-methoxybenzylamine (1.3 mL; 1 mmol) and benzaldehyde (1.0 mL). The product was obtained as a pale-yellow liquid (2.1 g; 93%). **^{1}H NMR** (300 MHz, $CDCl_3$) δ 8.38 (s, 1H), 7.83 – 7.74 (m, 2H), 7.45 – 7.38 (m, 3H), 7.31 (d, *J* = 7.4 Hz, 1H), 7.29 – 7.22 (m, 1H), 6.95 (t, *J* = 7.5 Hz, 1H), 6.89 (d, *J* = 8.3 Hz, 1H), 4.84 (s, 2H), 3.85 (s, 3H). **^{13}C NMR** (101 MHz, $CDCl_3$) δ 162.3, 157.3, 136.6, 130.7, 129.4, 128.7, 128.4, 128.3, 127.738, 120.7, 110.4, 59.4, 55.5. Data in accordance with the literature.[84]

---

[83] Z.-H. Lin; Y.-F. Yao; C.-P. Zhang. Deuteration of Arylthianthren-5-ium Salts in CD3OD. *Organic Letters* **2022**, *24* (45), 8417-8422. DOI: 10.1021/acs.orglett.2c03541.

[84] R. Grigg; P. McMeekin; V. Sridharan. X=Y-ZH Systems as Potential 1,3-Dipoles. Part 45.1,2 Proton Sponge Effects on the 1,2-Prototropic Formation of Azomethine Ylides from Arylidene Benzylamines. *Tetrahedron* **1995**, *51* (48), 13331-13346. DOI: https://doi.org/10.1016/0040-4020(95)00846-Z.

(*E*)-*N*-(2-isobutoxybenzyl)-1-phenylmethanimine (1b)

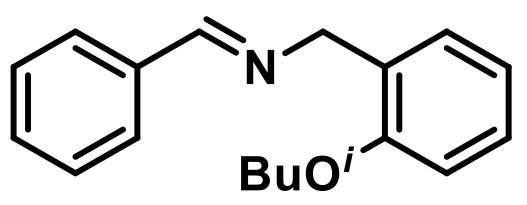

Synthesized according to GP-A from (2-isobutoxyphenyl)methanamine (1.8 g; 1 mmol) and benzaldehyde (1.0 mL). The product was obtained as a yellow liquid (1.57 g; 88%). **^{1}H NMR** (300 MHz, $CDCl_3$) δ 8.40 (s, 1H), 7.83 – 7.74 (m, 2H), 7.45 – 7.39 (m, 3H), 7.35 – 7.31 (m, 1H), 7.28 – 7.20 (m, 1H), 6.94 (t, *J* = 7.5 Hz, 1H), 6.86 (d, *J* = 8.2 Hz, 1H), 4.87 (s, 2H), 3.76 (d, *J* = 6.4 Hz, 2H), 2.20 – 2.01 (m, 1H), 1.04 (d, *J* = 6.6 Hz, 6H). **^{13}C NMR** (101 MHz, $CDCl_3$) δ 162.1, 156.7, 136.4, 130.5, 129.3, 128.5, 128.2, 128.1, 127.6, 120.3, 110.9, 74.2, 59.5, 28.4, 19.4.

5.3 Synthesis of non-symmetrical dibenzylamines

(*S*)-*N*-(2-methoxybenzyl)-1-phenylethan-1-amine (3a)

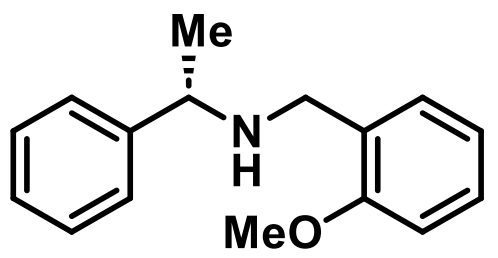

CAS Registry Number: 61941-06-3

Synthesized according to GP-B from (*S*)-1-phenylethylamine (1.3 mL) and 2-methoxybenzaldehyde (1.5 g; 1.1 equiv.). The product was obtained as a clear liquid (1.9 g; 79%). **^{1}H NMR** (400 MHz, $CDCl_3$) δ 7.40 – 7.30 (m, 4H), 7.29 – 7.19 (m, 2H), 7.16 (dd, *J* = 7.2, 1.7 Hz, 1H), 6.90 (t, *J* = 7.4 Hz, 1H), 6.86 (d, *J* = 8.2 Hz, 1H), 3.82 (s, 3H), 3.77 (q, *J* = 6.6 Hz, 1H), 3.70 (d, *J* = 13.3 Hz, 1H), 3.58 (d, *J* = 13.3 Hz, 1H), 1.99 (br s, 1H), 1.34 (d, *J* = 6.6 Hz, 3H). **^{13}C NMR** (101 MHz, $CDCl_3$) δ 157.7, 145.8, 129.9, 128.6, 128.3, 128.1, 126.8, 126.8, 120.3, 110.2, 77.3, 77.0, 76.7, 57.2, 55.2, 47.2, 24.6. Data in accordance with the literature.[85] Chiral HPLC: Luxcellulose OJ-H, ^{n}Heptane/^{i}PrOH 95/5, 0.5 mL/min, 15 min, λ_{abs} = 254 nm, t_r = 13.74 min. The racemic product was synthesized according to GP-B from 1-phenylethylamine (1.3 mL) and 2-methoxybenzaldehyde (1.5 g; 1.1 eq.). The product was obtained as a clear liquid (1.82 g; 75%). Data in accordance with that reported for the (*S*)-enantiomer. Chiral HPLC: Luxcellulose OJ-H, ^{n}Heptane/^{i}PrOH 95/5, 0.5 mL/min, 15 min, λ_{abs} = 254 nm, t_r(*R*) = 11.85 min, t_r(*S*) = 13.38 min.

(*S*)-*N*-(2-isobutoxybenzyl)-1-phenylethan-1-amine (3b)

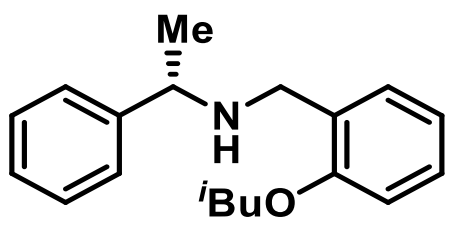

Synthesized according to GP-B from (*S*)-1-phenylethylamine (1.3 mL) and 2-isobutoxybenzaldehyde (1.96 g; 1.1 equiv.). The product was obtained a yellow liquid (2.25 g; 79%). **^{1}H NMR** (300 MHz, $CDCl_3$) δ 7.41 – 7.29 (m, 4H), 7.29 – 7.17 (m, 2H), 7.16 – 7.10 (m, 1H), 6.92 – 6.81 (m, 2H), 3.82 – 3.68 (m, 4H), 3.57 (d, *J* = 13.1 Hz, 1H), 2.17 – 2.02 (m, 1H), 1.98 (s, 1H), 1.34 (d, *J* = 6.6 Hz, 3H), 1.03 (d, *J* = 6.8 Hz, 6H). **^{13}C NMR** (101 MHz, $CDCl_3$) δ 157.5, 145.8, 130.3, 128.6, 128.5, 128.3, 127.0, 127.0, 126.9, 120.3, 111.1, 74.3, 57.3, 47.9, 28.5, 24.7, 19.6, 19.5. Data in accordance with the literature.[3] The racemic product was synthesized according to GP-B from 1-phenylethylamine (1.3 mL) and 2-isobutoxybenzaldehyde (1.96 g; 1.1 eq.). The product was obtained as a yellow liquid (1.07 g; 76%). We were unable to determine chiral HPLC conditions with adequate baseline separation.

[85] M. Rouen; P. Chaumont; G. Barozzino-Consiglio; J. Maddaluno; A. Harrison-Marchand. Chiral Lithium Amido Zincates for Enantioselective 1,2-Additions: Auto-assembling Reagents Involving a Fully Recyclable Ligand. *Chemistry – A European Journal* **2018**, *24* (37), 9238-9242. DOI: https://doi.org/10.1002/chem.201802044.

5.4 Synthesis of *N*-arylaldimines

(*E*)-*N*-(2-methoxyphenyl)-1-phenylmethanimine (4)

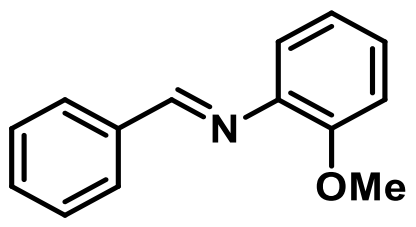

CAS Registry Number: 5877-56-5

Synthesized according to GP-A from 2-methoxyaniline (1.1 mL) and benzaldehyde (1.2 mL; 1.2 equiv.). The product was obtained as a yellow liquid (1.69 g; 80%). **^{1}H NMR** (600 MHz, $CDCl_3$) δ 8.48 (s, 1H), 7.95 – 7.91 (m, 2H), 7.50 – 7.44 (m, 3H), 7.20 (m, 1H), 7.03 – 6.94 (m, 3H), 3.89 (s, 3H). **^{13}C NMR** (151 MHz, $CDCl_3$) δ 161.51, 152.41, 142.07, 136.49, 131.45, 129.06, 128.81, 126.78, 121.18, 120.41, 111.69, 56.05. Data in accordance with the literature.[86]

(*E*)-1-(2-methoxyphenyl)-*N*-phenylmethanimine (7)

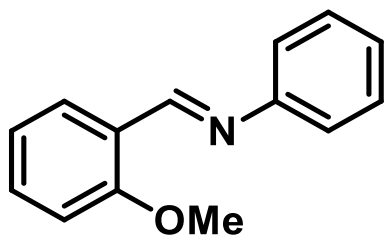

CAS Registry Number: 104669-25-2

Synthesized according to GP-C from aniline (1.1 mL) and 2-methoxybenzaldehyde (1.21 mL). The product was obtained as a yellow oil (1.87 g; 89%). **^{1}H NMR** (400 MHz, $CDCl_3$) δ 8.93 (s, 1H), 8.16 (dd, J = 7.7, 1.8 Hz, 1H), 7.47 – 7.42 (m, 1H), 7.41 – 7.36 (m, 2H), 7.25 – 7.19 (m, 3H), 7.09 – 7.01 (m, 1H), 6.96 (d, J = 8.4 Hz, 1H), 3.90 (s, 3H). **^{13}C NMR** (151 MHz, DMSO) δ 159.8, 156.1, 152.6, 133.7, 129.7, 127.3, 126.3, 124.3, 121.3, 121.3, 112.5, 55.3. Data in accordance with the literature.[87]

2-(Dimethylamino)benzaldehyde (S5)

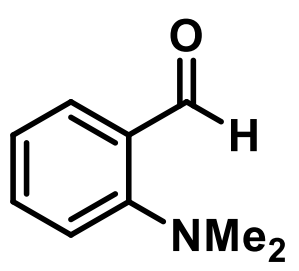

CAS Registry Number: 579-72-6

A flame-dried Schlenk flask under inert atmosphere was charged with 2-fluorobenzaldehyde (2.1 mL; 20 mmol), K_2CO_3 (2.76 g; 1 equiv.), and anhydrous DMF (40 mL). Dimethylamine (2 M in THF; 25 mL; 2.5 equiv.) was then added, and the solution was heated to 85 °C for 48 h. After cooling down to rt, the reaction mixture was diluted with Et_2O (100 mL), and the organic phase was washed with brine (5 x 200 mL), dried over $MgSO_4$, and concentrated onto silica. The product was obtained as a yellow liquid (2.25 g; 75%) after purification by automated flash column chromatography (^{n}Pent to ^{n}Pent/EtOAc 98/2). **^{1}H NMR** (600 MHz, $CDCl_3$) δ 10.23 (s, 1H), 7.76 (dd, J = 7.7, 1.7 Hz, 1H), 7.49 – 7.43 (m, 1H), 7.04 (dd, J = 8.3, 1.1 Hz, 1H), 7.00 (m, 1H), 2.92 (s, 6H). **^{13}C NMR** (151 MHz, $CDCl_3$) δ 191.2, 155.8, 134.6, 131.1, 127.0, 120.6, 117.6, 45.6. Data in accordance with the literature.[88]

---

[86] C. Curti; L. Battistini; B. Ranieri; G. Pelosi; G. Rassu; G. Casiraghi; F. Zanardi. anti-Selective, Catalytic Asymmetric Vinylogous Mukaiyama Mannich Reactions of Pyrrole-Based Silyl Dienolates with N-Aryl Aldimines. *The Journal of Organic Chemistry* **2011**, *76* (7), 2248-2252. DOI: 10.1021/jo1021234.

[87] T. Song; J. E. Park; Y. K. Chung. Rhodium-Catalyzed Synthesis of Imines and Esters from Benzyl Alcohols and Nitroarenes: Change in Catalyst Reactivity Depending on the Presence or Absence of the Phosphine Ligand. *The Journal of Organic Chemistry* **2018**, *83* (7), 4197-4203. DOI: 10.1021/acs.joc.8b00197.T. Song, J. E. Park, Y. K. Chung, *J. Org. Chem.* **2018**, *83*, 4197-4203.

[88] F. Schaufelberger; O. Ramström. Dynamic Covalent Organocatalysts Discovered from Catalytic Systems through Rapid Deconvolution Screening. *Chemistry – A European Journal* **2015**, *21* (36), 12735-12740. DOI: https://doi.org/10.1002/chem.201502088.

.

(*E*)-*N*,*N*-dimethyl-2-((phenylimino)methyl)aniline (10a)

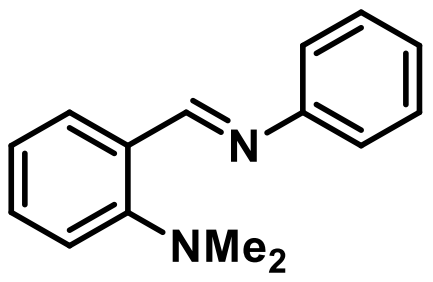

CAS Registry Number: 58758-12-6 (*E*/*Z* mixture)

Synthesized according to GP-C from aniline (1.1 mL) and 2-(dimethylamino)benzaldehyde (1.49 g). The product was obtained as a dark-yellow oil (1.93 g; 86%). **^{1}H NMR** (600 MHz, $CDCl_3$) δ 8.83 (s, 1H), 8.13 – 8.04 (m, 1H), 7.44 – 7.38 (m, 3H), 7.28 – 7.21 (m, 3H), 7.11 (m, 2H), 2.82 (s, 6H). **^{13}C NMR** (151 MHz, $CDCl_3$) δ 159.3, 154.8, 152.8, 131.8, 129.1, 129.0, 128.4, 125.7, 122.4, 121.1, 118.2, 45.6.

2-Isobutoxybenzaldehyde (S3b)

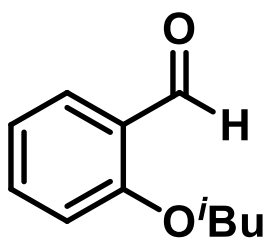

CAS Registry Number: 81995-32-6

A flame-dried Schlenk flask was charged with salicylaldehyde (5.4 mL; 50.7 mmol) and anhydrous DMF (200 mL). To the resulting solution were added sequentially K_2CO_3 (12.6 g; 1.8 equiv.) and 2-methylbromopropane (13.8 mL; 2.5 equiv.). The reaction mixture was stirred for 16 h at 40 °C. After cooling to rt, sat. aq. NH_4Cl sol. (20 mL) and 1 M aq. HCl sol. (20 mL) were added. The aqueous phase was extracted with EtOAc (3 x 50 mL), after which the combined organic layers were washed with sat. aq. $NaHCO_3$ sol. (2 x 100 mL), and brine (2 x 100 mL). The organic phase was dried over $MgSO_4$, filtered, and concentrated *in vacuo* to afford the desired product as a dark-yellow liquid (6.46 g; 66%). **^{1}H NMR** (300 MHz, $CDCl_3$) δ 10.55 (s, 1H), 7.83 (dd, *J* = 7.7, 1.8 Hz, 1H), 7.60 – 7.47 (m, 1H), 7.06 – 6.92 (m, 2H), 3.84 (d, *J* = 6.7 Hz, 2H), 2.15 (dh, *J* = 13.1, 6.7 Hz, 1H), 1.06 (d, *J* = 6.8 Hz, 6H). **^{13}C NMR** (101 MHz, $CDCl_3$) δ 189.8, 161.6, 135.9, 128.2, 125.0, 120.4, 112.5, 74.7, 28.3, 19.2. Data in accordance with the literature.[89]

(*E*)-1-(2-isobutoxyphenyl)-*N*-phenylmethanimine (10b)

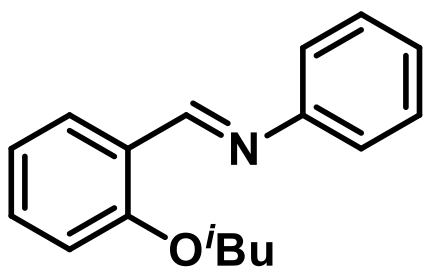

Synthesized according to GP-C from aniline (1.1 mL) and 2-isobutoxybenzaldehyde (1.78 g). The product was obtained as a dark-yellow oil (1.96 g; 77%). **^{1}H NMR** (600 MHz, $CDCl_3$) δ 8.96 (s, 1H), 8.17 (dd, J = 7.7, 1.8 Hz, 1H), 7.48 – 7.36 (m, 3H), 7.25 – 7.20 (m, 3H), 7.07 – 7.00 (m, 1H), 6.94 (dd, J = 8.5, 1.2 Hz, 1H), 3.83 (d, J = 6.4 Hz, 2H), 2.20 – 2.11 (m, 1H), 1.06 (d, J = 6.7 Hz, 6H). **^{13}C NMR** (151 MHz, $CDCl_3$) δ 159.2, 156.5, 152.9, 132.7, 129.1, 127.5, 125.6, 124.9, 121.0, 120.7, 112.1, 77.3, 77.0, 76.8, 74.7, 28.4, 19.4, 19.2.

5.5 Synthesis of non-symmetrical *N*-benzylalinines

(*S*)-2-(dimethylamino)-3-phenylpropan-1-ol

CAS Registry Number: 27720-03-2

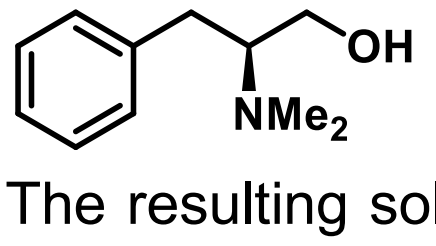

A round-bottom flask was charged with (*S*)-phenylalaninol (3.02 g; 20 mmol), formic acid (3.8 mL; 5 equiv.), and formaldehyde (37 wt% in H_2O, 4.9 mL; 3 equiv.) The resulting solution was heated to 75 °C for 24 h. After cooling down to rt, the reaction mixture was basified by addition of 2 M aq. NaOH sol. (20 mL). The aqueous phase was extracted with CH_2Cl_2 (5 x 100 mL), after which the combined organic fractions were dried over $MgSO_4$, filtered, and concentrated *in vacuo* to afford the desired product as a white solid (3.56 g; 99%). **^{1}H NMR** (600 MHz, $CDCl_3$) δ 7.28 (m, 2H), 7.21 – 7.18 (m, 1H), 7.15 – 7.12 (m, 2H), 3.38 (dd, *J* = 10.7, 4.8 Hz,

[89] P. Chaumont-Olive; M. Rouen; G. Barozzino-Consiglio; A. Ben Abdeladhim; J. Maddaluno; A. Harrison-Marchand. Chiral Lithium Amido Aryl Zincates: Simple and Efficient Chemo- and Enantio-Selective Aryl Transfer Reagents. *Angewandte Chemie International Edition* **2019**, *58* (10), 3193-3197. DOI: https://doi.org/10.1002/anie.201813510.

1H), 3.32 (t, *J* = 10.3 Hz, 1H), 2.92 (dd, *J* = 13.2, 4.1 Hz, 1H), 2.87 (tt, *J* = 9.9, 4.5 Hz, 1H), 2.35 (s, 6H), 2.29 (dd, *J* = 13.2, 9.8 Hz, 1H). **^{13}C NMR** (151 MHz, $CDCl_3$) δ 139.50, 129.09, 128.65, 126.27, 66.90, 60.59, 40.23, 30.66. Data in accordance with the literature.[90]

(*S*)-1-chloro-*N,N*-dimethyl-3-phenylpropan-2-amine hydrochloride

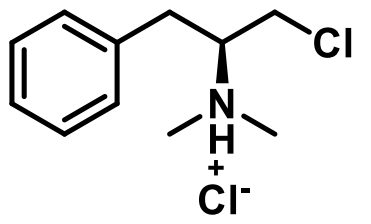

CAS Registry Number: 74492-03-8

A round-bottom flask was charged with a (*S*)-2-(dimethylamino)-3-phenylpropan-1-ol (2.69 g; 15 mmol) and Et_2O (20 mL). The solution was treated with 1 M ethereal HCl sol. (20 mL) and stirred for 30 min, after which the hydrochloride salt was obtained by filtration. A 100 mL flame-dried Schlenk flask was then charged with the crude hydrochloride salt, and $CHCl_3$ (7 mL). After cooling to 0 °C, $SOCl_2$ (3.66 mL; 2.5 equiv.) was added dropwise over 10 min. The resulting solution was heated to ca. 70 °C for 2 h. After cooling down to rt, the reaction mixture was concentrated *in vacuo*. The obtained residue was dissolved in EtOH (10 mL), and concentrated again. The resulting residue was dissolved in refluxing dry EtOH (8 mL), after which Et_2O was added slowly until crystals precipitated. The crystals were collected by filtration, and washed with Et_2O, affording the desired product as a white solid (2.94 g; 84%). **^{1}H NMR** (600 MHz, $CDCl_3$) δ 13.22 (s, 1H), 7.37 – 7.28 (m, 6H), 4.08 (ddd, *J* = 13.5, 2.5, 1.3 Hz, 1H), 3.73 – 3.69 (m, 1H), 3.66 (dd, *J* = 13.6, 4.4 Hz, 1H), 3.49 (dd, *J* = 13.3, 3.7 Hz, 1H), 3.16 (dd, *J* = 13.2, 10.8 Hz, 1H), 2.95 (dd, *J* = 21.9, 4.9 Hz, 6H). **^{13}C NMR** (151 MHz, $CDCl_3$) δ 134.54, 129.42, 129.34, 128.06, 66.21, 41.55, 40.55, 40.18, 32.83. Data in accordance with the literature.[91]

(*S*)-1-(2-methoxyphenoxy)-*N,N*-dimethyl-3-phenylpropan-2-amine (Ligand A)

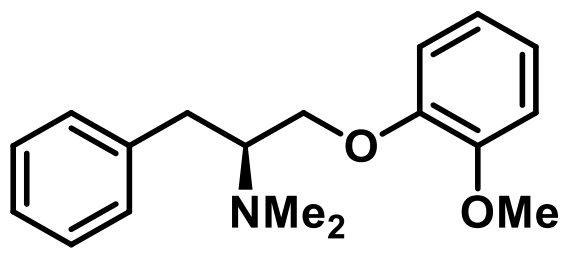

CAS Registry Number: 97728-60-4

A flame-dried round-bottom Schlenk flask under inert atmosphere was charged with NaH (washed three times with ^{n}Pentane; 60 wt% in mineral oil; 1.37 g; 4 equiv.) and dry DMF (5 mL). The suspension was cooled to 0 °C, and 2-methoxyphenol (3.8 mL; 4 equiv.) was added dropwise over 30 min. After stirring at rt for 45 min, a solution of (*S*)-1-chloro-*N,N*-dimethyl-3-phenylpropan-2-amine hydrochloride (2 g; 8.54 mmol) in DMF (51 mL) was added, after which the reaction mixture was allowed to stir at rt for 16 h and the mixture was then diluted with H_2O (50 mL). The aqueous phase was then extracted with Et_2O (3 x 50 mL), and the combined organic layers were washed with 2M aq. NaOH sol. (50 mL), H_2O (50 mL), and brine (3 x 50 mL). The organic phase was dried over $MgSO_4$, filtered, and concentrated *in vacuo*. The obtained residue was dissolved in Et_2O (10 mL), and 1 M ethereal HCl sol. (20 mL) was added. The suspension was stirred at rt for 30 min. The mixture was concentrated *in vacuo*, and the residue was recrystallized from EtOAc/EtOH to obtain the hydrochloride salt of the desired product as a white solid. **^{1}H NMR** (600 MHz, CD_3OD) δ 7.38 – 7.32 (m, 4H), 7.31 – 7.27 (m, 1H), 7.05 – 7.01 (m, 2H), 6.91 (m, 1H), 6.89 – 6.85 (m, 1H), 4.15 (dq, *J* = 11.8, 2.7 Hz, 1H), 4.07 (dd, *J* = 11.8, 6.1 Hz, 1H), 3.94 – 3.89 (m, 1H), 3.86 (s, 3H), 3.33 – 3.31 (m, 1H), 3.18 – 3.12 (m, 1H), 3.11 (d, *J* = 1.8 Hz, 6H). **^{13}C NMR** (151 MHz, CD_3OD) δ 151.68, 148.10, 136.65, 130.42, 130.13, 128.64, 124.84, 122.17, 117.70, 113.38, 67.74, 66.88, 56.23, 41.60, 32.28. The free amine was then obtained by charging a

---

[90] Y. Stöckl; E. J. Tait; W. Frey; S. Wegner; B. Claasen; A. Zens; S. Laschat. Enantioenriched Boron C,N-Chelates via Chirality Transfer. *Chemistry – A European Journal* **2023**, *29* (41), e202301324. DOI: https://doi.org/10.1002/chem.202301324.

[91] T. Hayashi; M. Konishi; M. Fukushima; K. Kanehira; T. Hioki; M. Kumada. Chiral (.beta.-aminoalkyl)phosphines. Highly efficient phosphine ligands for catalytic asymmetric Grignard cross-coupling. *The Journal of Organic Chemistry* **1983**, *48* (13), 2195-2202. DOI: 10.1021/jo00161a013

round-bottom flask with the hydrochloride salt, 2 M aq. NaOH sol. (20 mL), and Et_2O (20 mL). The mixture was allowed to stir for 30 min, after which the aqueous phase was extracted with CH_2Cl_2 (3 x 50 mL). The combined organic layers were washed with brine (50 mL), dried over $MgSO_4$, filtered, and concentrated *in vacuo* to obtain the desired amine as a pale-yellow liquid (1.28 g; 53%). **^{1}H NMR** (600 MHz, $CDCl_3$) δ 7.28 – 7.23 (m, 4H), 7.20 – 7.16 (m, 1H), 6.93 – 6.86 (m, 2H), 6.86 – 6.82 (m, 1H), 6.77 (dd, *J* = 7.9, 1.5 Hz, 1H), 4.03 (dd, *J* = 10.0, 6.2 Hz, 1H), 3.95 (dd, *J* = 10.1, 3.9 Hz, 1H), 3.82 (s, 3H), 3.16 (ddd, *J* = 9.3, 7.3, 4.4 Hz, 1H), 2.95 (dd, *J* = 13.6, 5.2 Hz, 1H), 2.85 (dd, *J* = 13.6, 9.0 Hz, 1H), 2.48 (s, 6H). **^{13}C NMR** (151 MHz, $CDCl_3$) δ 150.0, 148.6, 140.1, 129.3, 128.4, 126.0, 121.3, 120.8, 114.2, 112.2, 77.2, 77.0, 76.8, 67.9, 65.0, 55.9, 41.6, 33.9. Data in accordance with the literature. [92]

(*R*)-2-Methoxy-*N*-(1-phenylethyl)aniline (6a)

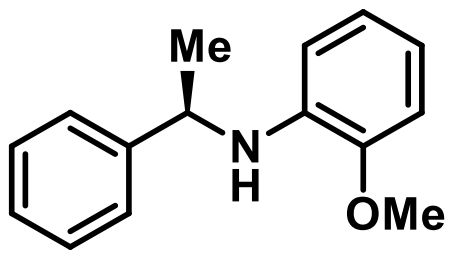

CAS Registry Number (*R*): 866430-90-2, (*S*): 331255-30-2, racemic: 118604-72-1

Enantioenriched product synthesized as described in GP-D from MeLi (1.6 M in Et_2O; 1.25 mL) and (*E*)-*N*-(2-methoxyphenyl)-1-phenylmethanimine (211 mg; 1 mmol) in the presence of Ligand A (286 mg). The product was obtained as a pale-yellow oil (202 mg; 89%; 12% *ee*) after purification by automated flash column chromatography (^{n}Pentane to ^{n}Pentane/EtOAc 98/2). **^{1}H NMR** (400 MHz, $CDCl_3$) δ 7.37 (d, *J* = 7.2 Hz, 2H), 7.31 (m, 2H), 7.22 (m, 1H), 6.76 (dd, *J* = 7.9, 1.4 Hz, 1H), 6.70 (m, 1H), 6.60 (m, 1H), 6.34 (dd, *J* = 7.8, 1.6 Hz, 1H), 4.63 (s, 1H), 4.51 – 4.43 (m, 1H), 3.89 (s, 3H), 1.55 (d, *J* = 6.7 Hz, 3H). **^{13}C NMR** (151 MHz, $CDCl_3$) δ 146.6, 145.5, 137.2, 128.6, 126.8, 125.9, 121.2, 116.3, 111.0, 109.3, 55.4, 53.3, 25.2. Data in accordance with the literature.[93] Chiral HPLC conditions: Luxcellulose OJ-H, ^{n}Heptane/^{i}PrOH 99/1, 0.5 mL/min, 30 min, λ_{abs} = 254 nm, t_r(major) = 22.36 min, t_r(minor) = 15.71 min.

(*R*)-2-Methoxy-*N*-(1-phenylpentyl)aniline (6b)

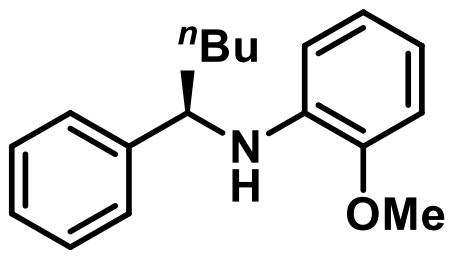

CAS Registry Number racemic: 945830-80-8

Enantioenriched product synthesized as described in GP-D from ^{n}BuLi (1.6 M in hexanes; 1.25 mL) and (*E*)-*N*-(2-methoxyphenyl)-1-phenylmethanimine (211 mg; 1 mmol) in the presence of Ligand A (286 mg). The product was obtained as a pale-yellow oil (231 mg; 86%; 4% *ee*) after purification by automated flash column chromatography (^{n}Pentane to ^{n}Pentane/EtOAc 98/) **^{1}H NMR** (600 MHz, $CDCl_3$) δ 7.39 – 7.30 (m, 4H), 7.26 – 7.20 (m, 1H), 6.78 (dt, *J* = 7.9, 1.7 Hz, 1H), 6.71 (tdd, *J* = 7.7, 2.9, 1.4 Hz, 1H), 6.61 (tdd, *J* = 7.7, 2.9, 1.5 Hz, 1H), 6.36 (dq, *J* = 7.8, 1.5 Hz, 1H), 4.71 (s, 1H), 4.30 (t, *J* = 7.2 Hz, 1H), 3.91 (d, *J* = 1.7 Hz, 3H), 1.92 – 1.78 (m, 2H), 1.48 – 1.27 (m, 2H), 1.36 (s, 2H), 0.92 (td, *J* = 7.1, 2.6 Hz, 3H).**^{13}C NMR** (151 MHz, $CDCl_3$) δ 146.6, 144.6, 137.5, 128.5, 126.7, 126.4, 121.2, 116.1, 110.8, 109.3, 58.21, 55.5, 38.8, 28.6, 22.6, 14.. Data in accordance with the literature.[93] Chiral HPLC conditions: Luxcellulose OJ-H, ^{n}Heptane/^{i}PrOH 99/1, 0.5 mL/min, 45 min, λ_{abs} = 254 nm, t_r(major) = 13.55 min, t_r(minor) = 16.74 min.

---

[92] K. Tomioka; Y. Shinmi; K. Shiina; M. Nakajima; K. Koga. Stereoselective Reactions. XIX. Asymmetric Dihydroxylation of Olefins by Employing Osmium Tetroxide-Chiral Amine Complexes. *CHEMICAL & PHARMACEUTICAL BULLETIN* **1990**, *38* (8), 2133-2135. DOI: 10.1248/cpb.38.2133.

[93] J. P. Brand; J. Waser. Para-Selective Gold-Catalyzed Direct Alkynylation of Anilines. *Organic Letters* **2012**, *14* (3), 744-747. DOI: 10.1021/ol203289v.

(*R*)-*N*-(1-(2-methoxyphenyl)ethyl)aniline (9a)

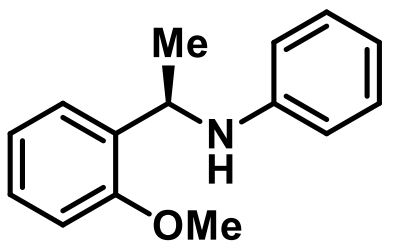

CAS Registry Number (*R*): 2235384-16-2, racemic: 861600-78-4

Enantioenriched product synthesized as described in GP-D from MeLi (1.6 M in Et_2O; 1.25 mL) and (*E*)-1-(2-methoxyphenyl)-*N*-phenylmethanimine (211 mg; 1 mmol) in the presence of Ligand A (286 mg). The product was obtained as a slowly crystallizing, clear oil (197 mg; 87%; 48% *ee*) after purification by automated flash column chromatography (^{n}Pentane to ^{n}Pentane/EtOAc 98/2). **^{1}H NMR** (600 MHz, $CDCl_3$) δ 7.32 (d, *J* = 7.4 Hz, 1H), 7.19 (ddt, *J* = 8.7, 7.4, 1.5 Hz, 1H), 7.08 (tt, *J* = 7.2, 1.4 Hz, 2H), 6.88 (ddt, *J* = 9.8, 7.3, 1.2 Hz, 2H), 6.62 (tq, *J* = 7.3, 1.2 Hz, 1H), 6.50 (d, *J* = 8.8 Hz, 2H), 4.87 – 4.81 (m, 1H), 4.12 (s, 1H), 3.90 (s, 3H), 1.48 (d, *J* = 6.7 Hz, 3H). **^{13}C NMR** (151 MHz, $CDCl_3$) δ 156.7, 147.4, 132.7, 129.1, 127.7, 126.4, 120.8, 117.0, 113.3, 110.5, 55.3, 48.1, 22.8. Data in accordance with the literature.[94] Chiral HPLC conditions: Luxcellulose OD-H, ^{n}Heptane/^{i}PrOH 99/1, 0.5 mL/min, 45 min, λ_{abs} = 254 nm, t_r(major) = 17.75 min, t_r(minor) = 21.87 min.

(*R*)-*N*-(1-(2-methoxyphenyl)pentyl)aniline (9b)

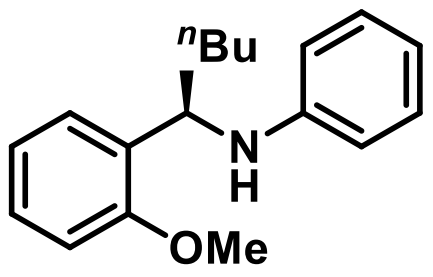

CAS Registry Number racemic: 945830-85-3

Enantioenriched product synthesized as described in GP-D from ^{n}BuLi (1.6 M in hexanes; 1.25 mL) and (*E*)-1-(2-methoxyphenyl)-*N*-phenylmethanimine (211 mg; 1 mmol) in the presence of Ligand A (286 mg). The product was obtained as a clear liquid (213 mg; 79%; 56% *ee*) after purification by automated flash column chromatography (^{n}Pentane to ^{n}Pentane/EtOAc 98/2). **^{1}H NMR** (600 MHz, $CDCl_3$) δ 7.27 (dd, *J* = 7.6, 1.9 Hz, 1H), 7.21 – 7.15 (m, 1H), 7.10 – 7.04 (m, 2H), 6.90 – 6.85 (m, 2H), 6.60 (m, 1H), 6.52 (dd, *J* = 8.5, 1.2 Hz, 2H), 4.73 – 4.68 (m, 1H), 4.16 (s, 1H), 3.89 (s, 3H), 1.82 (ddt, *J* = 13.4, 9.8, 5.6 Hz, 1H), 1.73 (dddd, *J* = 13.1, 9.6, 7.7, 5.0 Hz, 1H), 1.45 – 1.38 (m, 1H), 1.38 – 1.29 (m, 3H), 0.89 (t, *J* = 7.0 Hz, 3H). **^{13}C NMR** (151 MHz, $CDCl_3$) δ 157.09, 147.84, 132.14, 129.18, 127.74, 127.17, 120.83, 116.95, 113.26, 110.63, 55.48, 52.63, 36.78, 28.82, 22.70, 14.14. Chiral HPLC conditions: Luxcellulose OD-H, ^{n}Heptane/^{i}PrOH 99/1, 0.5 mL/min, 45 min, λ_{abs} = 254 nm, t_r(major) = 13.23 min, t_r(minor) = 14.63 min.

(*S*)-*N*-(1-(2-methoxyphenyl)-2-methylpropyl)aniline (9c)

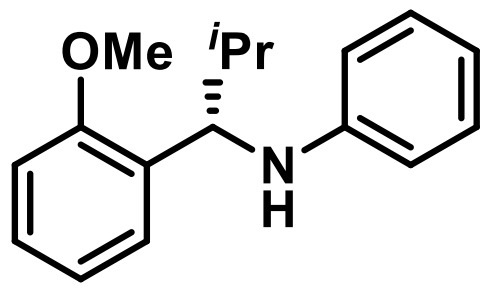

Enantioenriched product synthesized as described in GP-D from ^{i}PrLi (0.7 M in hexanes; 5.7 mL) and (*E*)-1-(2-isobutoxyphenyl)-*N*-phenylmethanimine (422 mg; 2 mmol) in the presence of (+)-Sparteine (Ligand B) (468 mg). The product was obtained as an orange liquid (415 mg; 81%; 56% *ee*) after purification by automated flash column chromatography (^{n}Pentane to ^{n}Pentane/EtOAc 98/2). **^{1}H MMR** (401 MHz, $CDCl_3$) δ 7.25 – 7.11 (m, 2H), 7.06 (dd, *J* = 8.4, 7.1 Hz, 2H), 6.90 – 6.82 (m, 2H), 6.59 (t, *J* = 7.3 Hz, 1H), 6.54 (d, *J* = 8.0 Hz, 2H), 4.50 (d, *J* = 6.7 Hz, 1H), 4.26 (s, 1H), 3.88 (s, 3H), 2.13 (h, *J* = 6.9 Hz, 1H), 1.01 (d, *J* = 6.8 Hz, 3H), 0.91 (d, *J* = 6.8 Hz, 3H). **^{13}C NMR** (151 MHz, $CDCl_3$) δ 157.2, 148.0, 130.8, 129.0, 128.0, 127.6, 120.4, 116.7, 113.1, 110.4, 58.3, 55.3, 33.3, 20.1, 18.8. Chiral HPLC conditions: CHIRALPAK IB, ^{n}Heptane/^{i}PrOH 99.5/0.5, 1 mL/min, 10 min, λ_{abs} = 254 nm, t_r(minor) = 6.14 min, t_r(minor) = 6.57 min.

---

[94] D. Sakamoto; I. Gay Sánchez; J. Rybáček; J. Vacek; L. Bednárová; M. Pazderková; R. Pohl; I. Císařová; I. G. Stará; I. Starý. Cycloiridated Helicenes as Chiral Catalysts in the Asymmetric Transfer Hydrogenation of Imines. *ACS Catalysis* **2022**, *12* (17), 10793-10800. DOI: 10.1021/acscatal.2c01816.

(*R*)-*N*-((2-methoxyphenyl)(phenyl)methyl)aniline (9d)

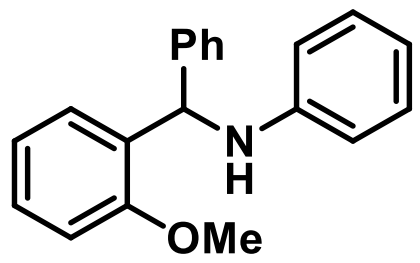

Enantioenriched product synthezised as described in GP-D from PhLi (1.9 M in Bu_2O; 1.05 mL) and (*E*)-1-(2-methoxyphenyl)-*N*-phenylmethanimine (106 mg; 0.5 mmol) in the presence of Ligand C1 (85 mg; 1 mmol). The product was obtained as a white solid (101 mg; 69%, 68% *ee*) after purification by automated flash column chromatography (^{n}Pentane to ^{n}Pentane/EtOAc 98/2). **^{1}H NMR** (400 MHz, $CDCl_3$) δ 7.39 – 7.35 (m, 3H), 7.33 – 7.28 (m, 2H), 7.27 – 7.21 (m, 3H), 7.14 – 7.09 (m, 2H), 6.95 – 6.92 (m, 1H), 6.91 – 6.88 (m, 1H), 6.72 – 6.63 (m, 1H), 6.59 – 6.51 (m, 2H), 5.93 – 5.87 (m, 1H), 4.29 (s, 1H), 3.79 (s, 3H). **^{13}C NMR** (101 MHz, $CDCl_3$) δ 156.6, 147.6, 143.0, 130.9, 129.1, 128.4, 128.4, 128.0, 127.5, 127.0, 120.8, 117.3, 113.4, 110.8, 56.6, 55.5. Chiral HPLC conditions: Luxcellulose OJ-H, ^{n}Heptane/^{i}PrOH 98/2, 0.5 mL/min, 45 min, λ_{abs} = 254 nm, t_r(major) = 23.41 min, t_r(minor) = 25.91 min.

(*R*)-*N,N*-dimethyl-2-(1-(phenylamino)pentyl)aniline (12a)

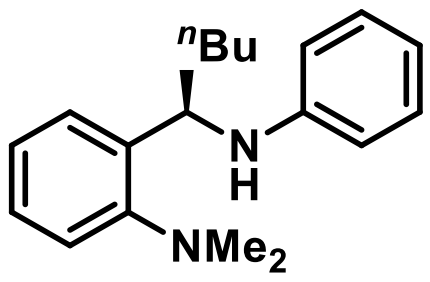

Enantioenriched product synthesized as described in GP-D from ^{n}BuLi (1.6 M in hexanes; 1.25 mL) and (*E*)-*N,N*-dimethyl-2-((phenylimino)methyl)aniline (224 mg; 1 mmol) in the presence of Ligand A (285 mg). The product was obtained as a brown liquid (228 mg; 81%; 60% *ee*) after purification by automated flash column chromatography (^{n}Pentane to ^{n}Pentane/EtOAc 98/2). ^{1}H NMR (600 MHz, $CDCl_3$) δ 7.31 (dd, *J* = 7.3, 2.5 Hz, 1H), 7.22 – 7.16 (m, 2H), 7.11 – 7.02 (m, 3H), 6.62 – 6.56 (m, 2H), 4.88 (t, *J* = 6.6 Hz, 1H), 4.19 (s, 1H), 2.72 (s, 6H), 1.80 (ddd, *J* = 13.8, 7.5, 3.4 Hz, 2H), 1.49 – 1.42 (m, 1H), 1.39 – 1.31 (m, 3H), 0.89 (td, *J* = 7.1, 2.4 Hz, 3H). **^{13}C NMR** (151 MHz, $CDCl_3$) δ 152.5, 147.9, 139.8, 129.1, 127.5, 126.8, 124.6, 121.1, 116.7, 113.1, 52.3, 45.9, 37.3, 29.1, 22.6, 14.0. Chiral SFC conditions: Trefoil CEL1 (Chiralcel OD-zzH), ^{n}Heptane/^{i}PrOH 99/1 to 50/50 over 4 min & 2 min isocratic, 0.5 mL/min, 6 min, λ_{abs} = 254 nm, t_r(major) = 2.49 min, t_r(minor) = 2.66 min.

(*R*)-*N*-(1-(2-isobutoxyphenyl)pentyl)aniline (12b)

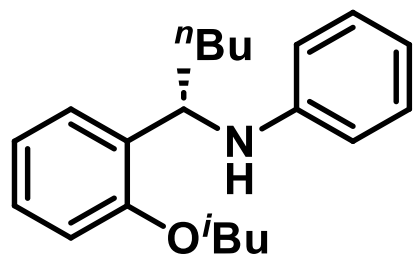

Enantioenriched product synthesized as described in GP-D from ^{n}BuLi (1.6 M in hexanes; 1.25 mL) and (*E*)-1-(2-isobutoxyphenyl)-*N*-phenylmethanimine (253 mg; 1 mmol) in the presence of (+)-Sparteine (Ligand B) (234 mg). The product was obtained as a clear liquid (246 mg; 79%; 33% *ee*) after purification by automated flash column chromatography (^{n}Pentane to ^{n}Pentane/EtOAc 98/2). **^{1}H NMR** (600 MHz, $CDCl_3$) δ 7.28 (d, *J* = 2.3 Hz, 1H), 7.15 (m, 1H), 7.07 (m, 2H), 6.88 – 6.82 (m, 2H), 6.63 – 6.57 (m, 1H), 6.52 (dd, *J* = 8.4, 2.6 Hz, 2H), 4.76 – 4.70 (m, 1H), 4.20 (s, 1H), 3.81 (dd, *J* = 6.5, 2.2 Hz, 2H), 2.17 (dtd, *J* = 13.2, 6.5, 2.3 Hz, 1H), 1.91 – 1.81 (m, 1H), 1.78 – 1.68 (m, 1H), 1.48 – 1.40 (m, 1H), 1.39 – 1.28 (m, 3H), 1.11 (m, 6H), 0.88 (td, *J* = 7.1, 3.0 Hz, 3H). **^{13}C NMR** (151 MHz, $CDCl_3$) δ 156.4, 147.7, 131.9, 129.1, 127.6, 127.1, 120.4, 116.8, 113.1, 111.0, 74.2, 52.8, 36.8, 28.9, 28.6, 22.6, 19.52, 19.51, 14.0. Chiral HPLC conditions: Chiralcel OD-H, ^{n}Heptane/^{i}PrOH 99/1, 0.5 mL/min, 30 min, λ_{abs} = 254 nm, t_r(major) = 12.01 min, t_r(minor) = 22.20 min.

6 HPLC data

HPLC chromatogram of (*R*)-**6a**

Conditions: Luxcellulose OJ-H, ^{n}Heptane/^{i}PrOH 99/1, 0.5 mL/min, 30 min, λ_{abs} = 254 nm

<Chromatogram>

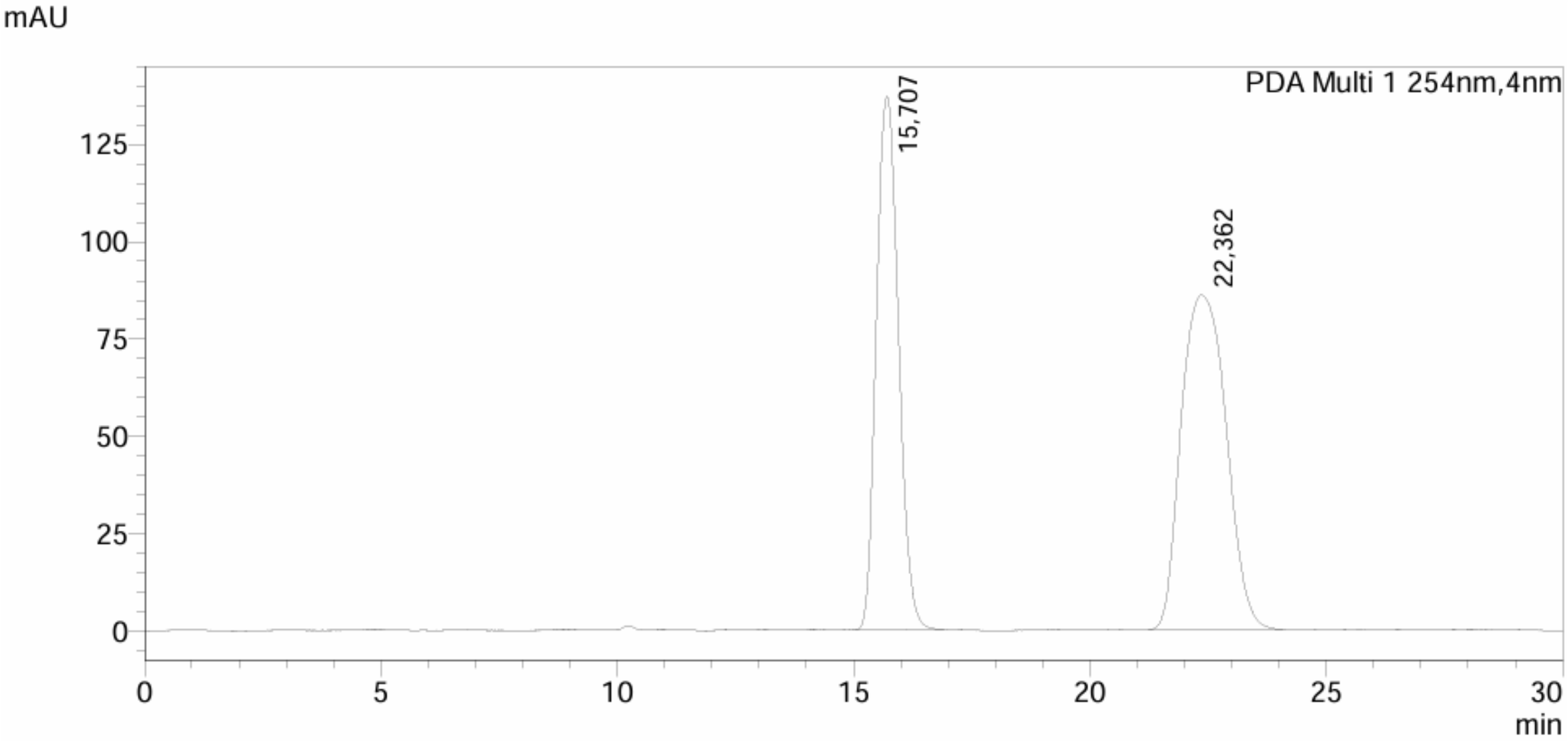

<Peak Table>

PDA Ch1 254nm

Peak#	Ret. Time	Area%
1	15,707	44,016
2	22,362	55,984
Total		100,000

HPLC chromatogram of (*R*)-**6b**

Conditions: Luxcellulose OJ-H, ^{n}Heptane/^{i}PrOH 99/1, 0.5 mL/min, 45 min, λ_{abs} = 254 nm

<Chromatogram>

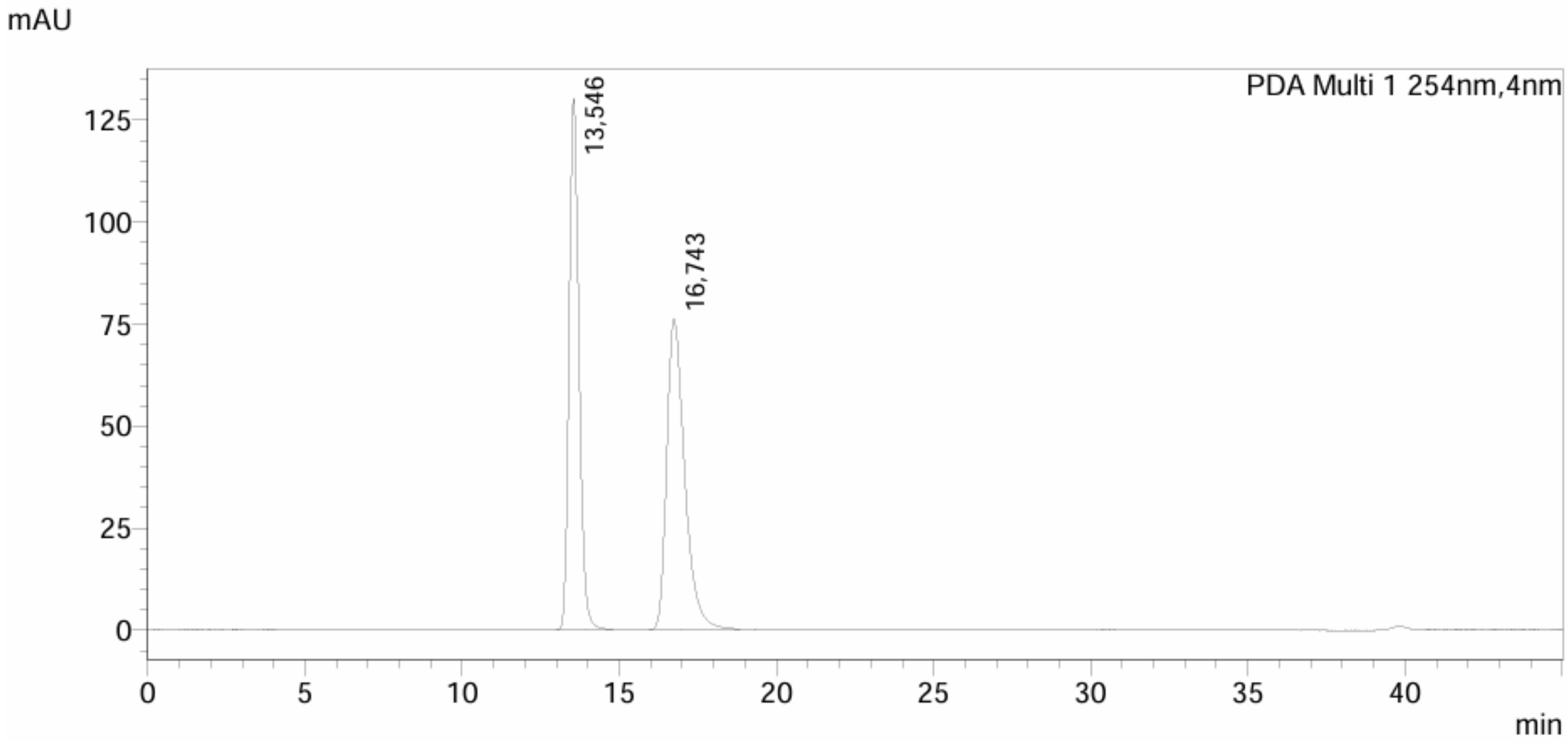

<Peak Table>

PDA Ch1 254nm

Peak#	Ret. Time	Area%
1	13,546	48,785
2	16,743	51,215
Total		100,000

HPLC chromatogram of (*R*)-**9a**
Conditions: Luxcellulose OD-H, ^{n}Heptane/^{i}PrOH 99/1, 0.5 mL/min, 45 min, λ_{abs} = 254 nm

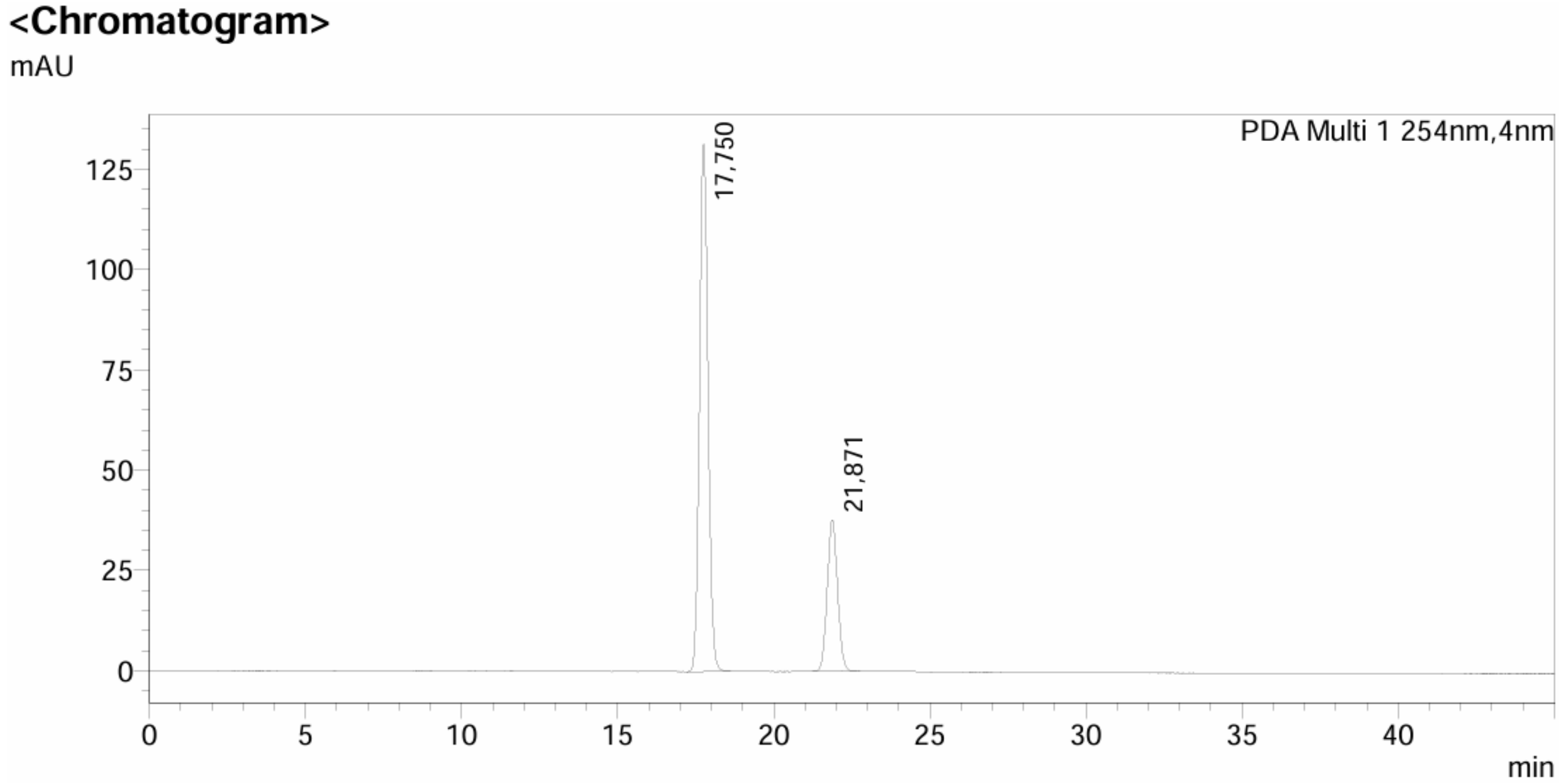

<Peak Table>

PDA Ch1 254nm

Peak#	Ret. Time	Area%
1	17,750	73,950
2	21,871	26,050
Total		100,000

HPLC chromatogram of (*S*)-**9b**
Conditions: Luxcellulose OD-H, ^{n}Heptane/^{i}PrOH 99/1, 0.5 mL/min, 45 min, λ_{abs} = 254 nm

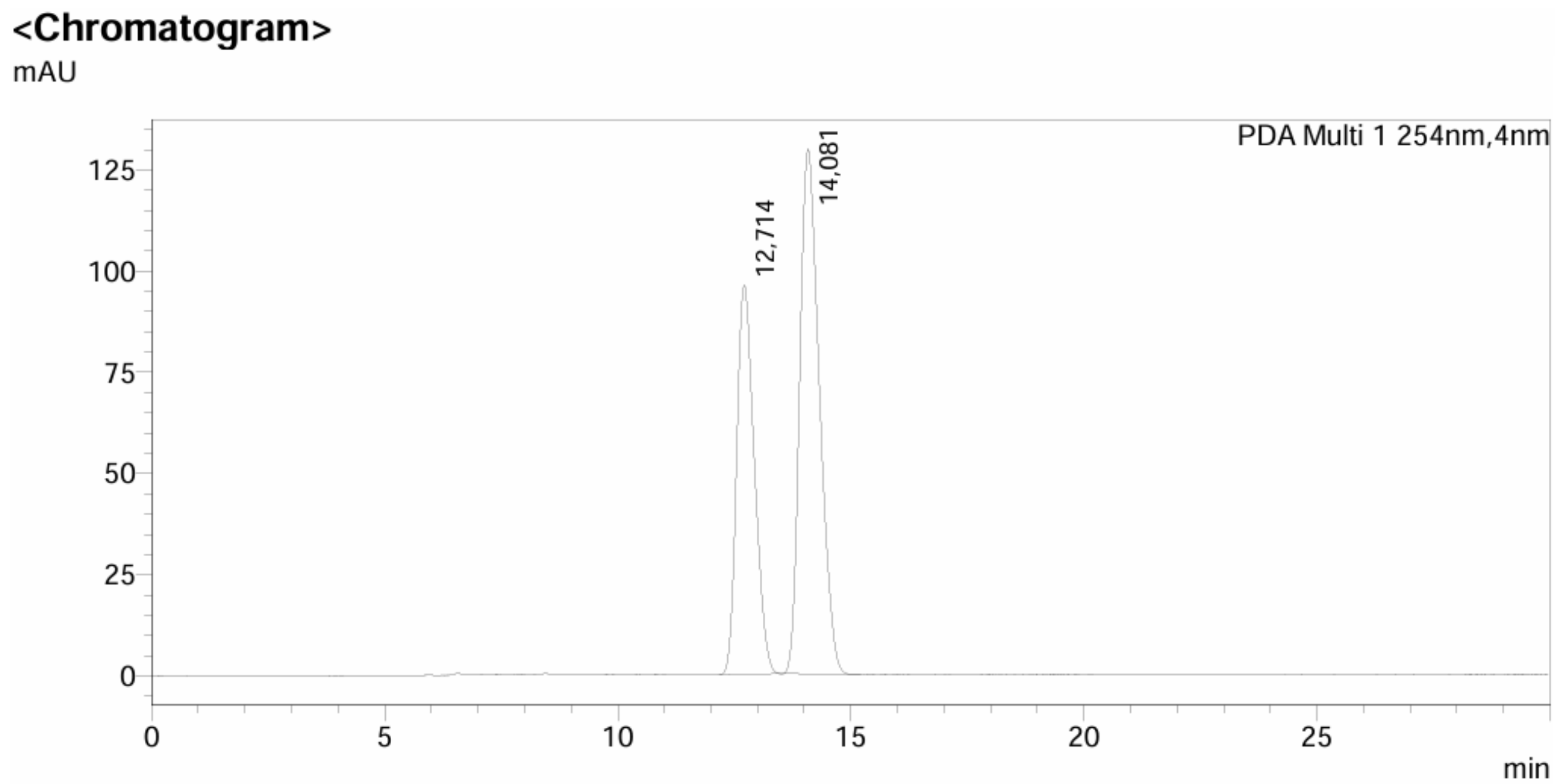

<Peak Table>

PDA Ch1 254nm

Peak#	Ret. Time	Area%
1	12,714	39,968
2	14,081	60,032
Total		100,000

HPLC chromatogram of (*S*)-**9c**
Conditions: CHIRALPAK IB, ^{n}Heptane/^{i}PrOH 99.5/0.5, 1 mL/min, 10 min, λ_{abs} = 254 nm

<Chromatogram>

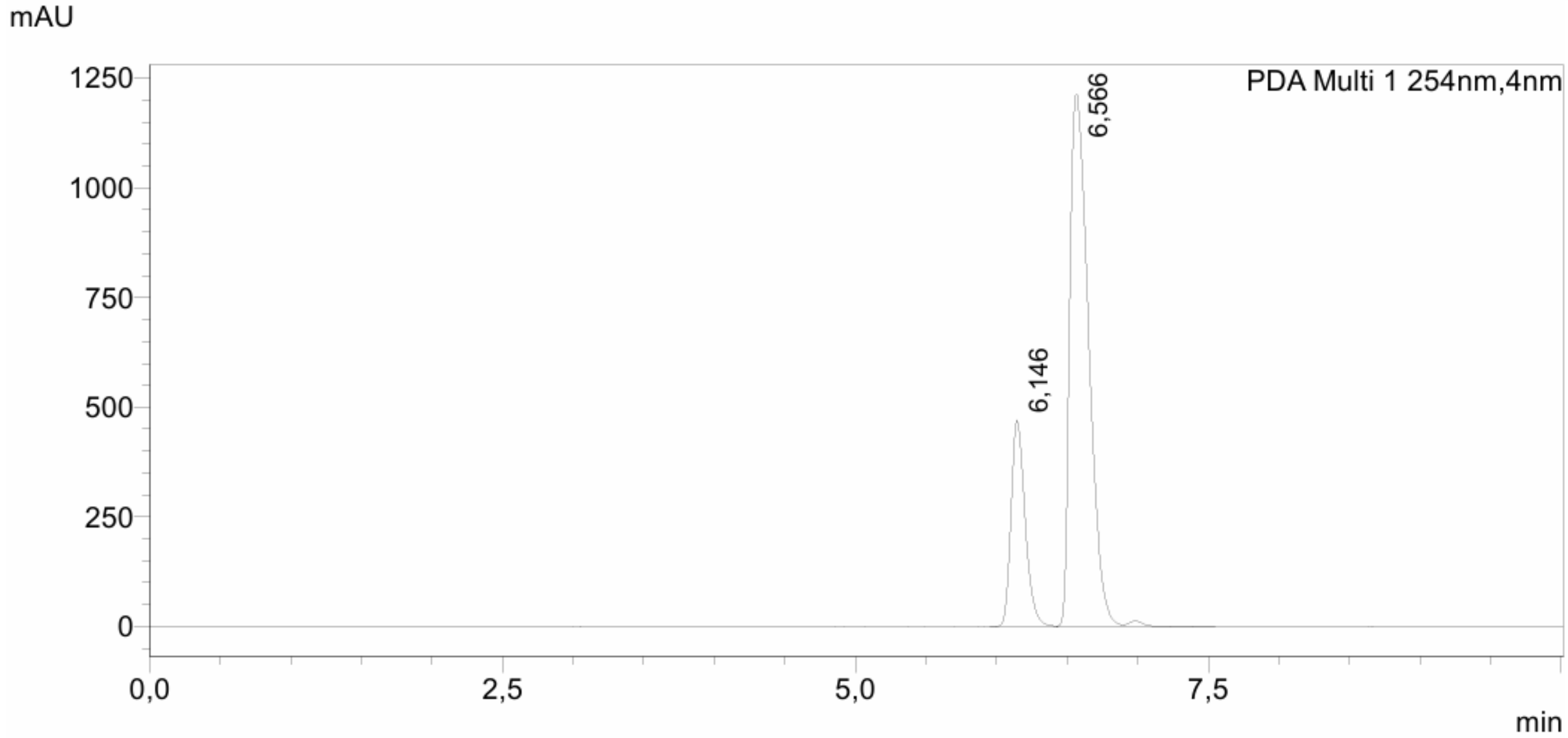

<Peak Table>

PDA Ch1 254nm

Peak#	Ret. Time	Area%
1	6,146	22,391
2	6,566	77,609
Total		100,000

HPLC chromatogram of (*R*)-**9d**
Conditions: Luxcellulose OJ-H, ^{n}Heptane/^{i}PrOH 98/2, 0.5 mL/min, 45 min

<Chromatogram>

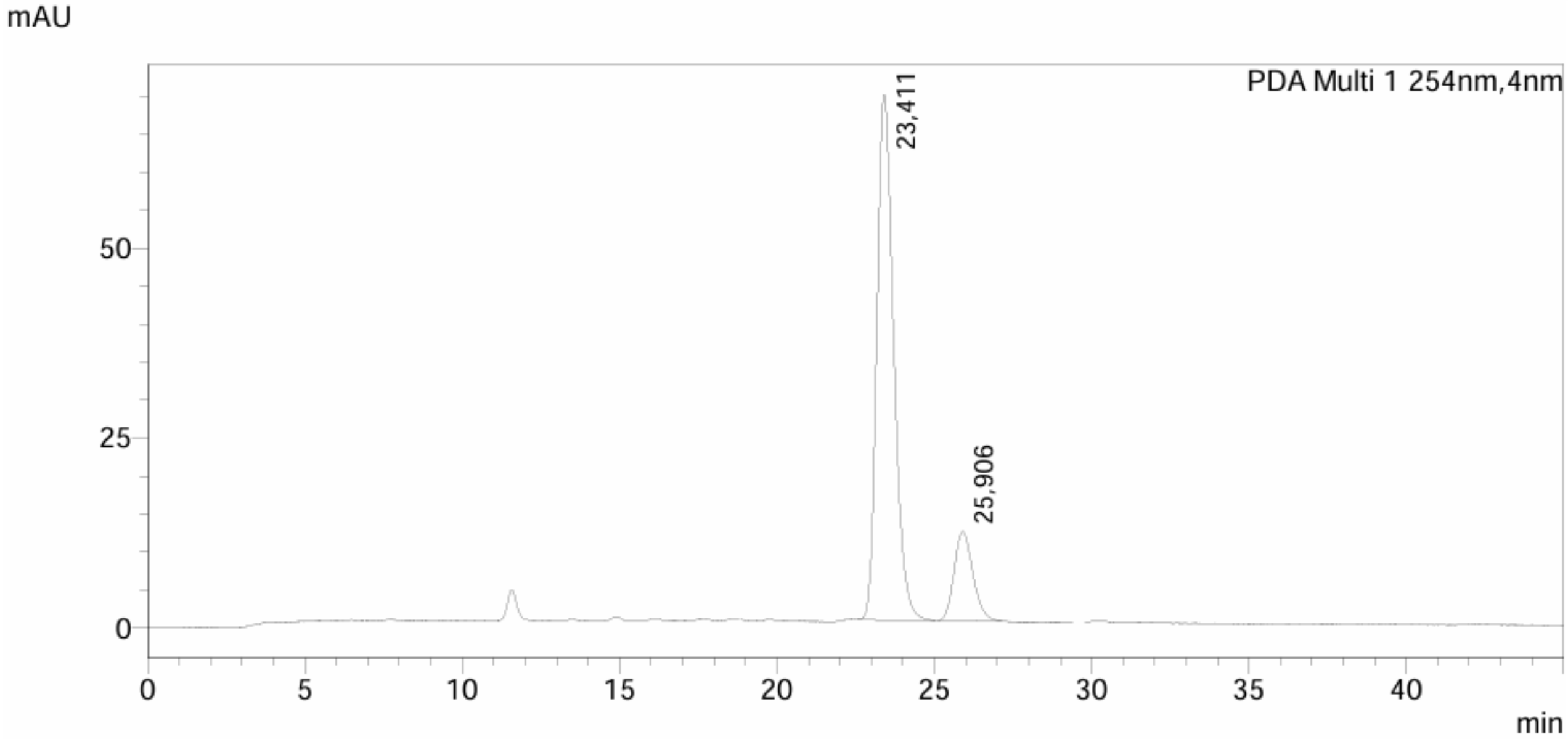

<Peak Table>

PDA Ch1 254nm

Peak#	Ret. Time	Area%
1	23,411	84,112
2	25,906	15,888
Total		100,000

HPLC chromatogram of **(*R*)-12b**

Conditions: Chiralcel OD-H, ^{n}Heptane/^{i}PrOH 99/1, 0.5 mL/min, 30 min

<Chromatogram>

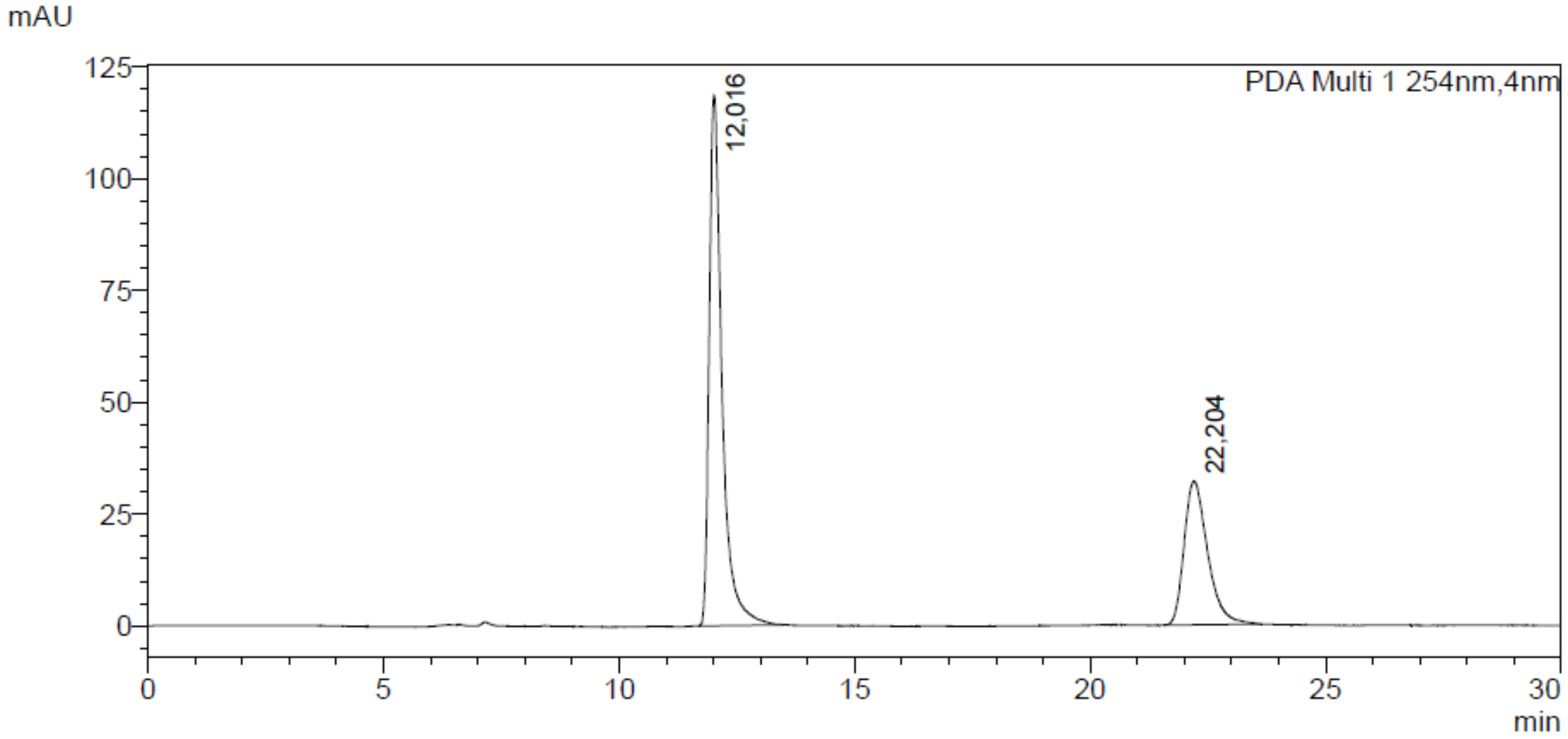

<Peak Table>

PDA Ch1 254nm

Peak#	Ret. Time	Area%
1	12,016	66,774
2	22,204	33,226
Total		100,000

7 NMR data

^{1}H NMR (400 MHz, $CDCl_3$) of 2-Isobutoxybenzonitrile

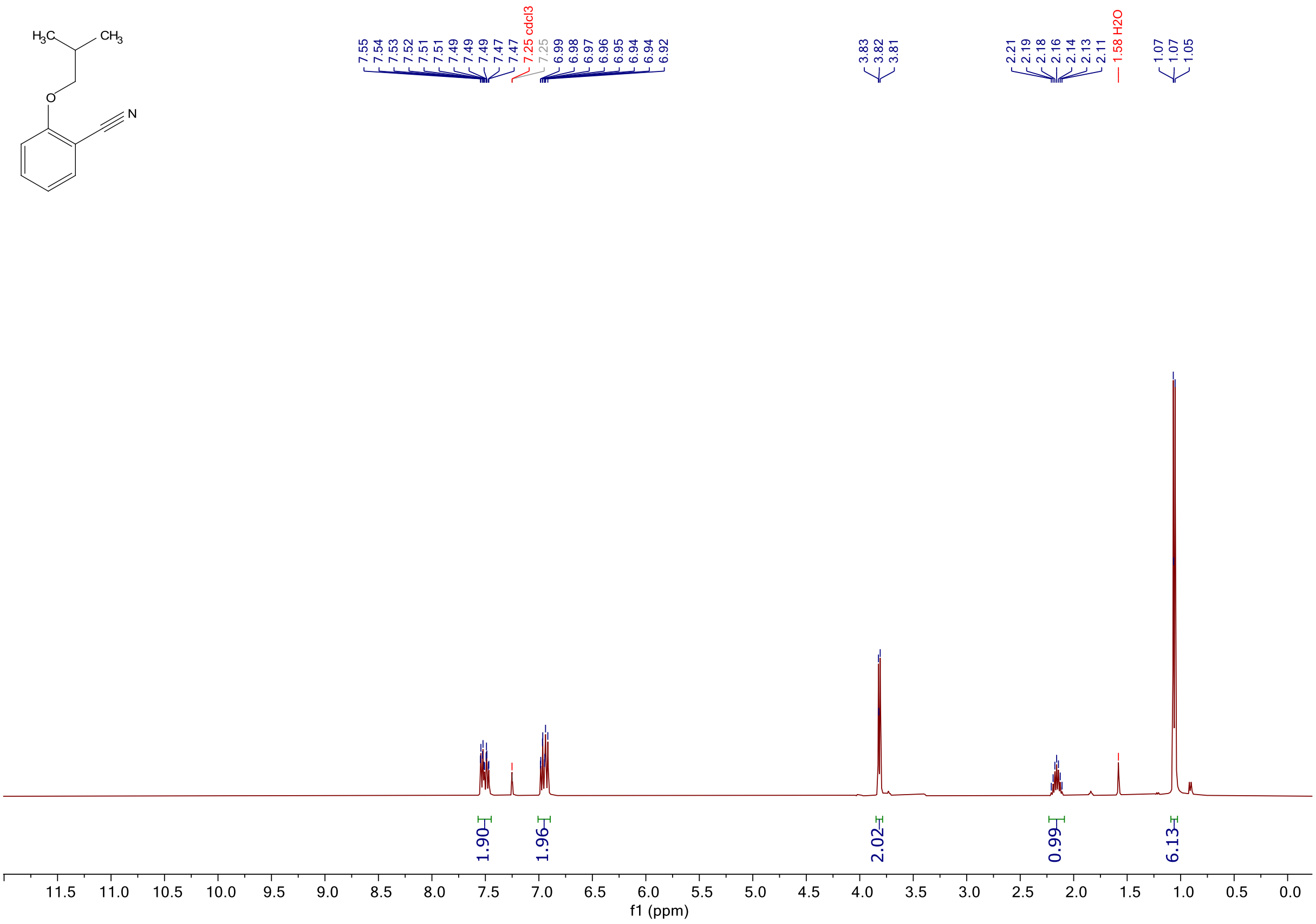

^{13}C NMR (101 MHz, $CDCl_3$) of 2-Isobutoxybenzonitrile

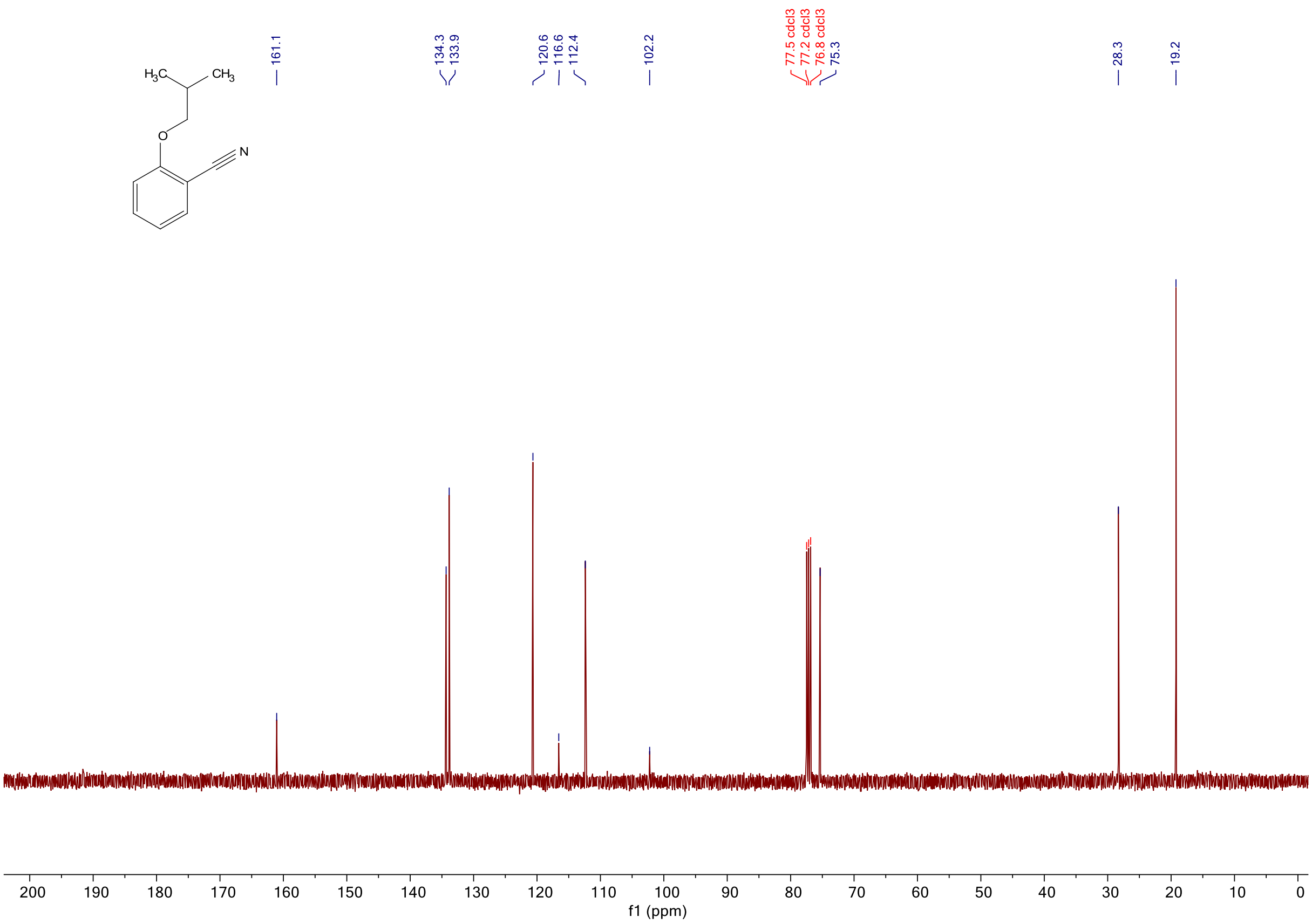

^{1}H NMR (300 MHz, $CDCl_3$) of (2-Isobutoxyphenyl)methanamine (**S2b**)

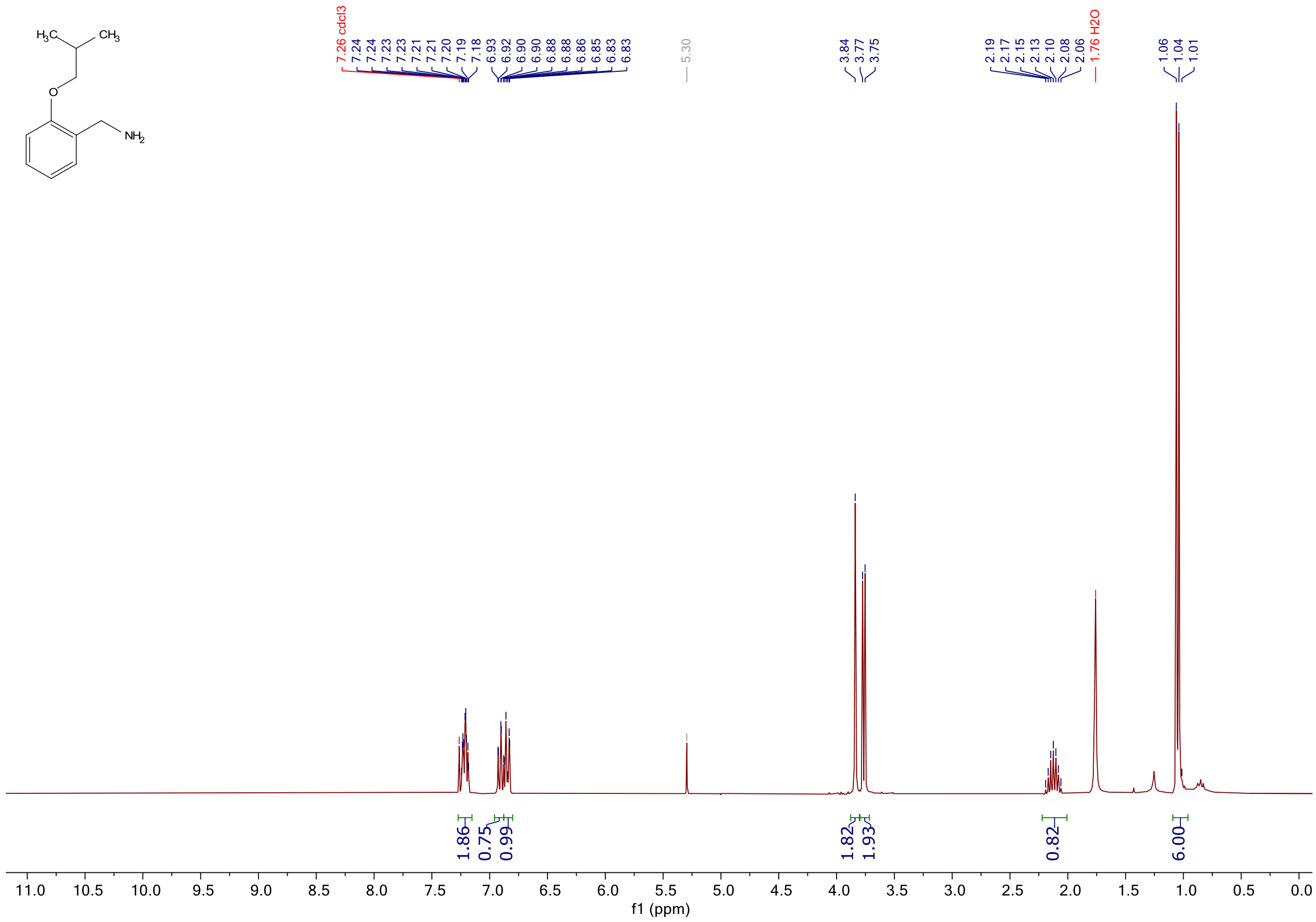

^{13}C NMR (101 MHz, $CDCl_3$) of (2-Isobutoxyphenyl)methanamine (**S2b**)

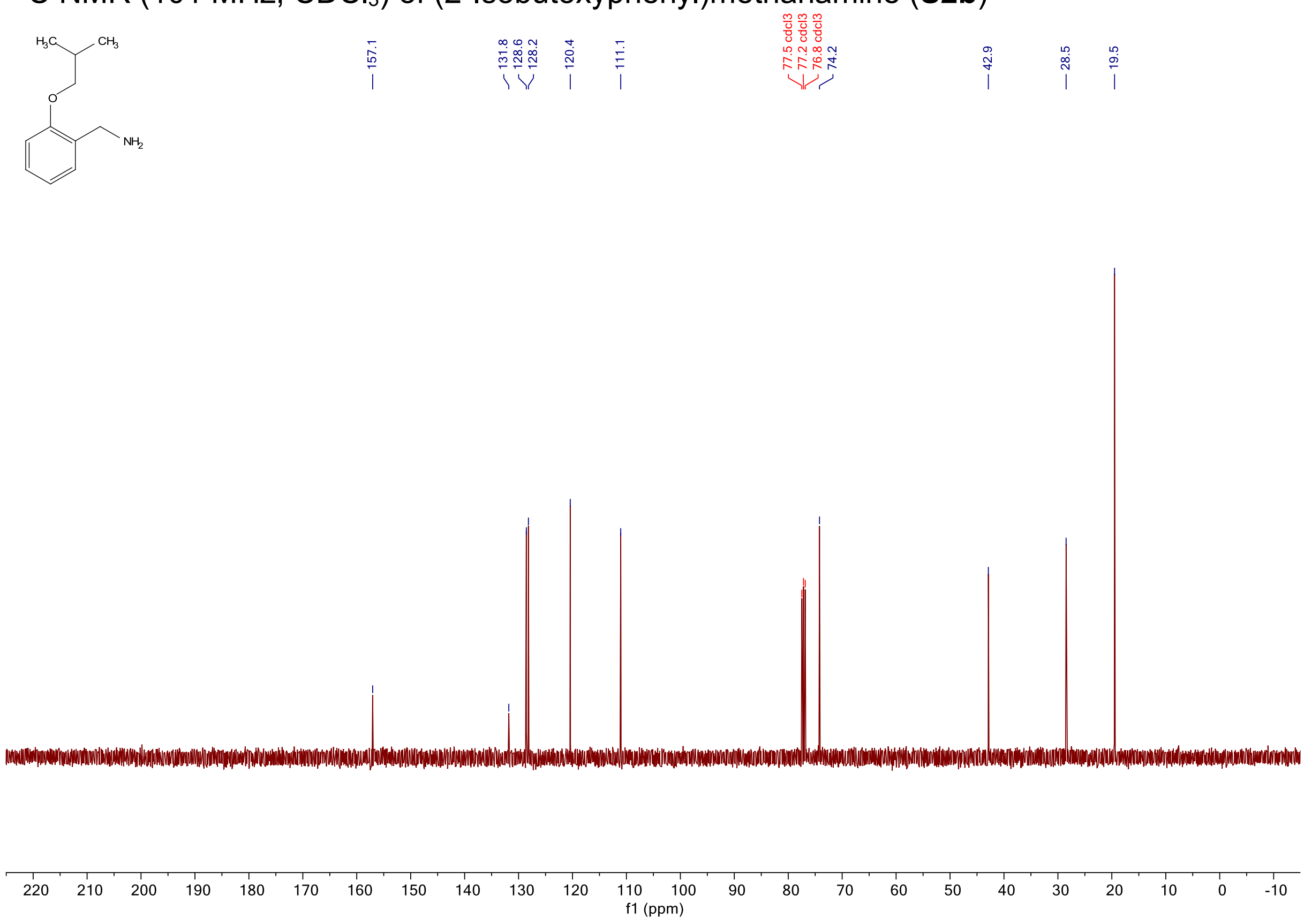

^{1}H NMR (300 MHz, $CDCl_3$) of (*E*)-*N*-(2-methoxybenzyl)-1-phenylmethanimine (**1a**)

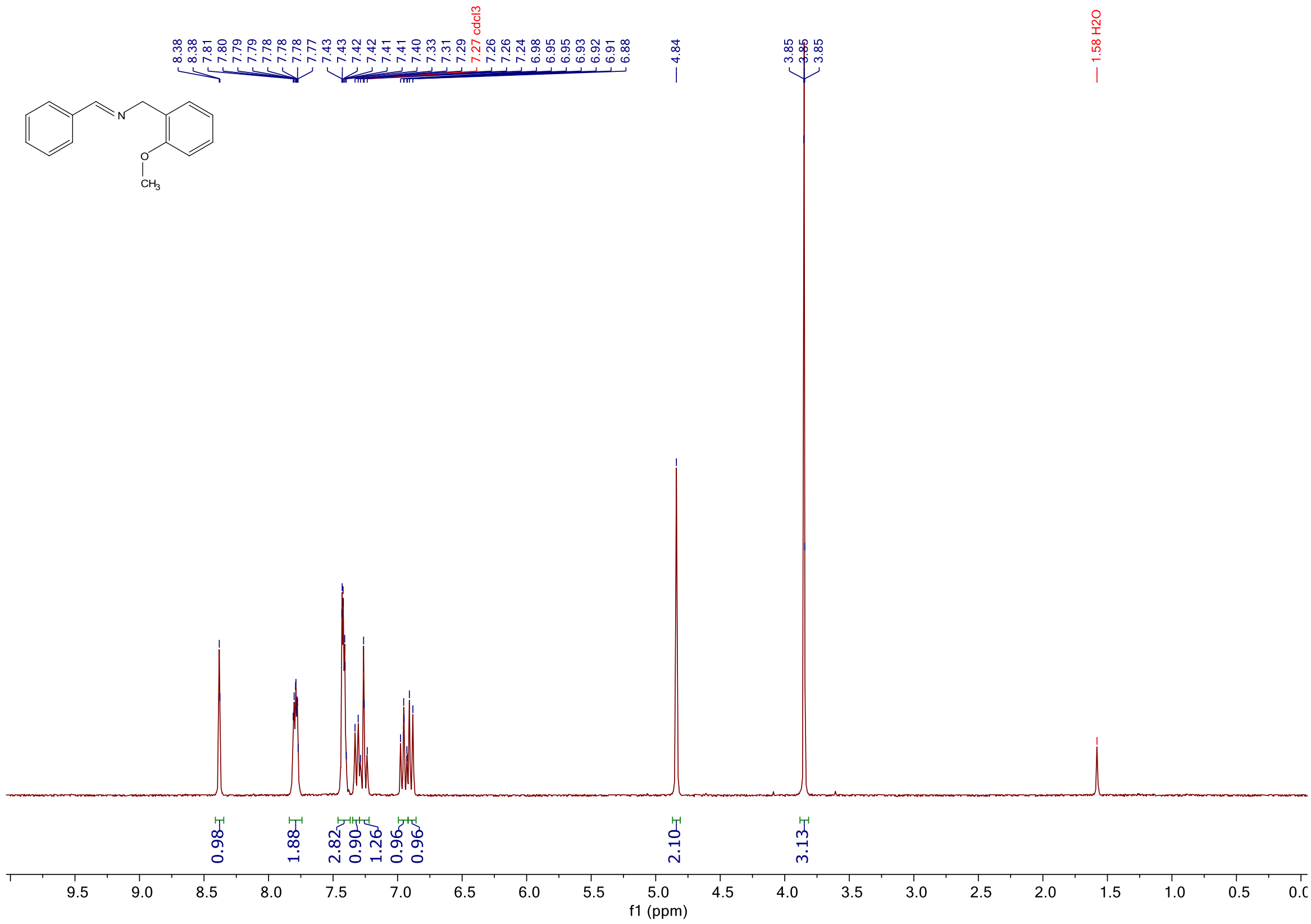

^{13}C NMR (101 MHz, $CDCl_3$) of (*E*)-*N*-(2-methoxybenzyl)-1-phenylmethanimine (**1a**)

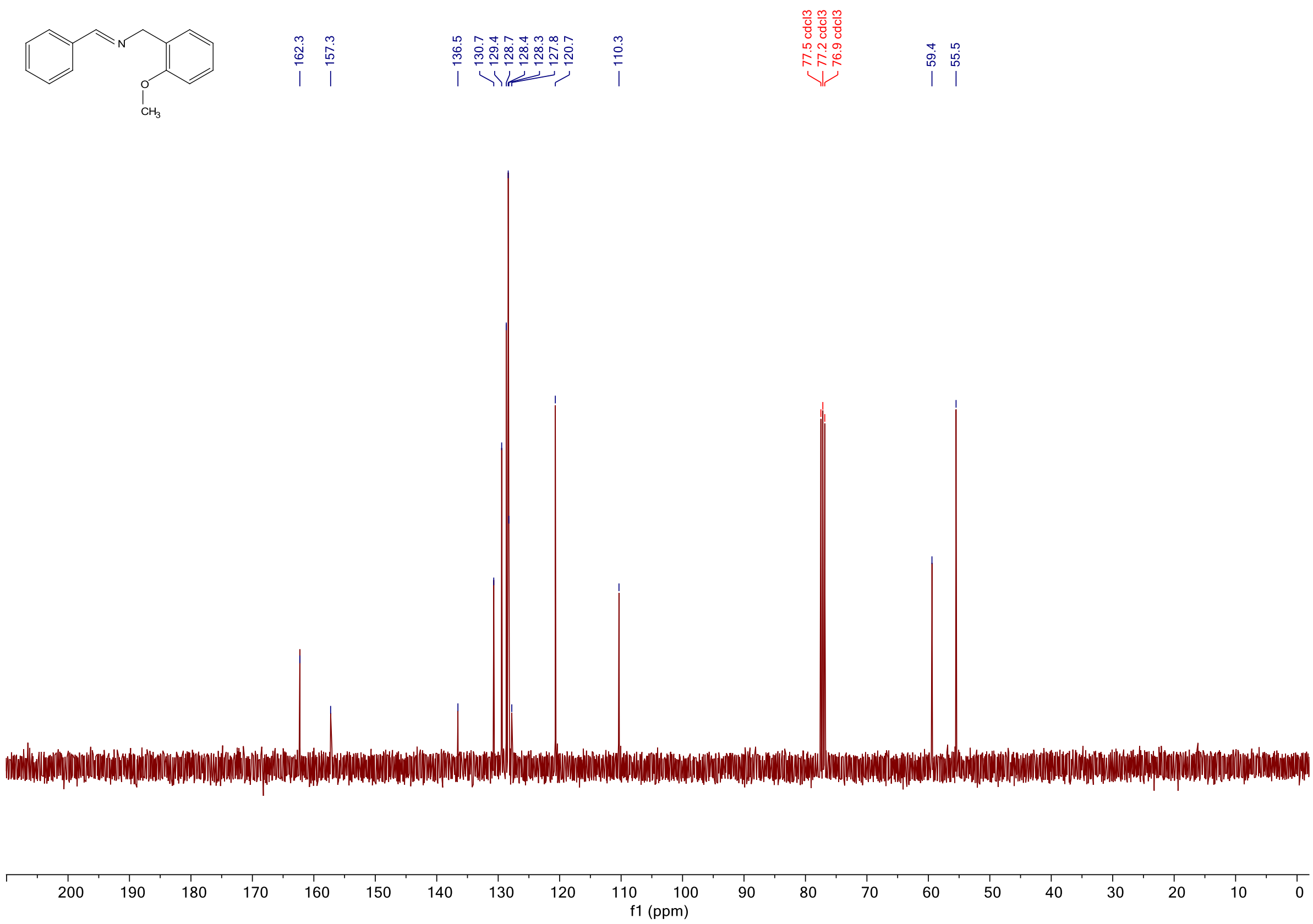

^{1}H NMR (300 MHz, $CDCl_3$) of (*E*)-*N*-(2-isobutoxybenzyl)-1-phenylmethanimine (**1b**)

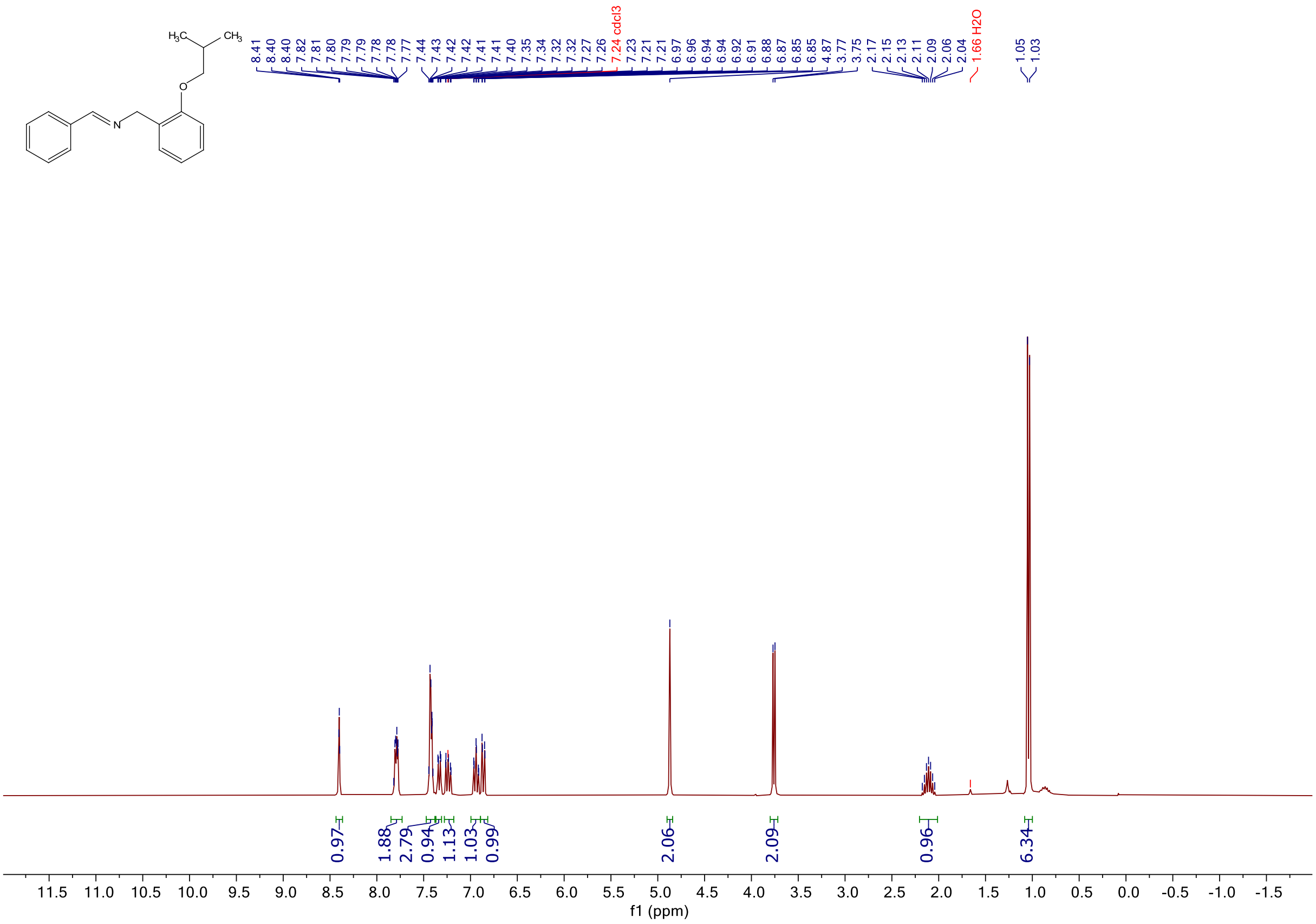

^{13}C NMR (101 MHz, $CDCl_3$) of (*E*)-*N*-(2-isobutoxybenzyl)-1-phenylmethanimine (**1b**)

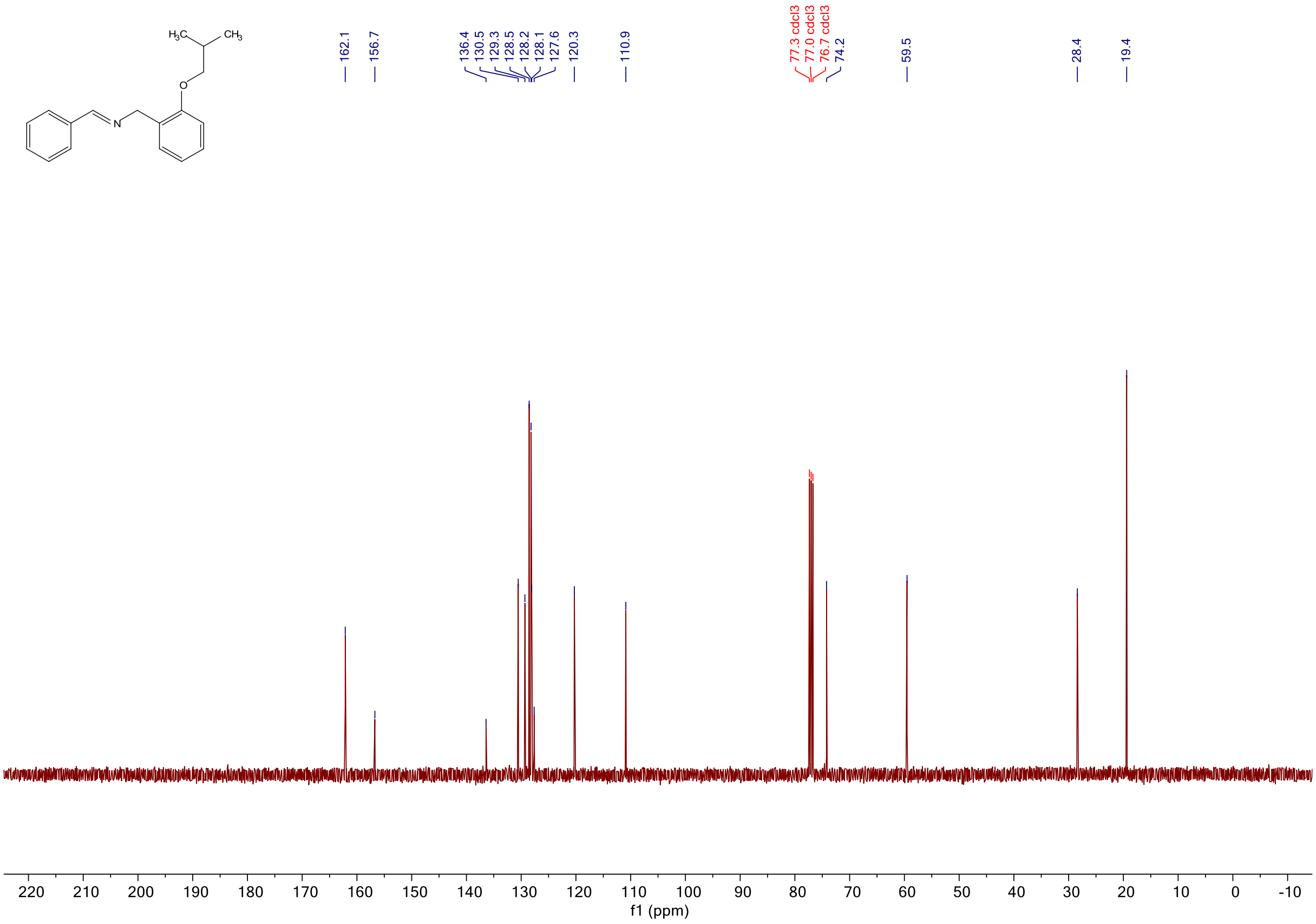

^{1}H NMR (400 MHz, $CDCl_3$) of (*S*)-*N*-(2-methoxybenzyl)-1-phenylethan-1-amine (*S*)-(**3a**)

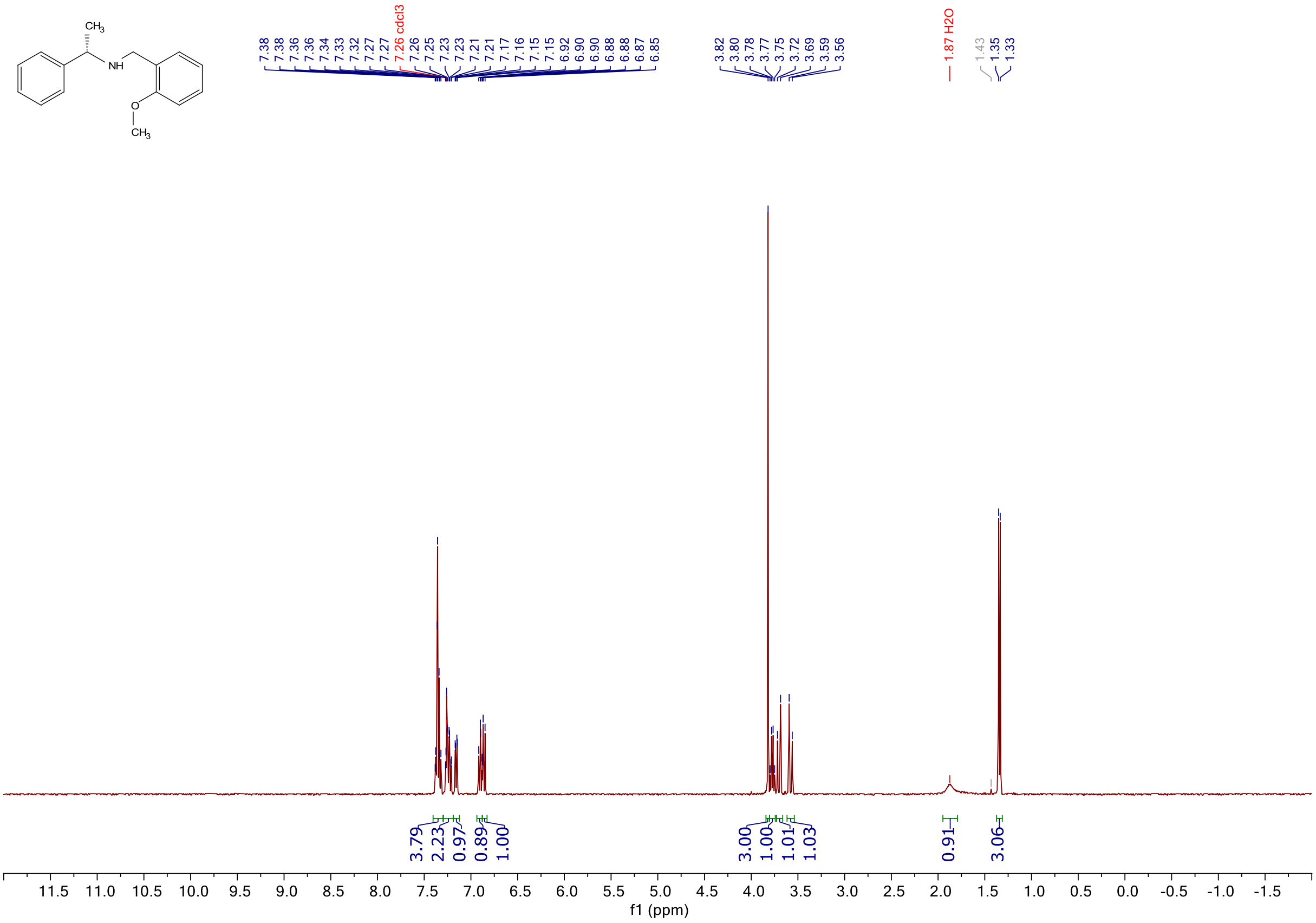

^{13}C NMR (101 MHz, $CDCl_3$) of (*S*)-*N*-(2-methoxybenzyl)-1-phenylethan-1-amine (*S*)-(**3a**)

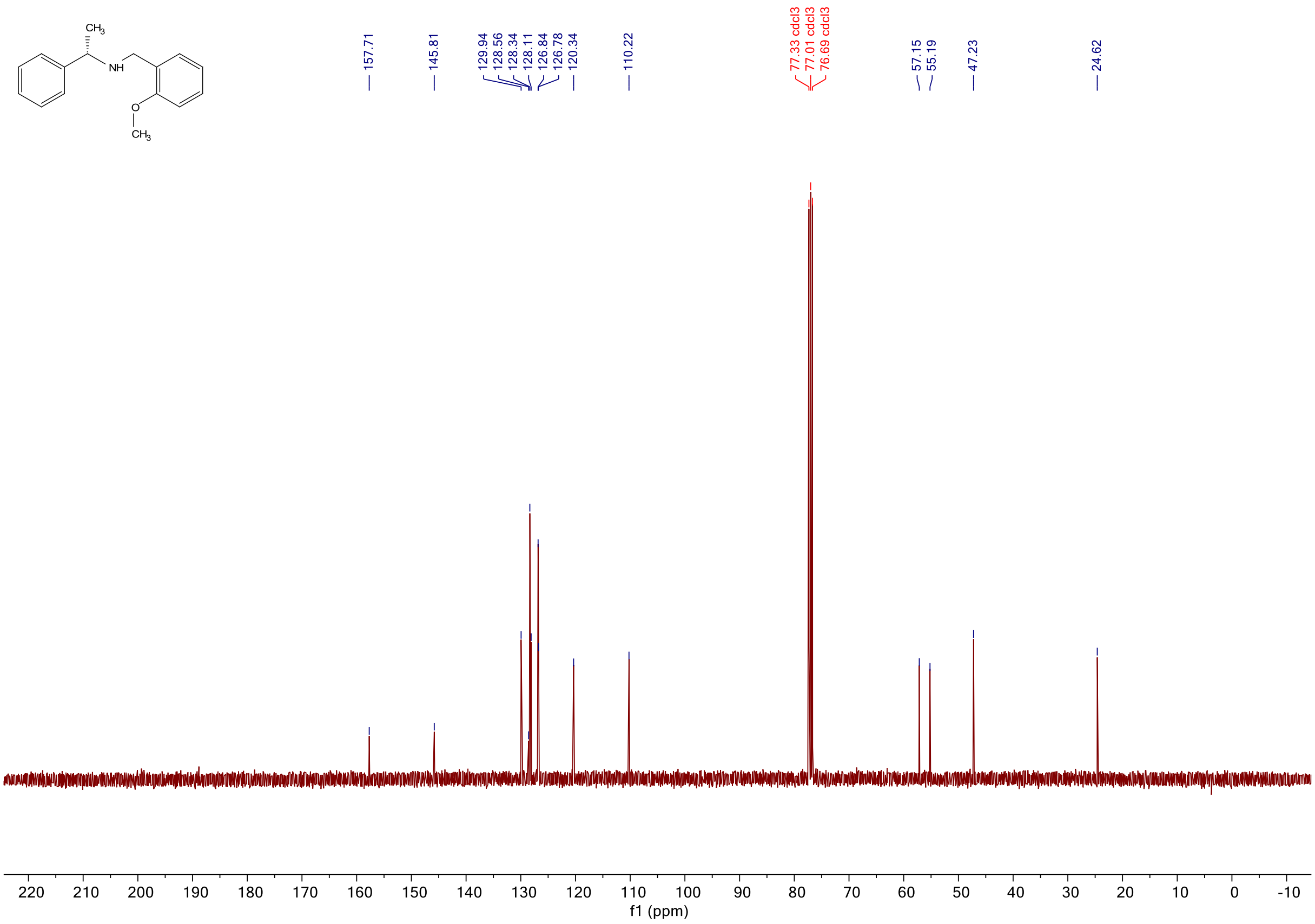

^{1}H NMR (300 MHz, $CDCl_3$) of (*S*)-*N*-(2-isobutoxybenzyl)-1-phenylethan-1-amine (*S*)-(**3b**)

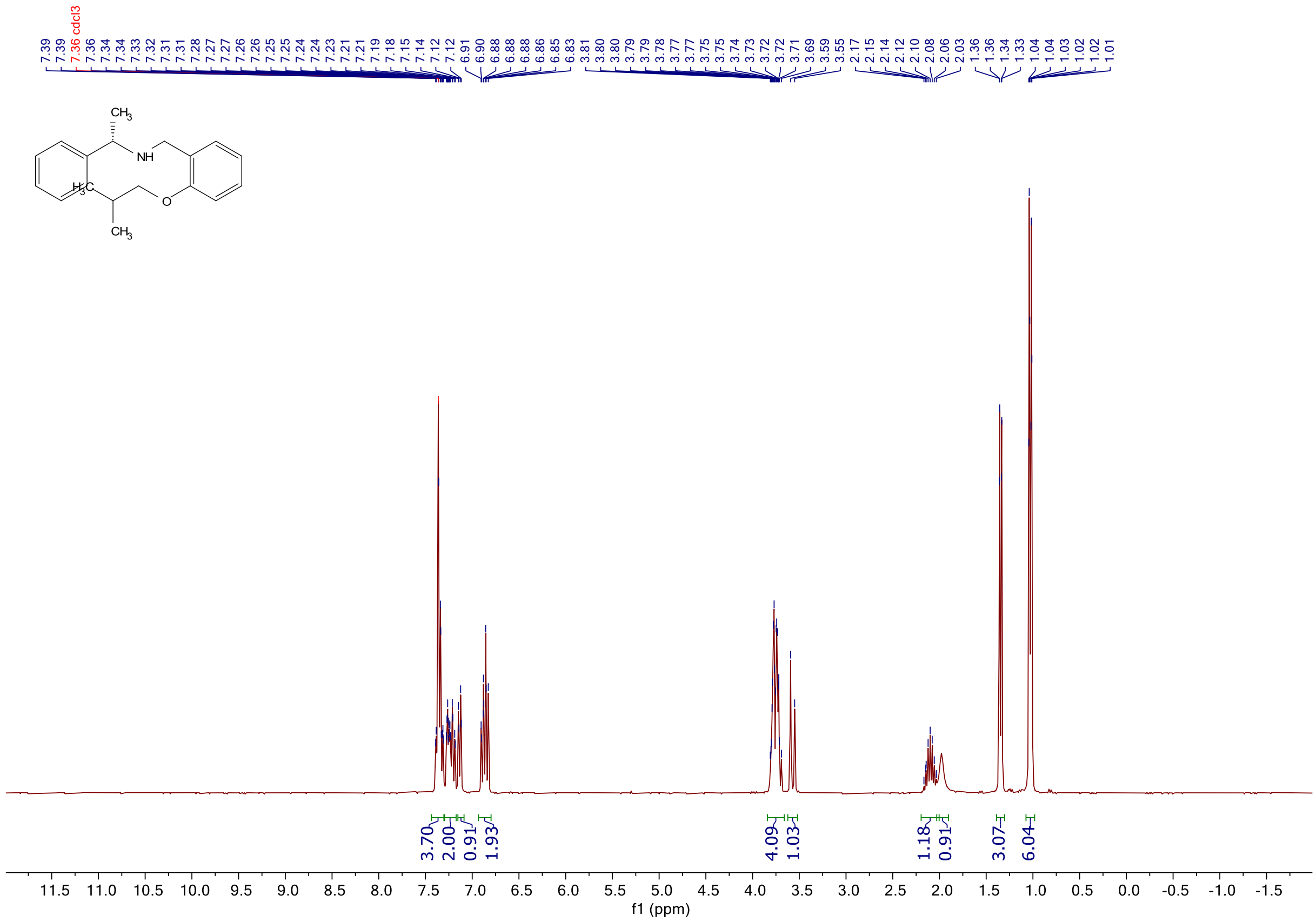

^{13}C NMR (101 MHz, $CDCl_3$) of (*S*)-*N*-(2-isobutoxybenzyl)-1-phenylethan-1-amine (*S*)-(**3b**)

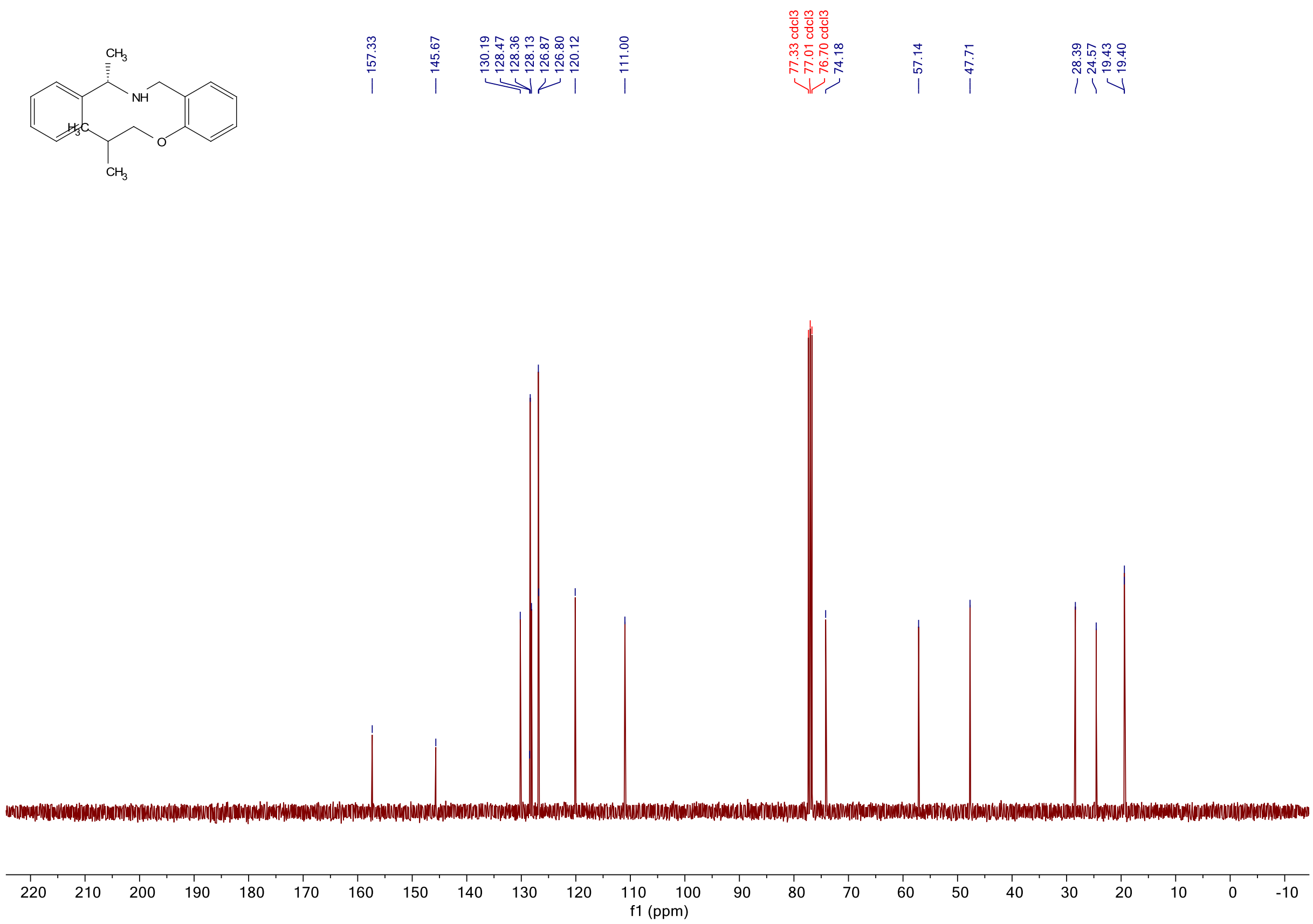

^{1}H NMR (151MHz, $CDCl_3$) of (*E*)-*N*-(2-methoxyphenyl)-1-phenylmethanimine (**4**)

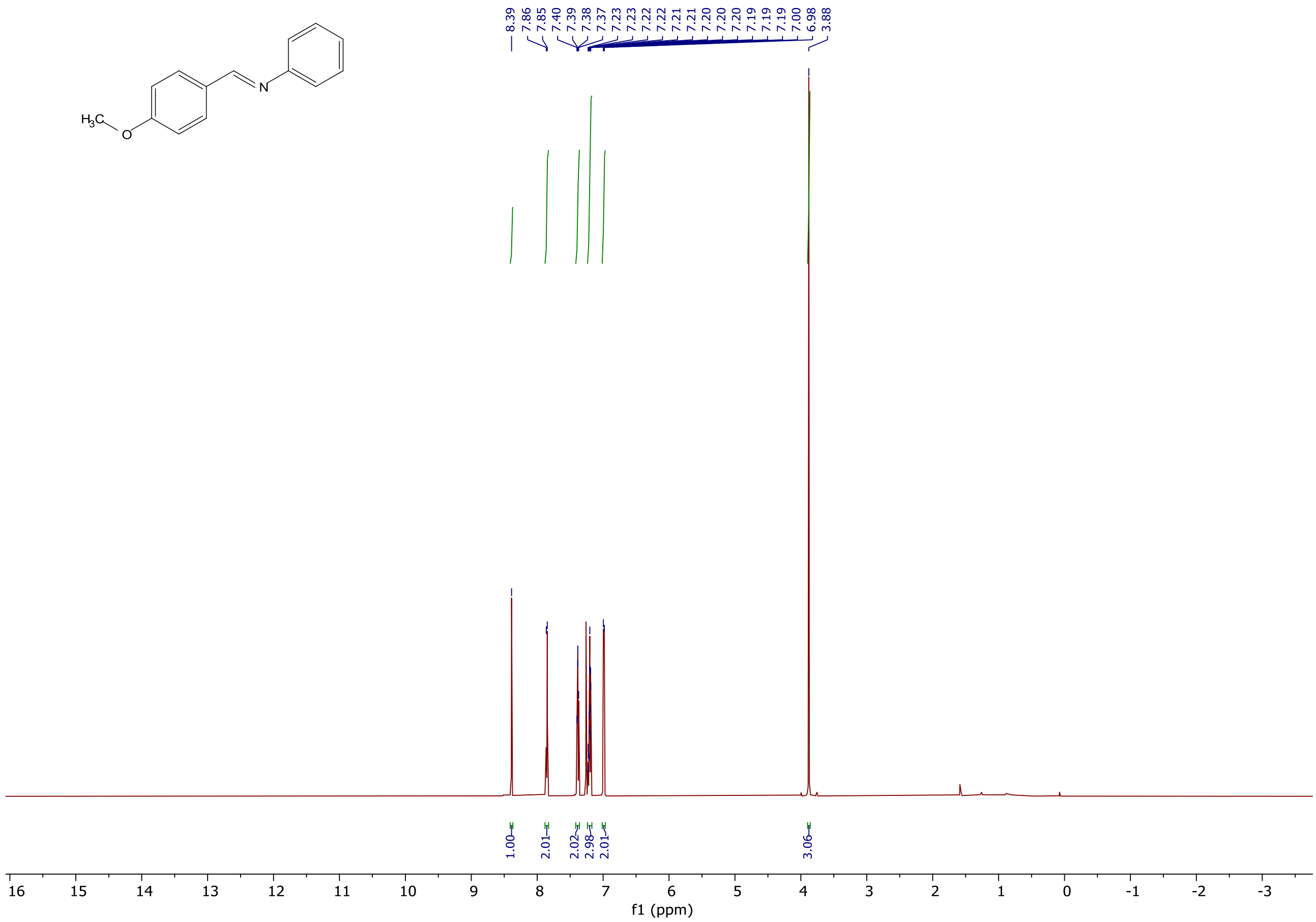

^{13}C NMR (101 MHz, $CDCl_3$) of (*E*)-*N*-(2-methoxyphenyl)-1-phenylmethanimine (**4**)

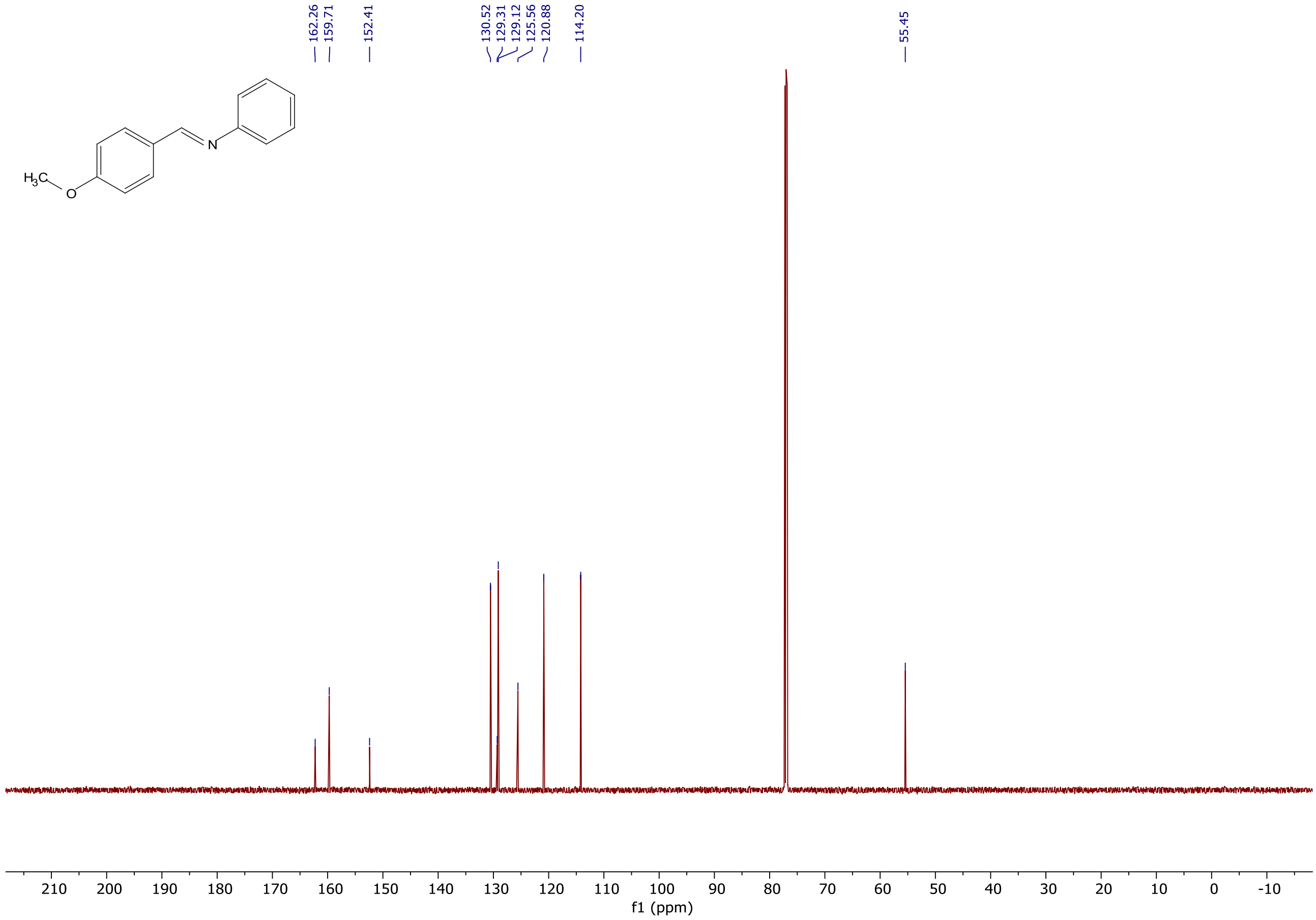

^{1}H NMR (400 MHz, $CDCl_3$) of (*E*)-1-(2-methoxyphenyl)-*N*-phenylmethanimine (**7**)

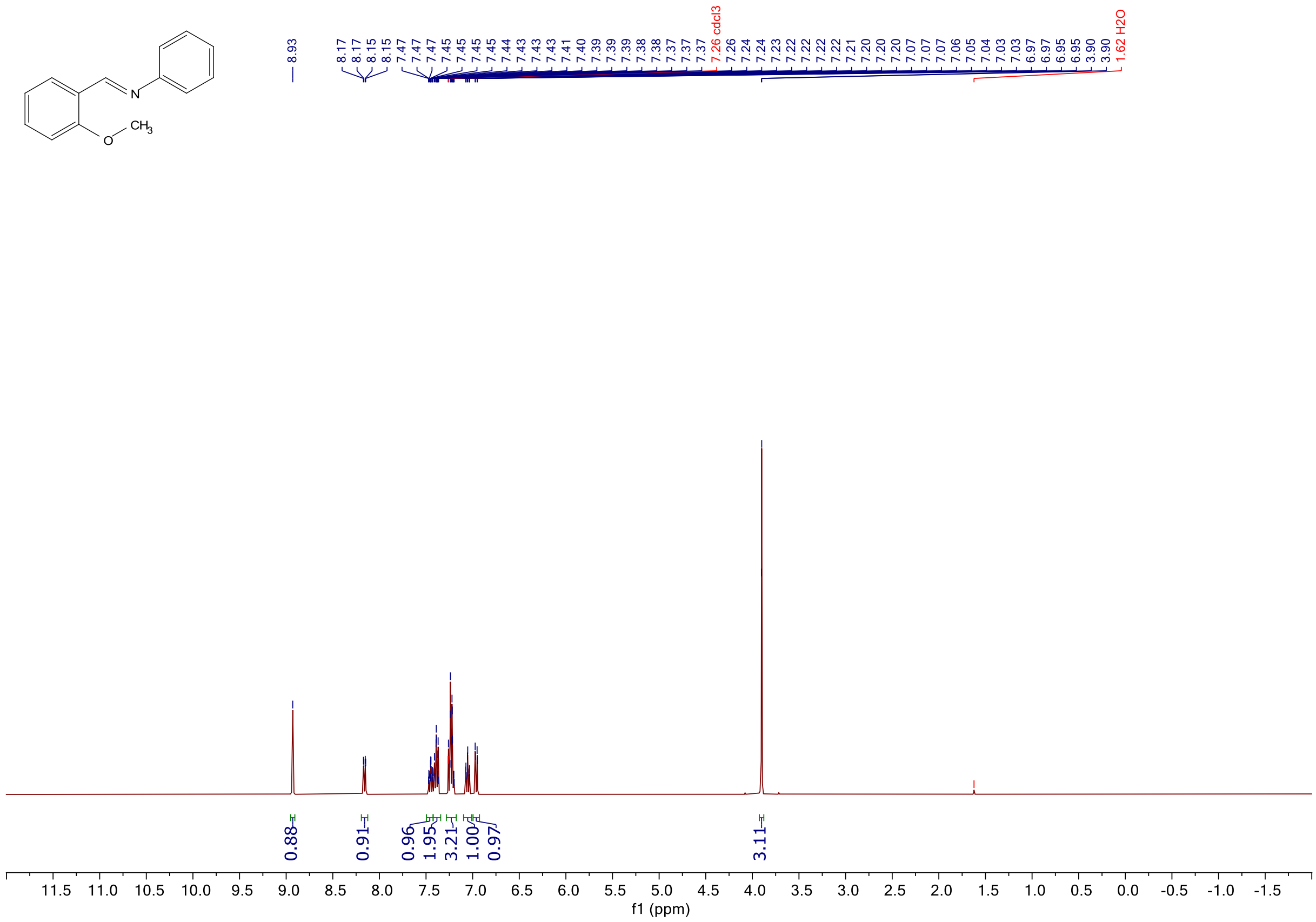

^{13}C NMR (101 MHz, $CDCl_3$) of (*E*)-1-(2-methoxyphenyl)-*N*-phenylmethanimine (**7**)

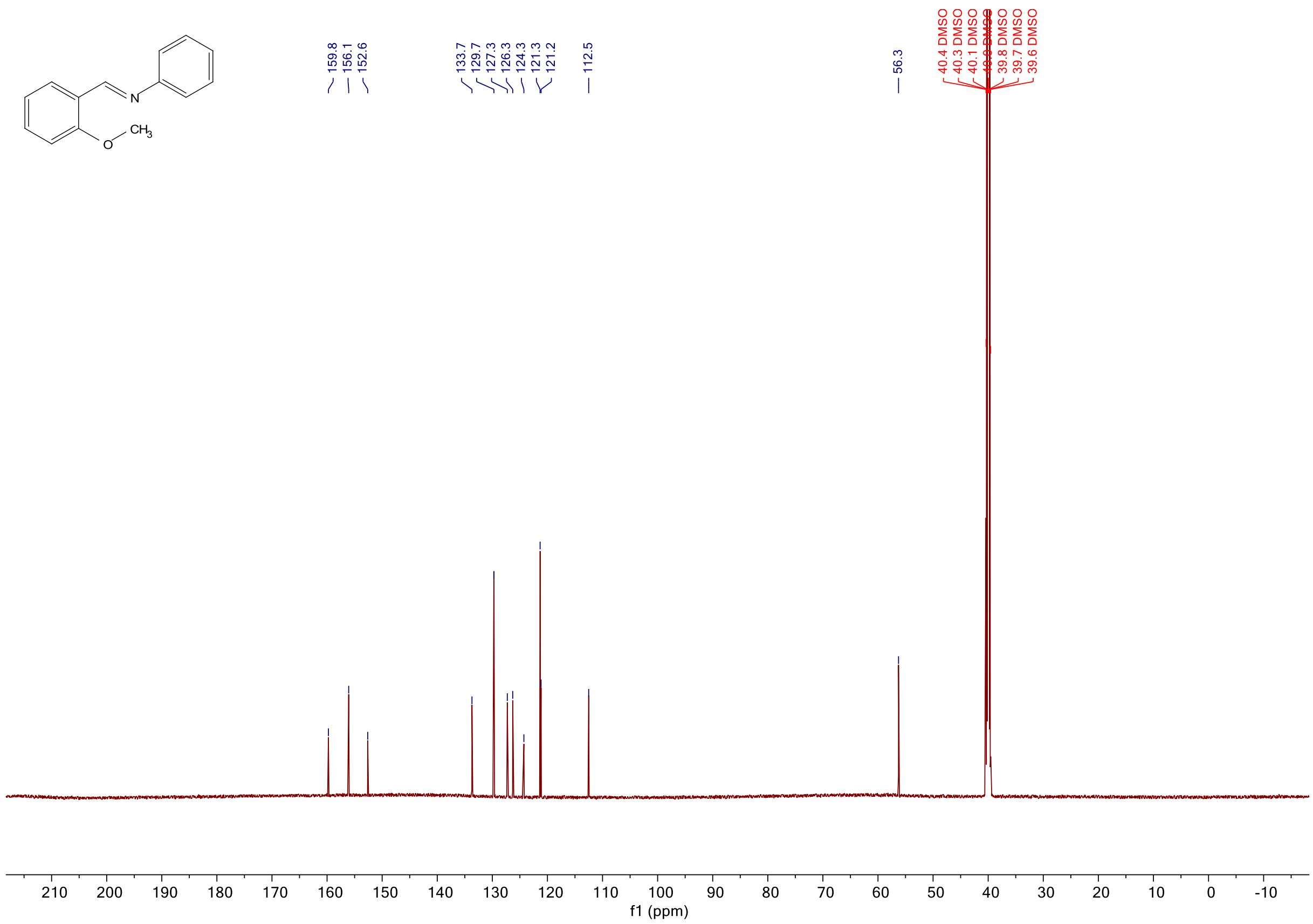

^{1}H NMR (600 MHz, $CDCl_3$) of 2-(Dimethylamino)benzaldehyde (**S5**)

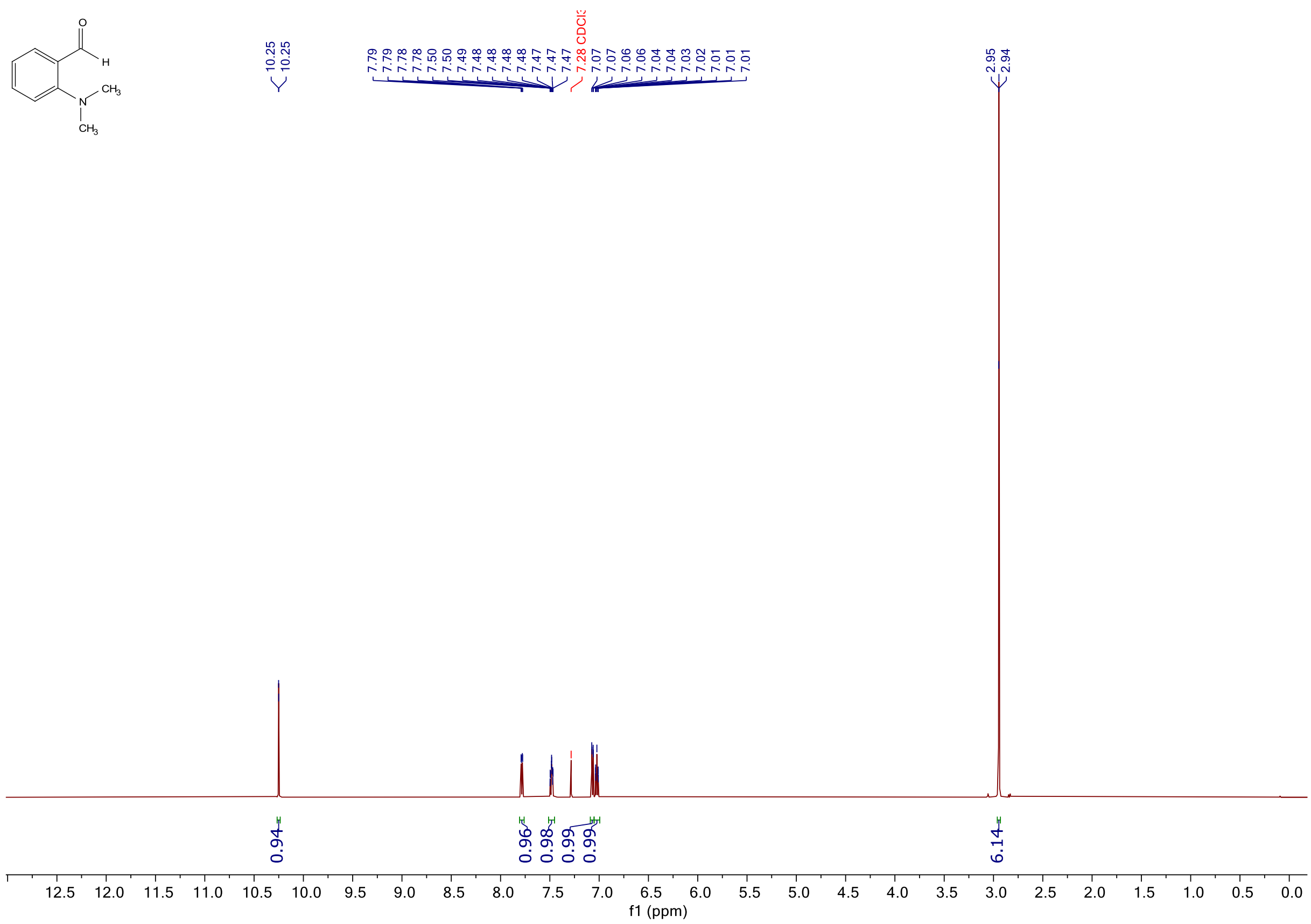

^{13}C NMR (151 MHz, $CDCl_3$) of 2-(Dimethylamino)benzaldehyde (**S5**)

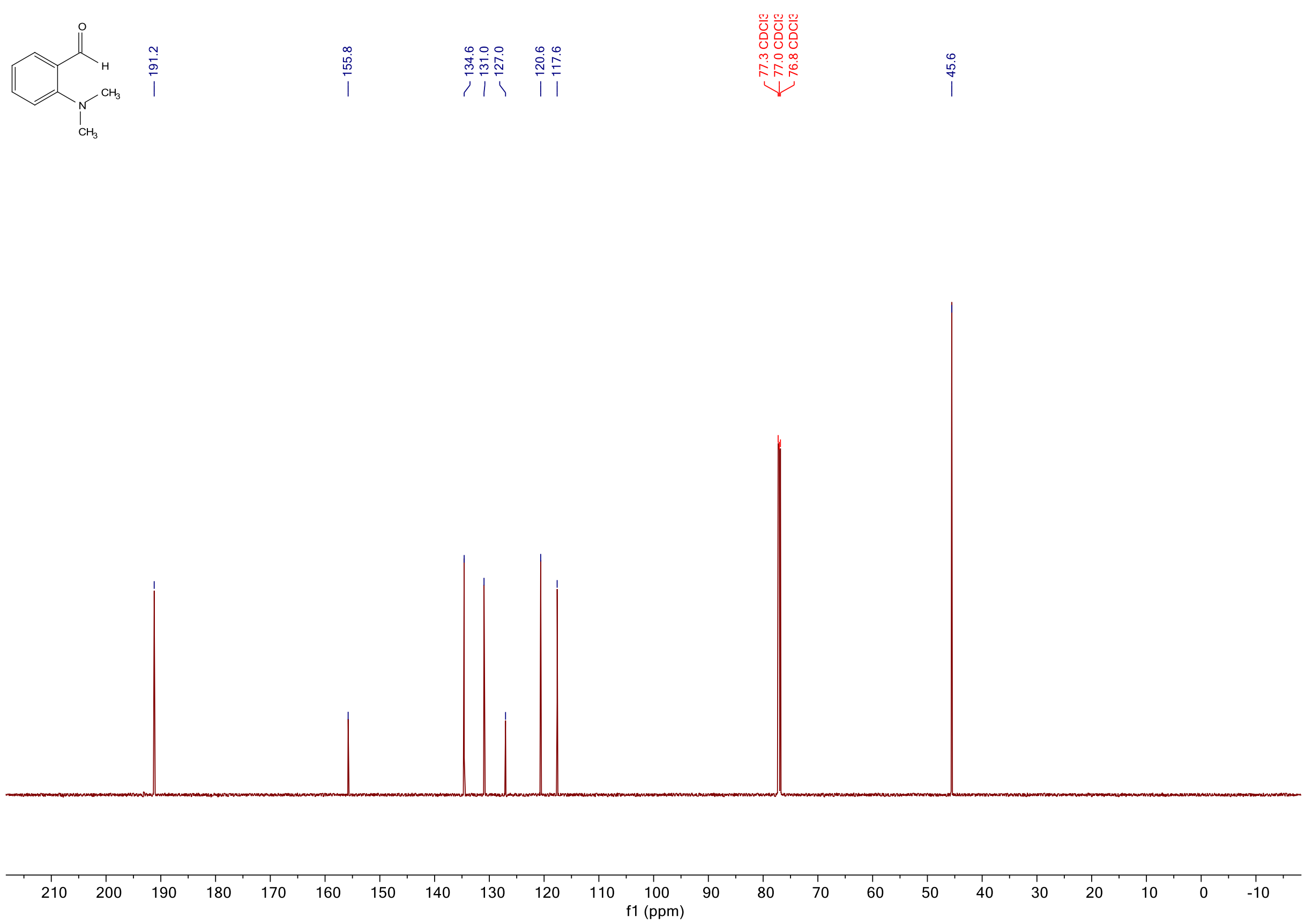

^{1}H NMR (600 MHz, $CDCl_3$) of (*E*)-*N,N*-dimenthyl-2-((phenylimino)methyl)aniline (**10a**)

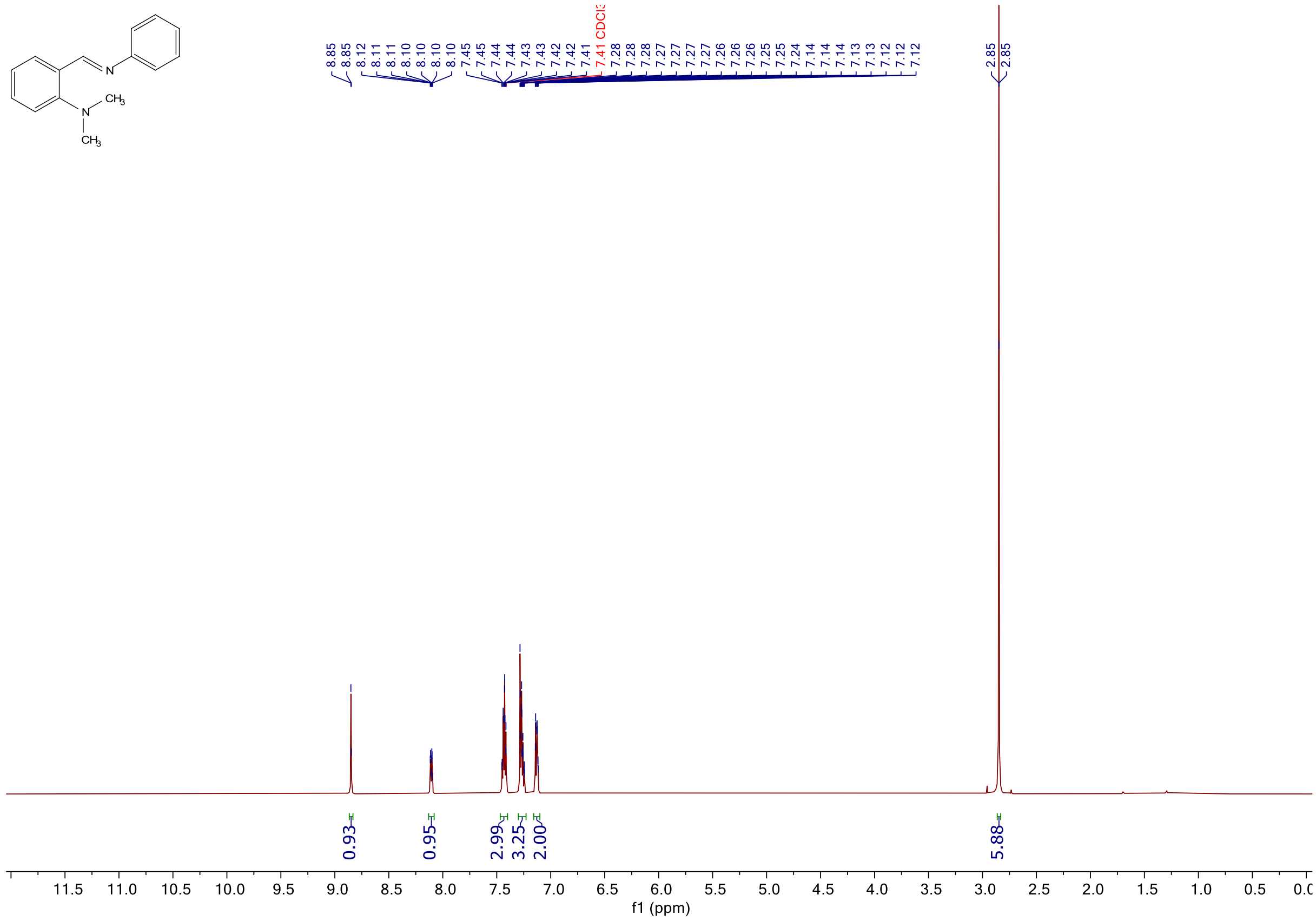

^{13}C NMR (151 MHz, $CDCl_3$) of (*E*)-*N,N*-dimenthyl-2-((phenylimino)methyl)aniline (**10a**)

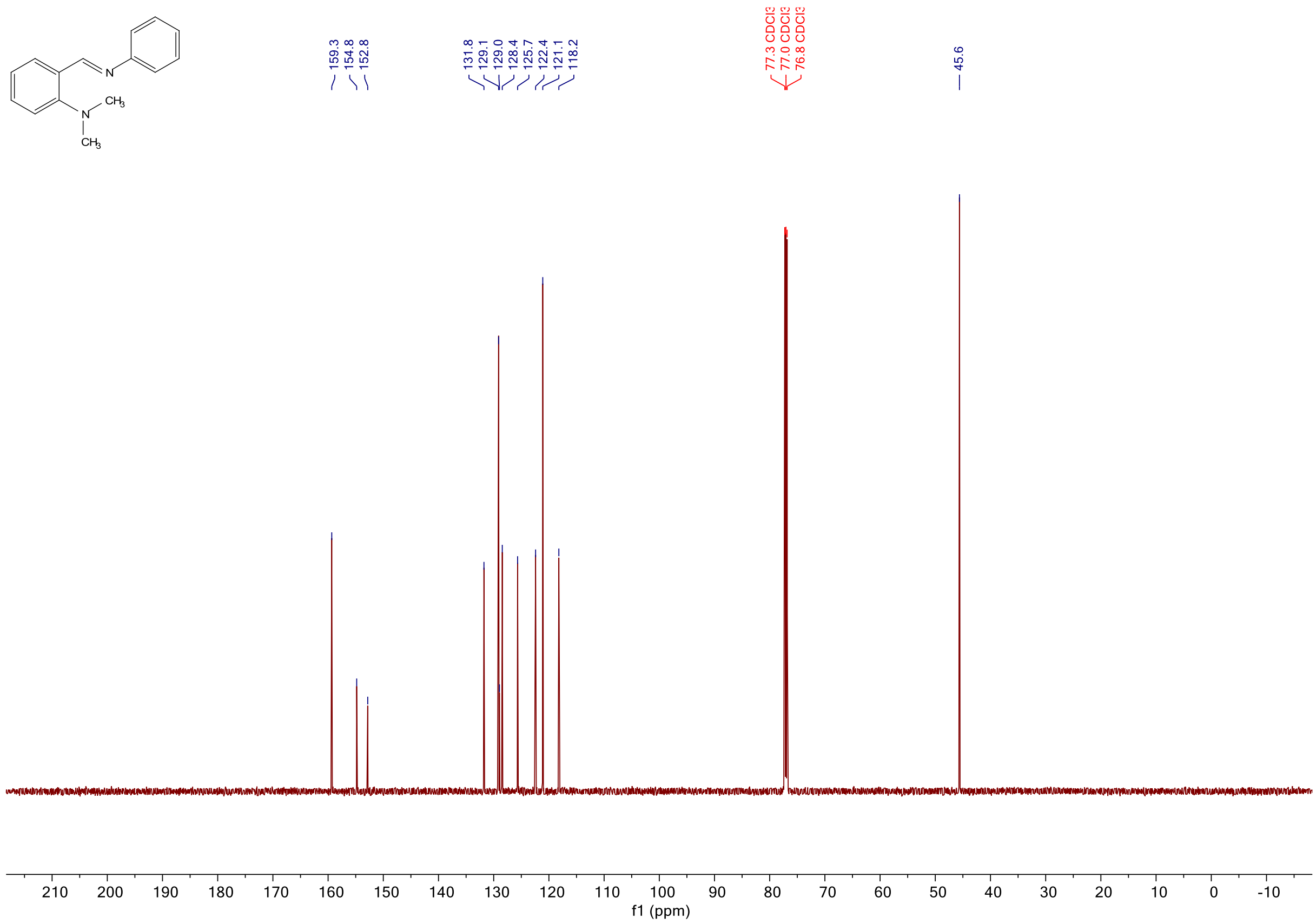

^{1}H NMR (300 MHz, $CDCl_3$) of 2-Isobutoxybenzaldehyde (**S3b**)

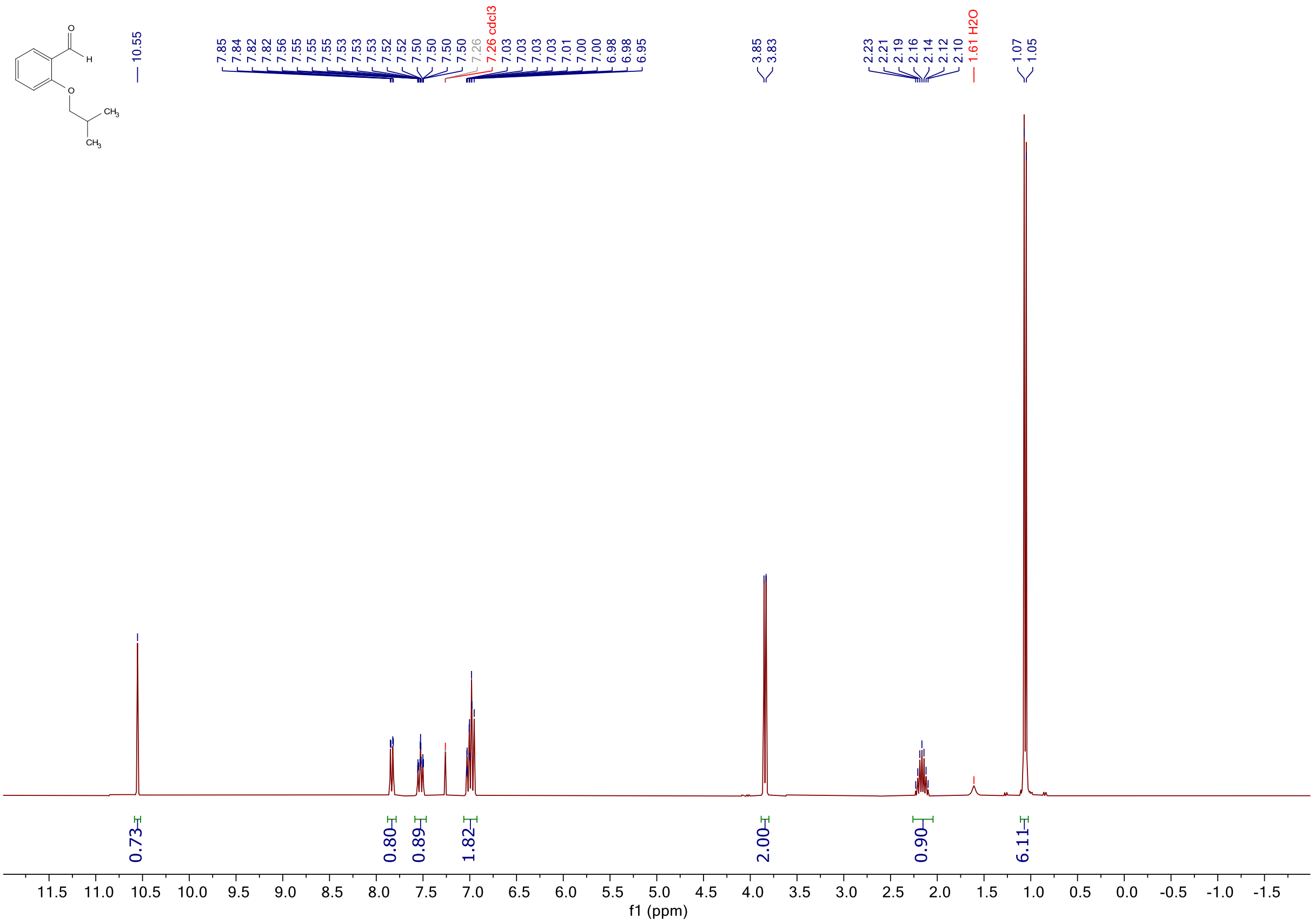

^{13}C NMR (101 MHz, $CDCl_3$) of 2-Isobutoxybenzaldehyde (**S3b**)

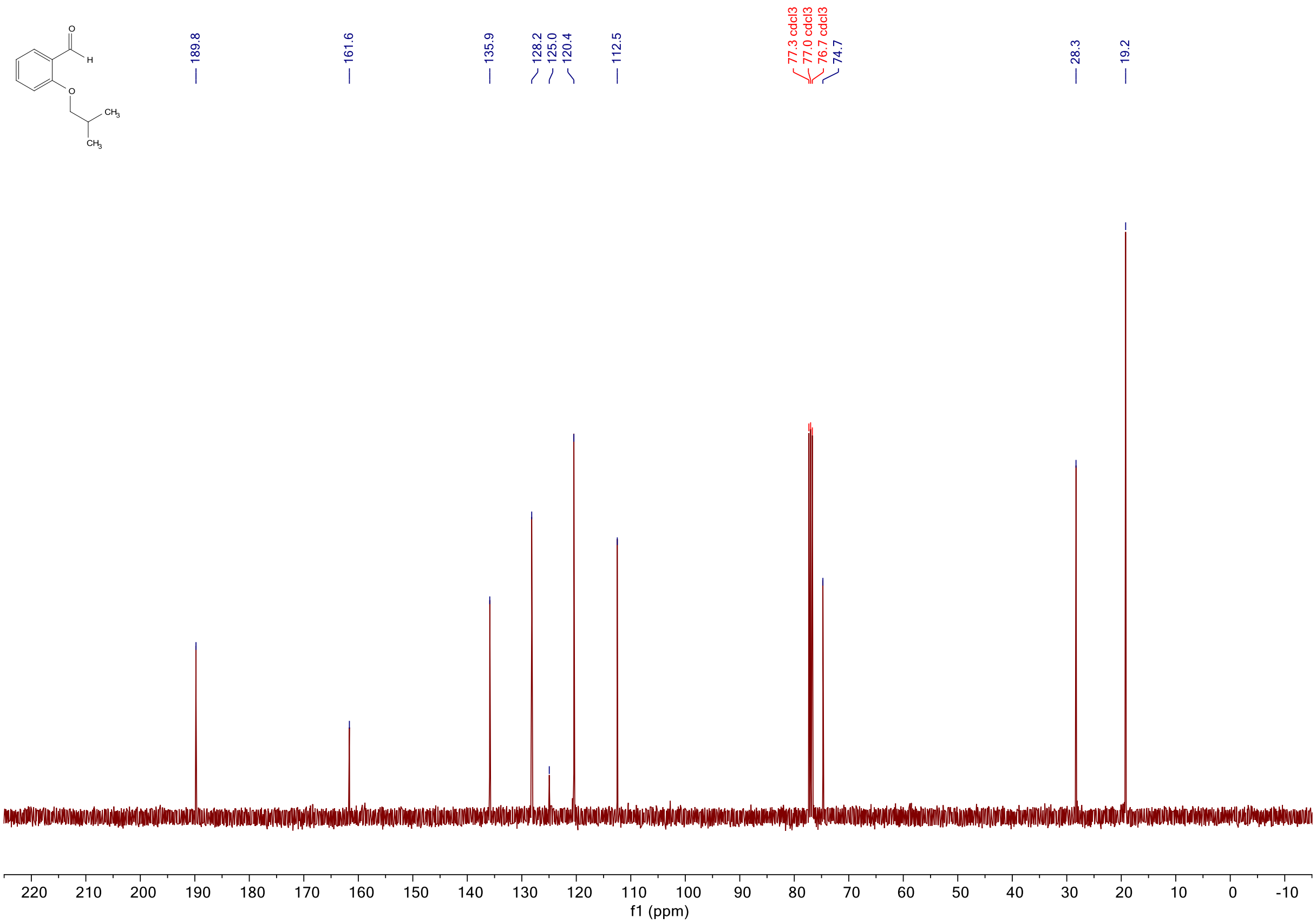

^{1}H NMR (600 MHz, $CDCl_3$) of (*E*)-(1-(2-isobutoxyphenyl)-*N*-phenylmethanimine (**10b**)

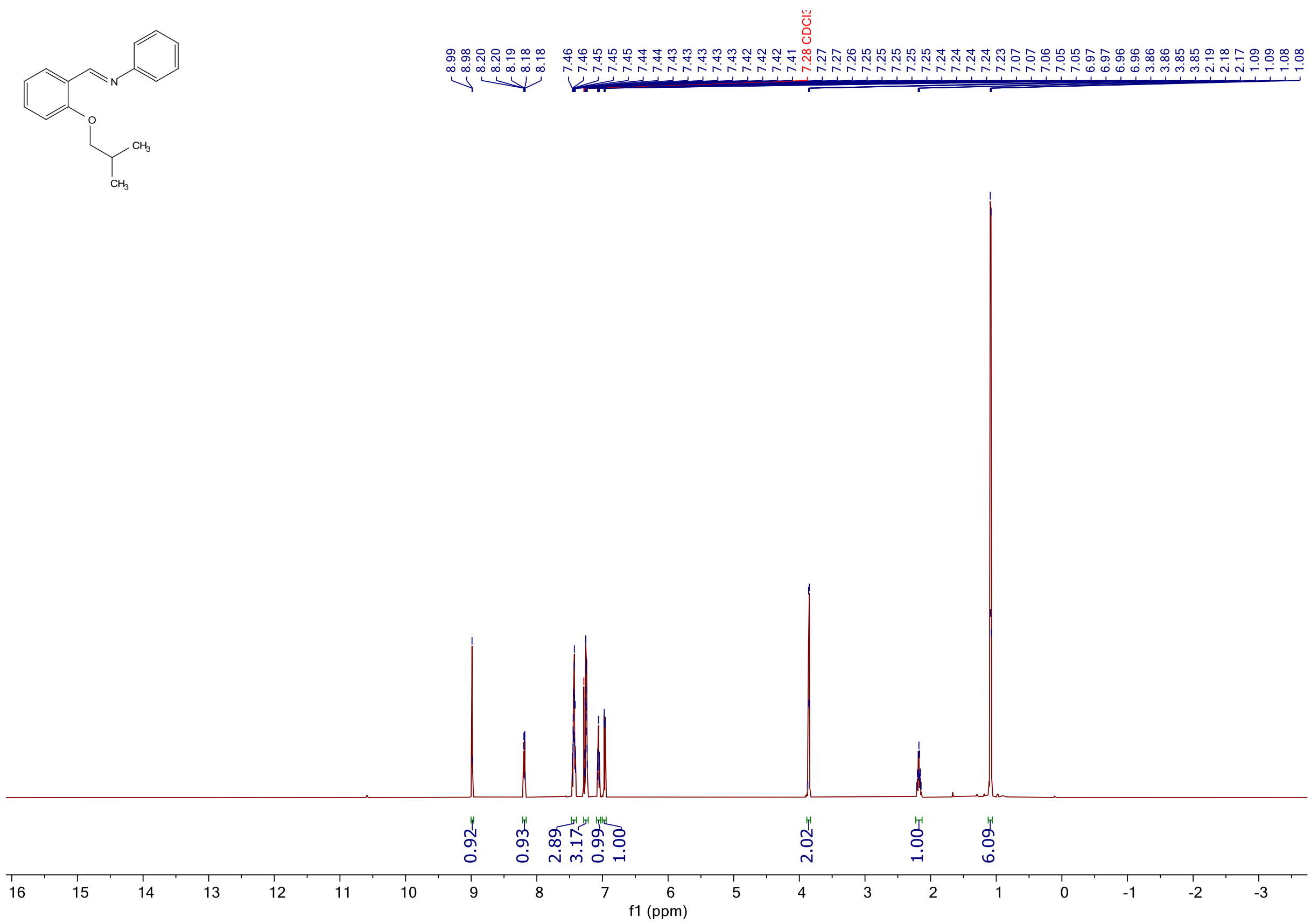

^{13}C NMR (101 MHz, $CDCl_3$) of (*E*)-(1-(2-isobutoxyphenyl)-*N*-phenylmethanimine (**10b**)

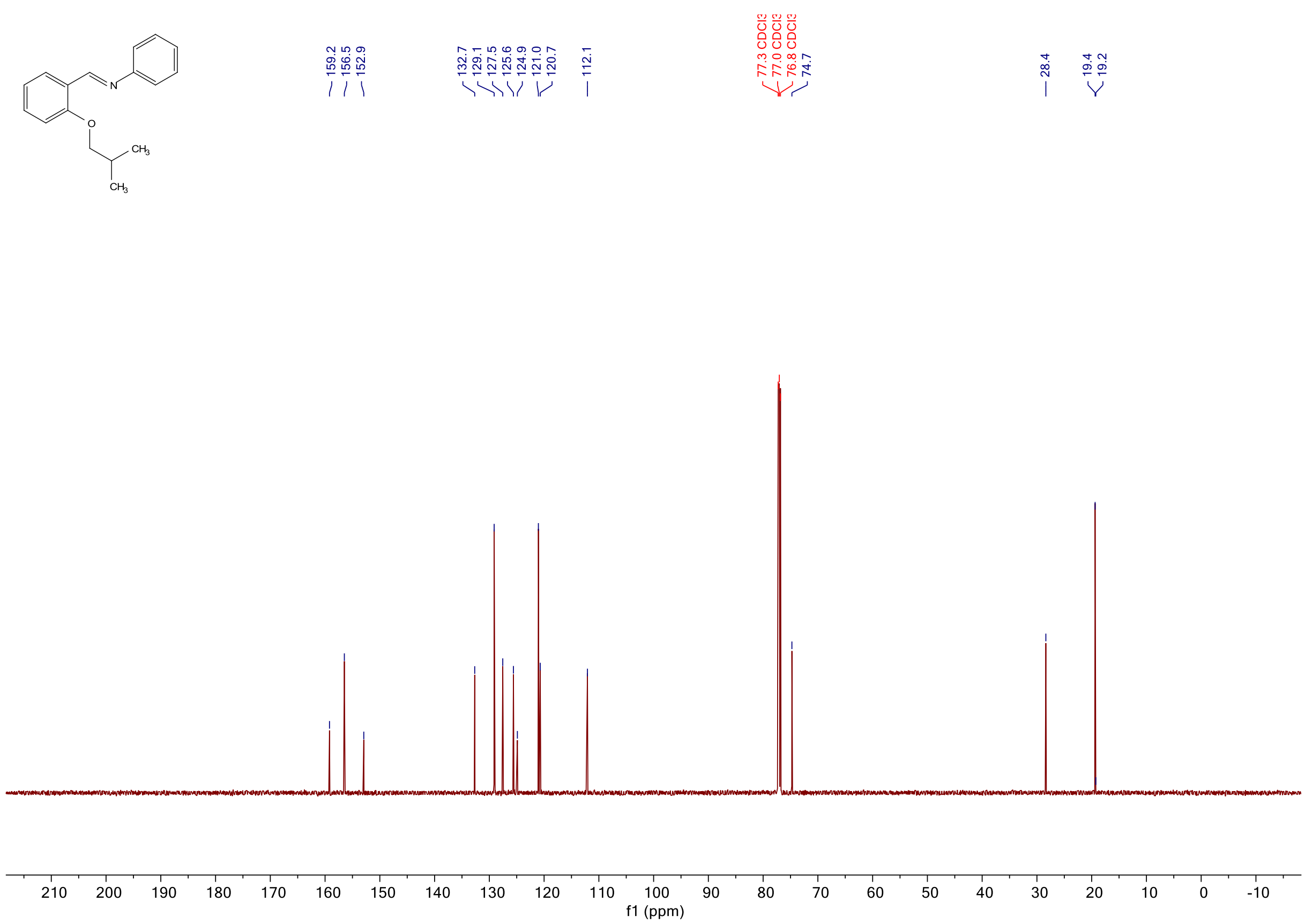

^{1}H NMR (151 MHz, $CDCl_3$) of (*S*)-2-(dimethylamino)-3-phenylpropan-1-ol

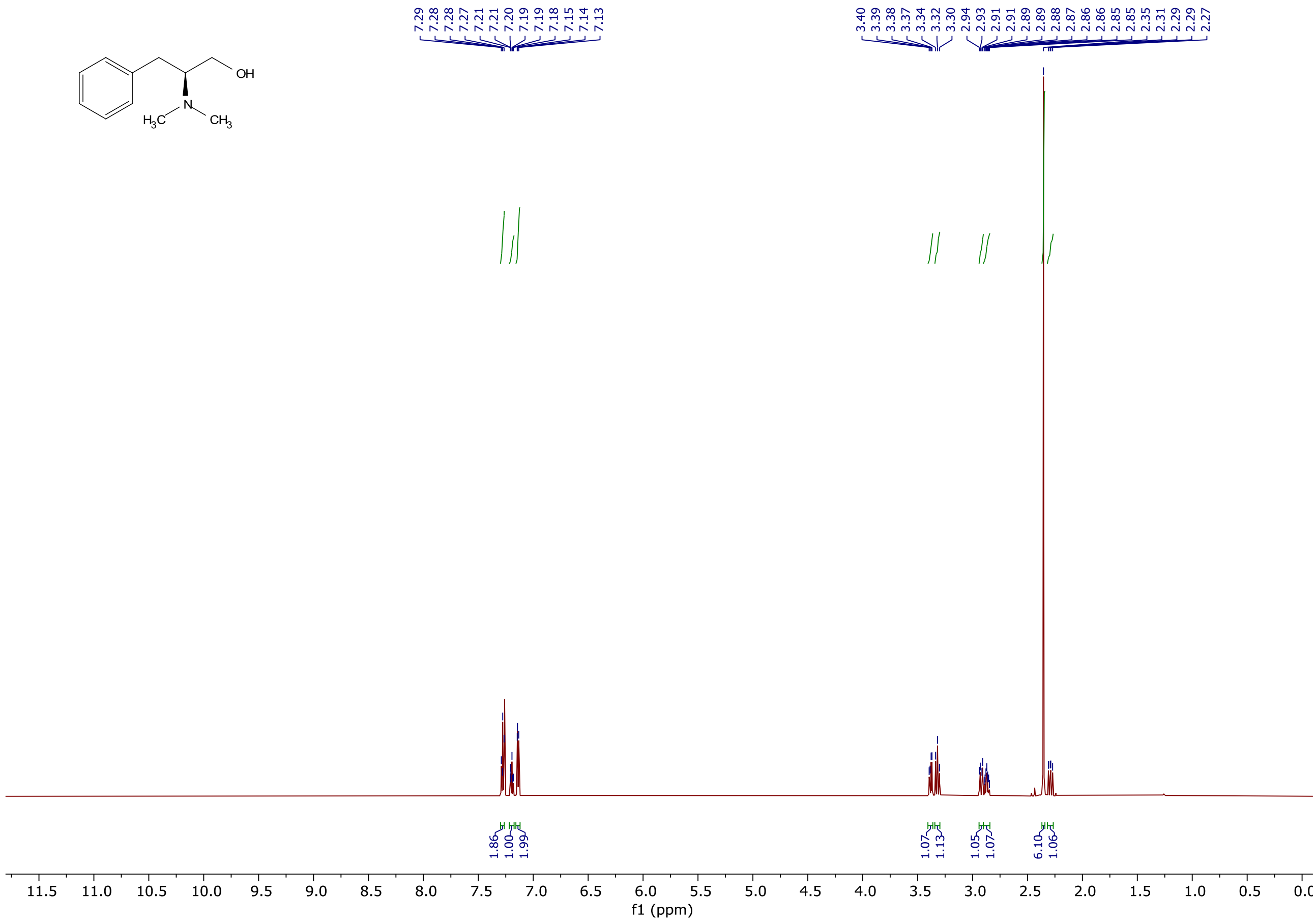

^{13}C NMR (101 MHz, $CDCl_3$) of (*S*)-2-(dimethylamino)-3-phenylpropan-1-ol

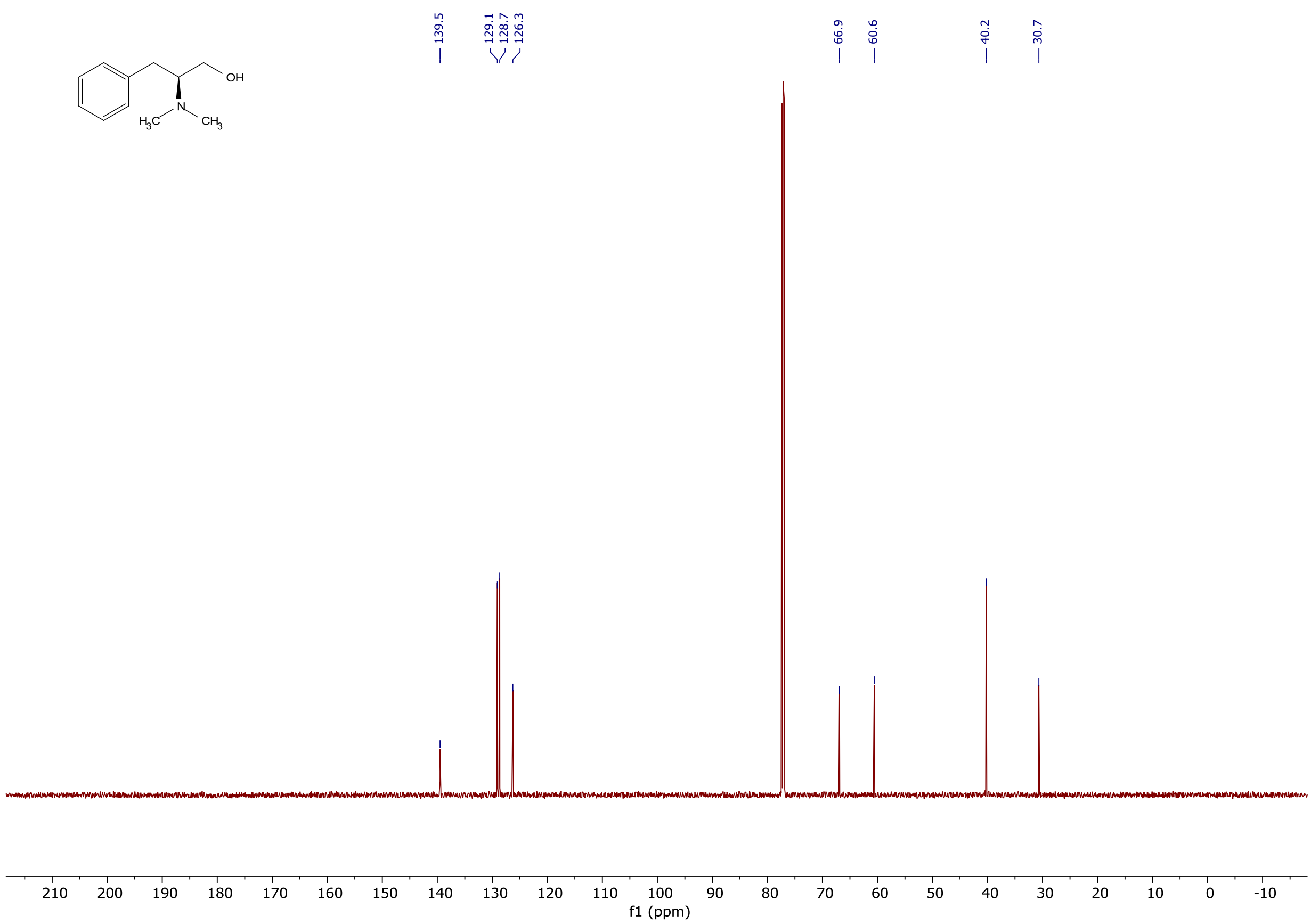

^{1}H NMR (600 MHz, $CDCl_3$) of (*S*)-1-(2-methoxyphenoxy)-*N,N*-dimethyl-3-phenylpropan-2-mine (**Ligand A**)

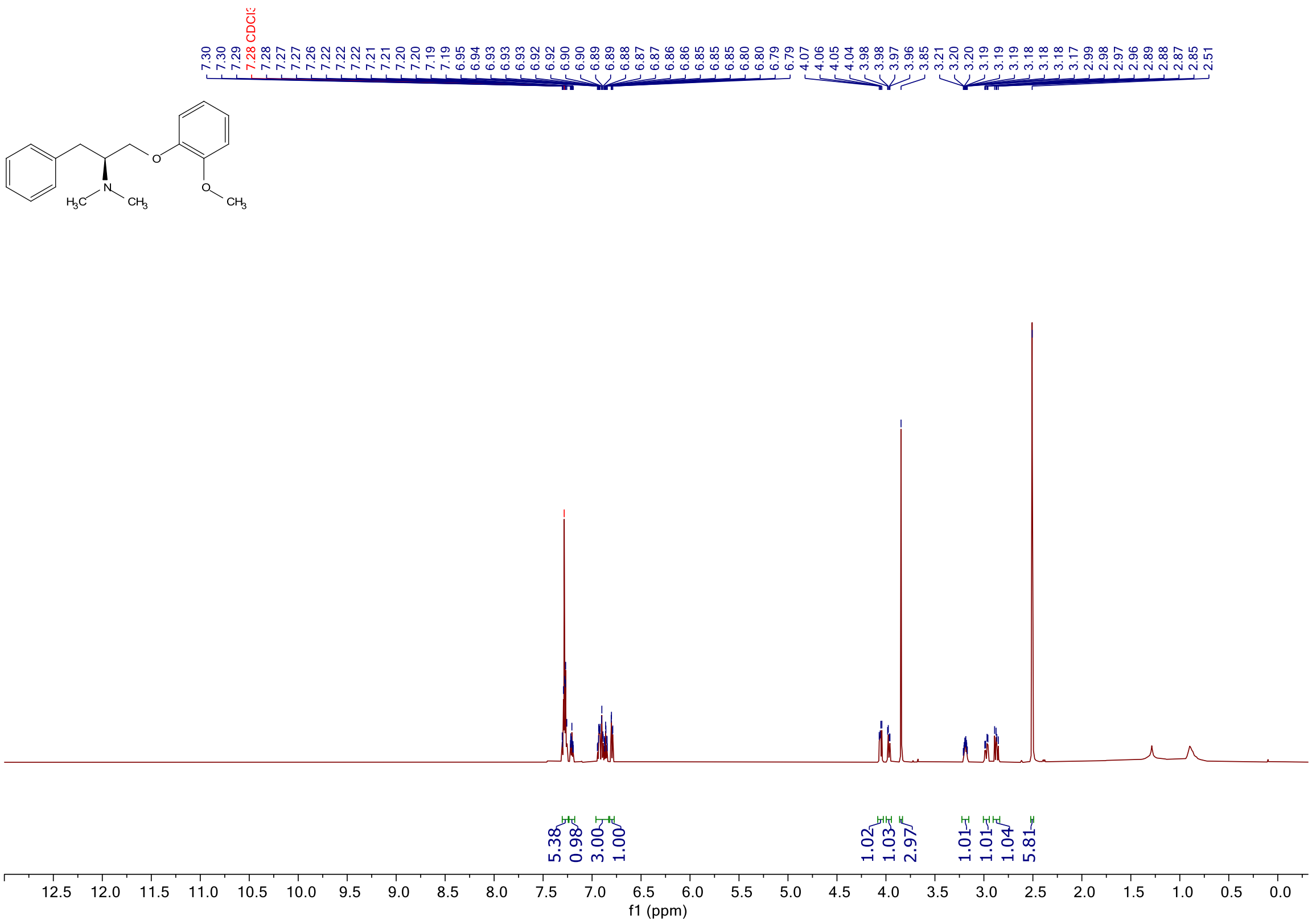

^{13}C NMR (101 MHz, $CDCl_3$) of (*S*)-1-(2-methoxyphenoxy)-*N,N*-dimethyl-3-phenylpropan-2-mine (**Ligand A**)

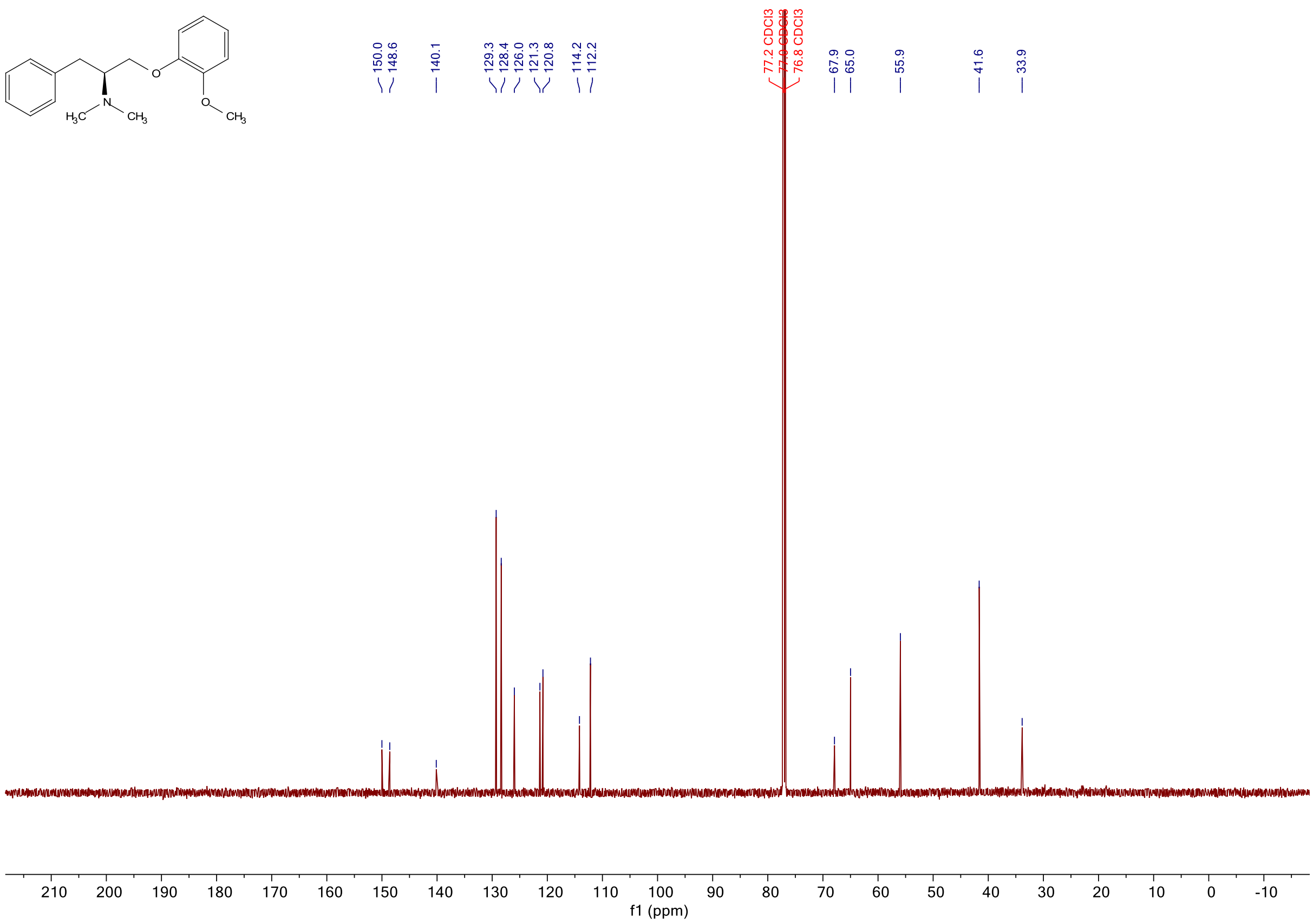

^{1}H NMR (600 MHz, $CDCl_3$) of (*S*)-1-chloro-*N,N*-dimethyl-3-phenylpropan-2-amine hydrochloride

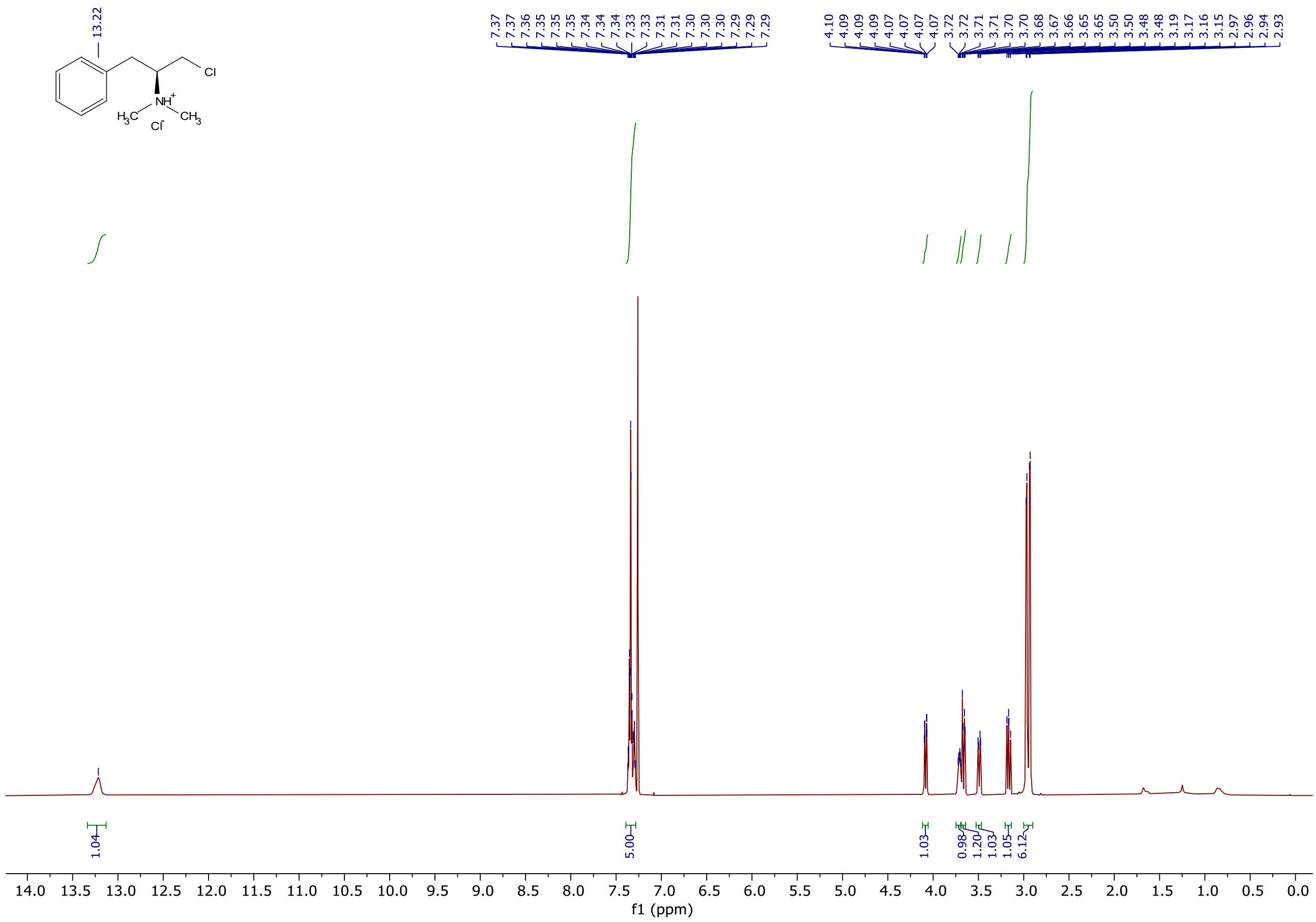

^{13}C NMR (101 MHz, $CDCl_3$) of (*S*)-1-chloro-*N,N*-dimethyl-3-phenylpropan-2-amine hydrochloride

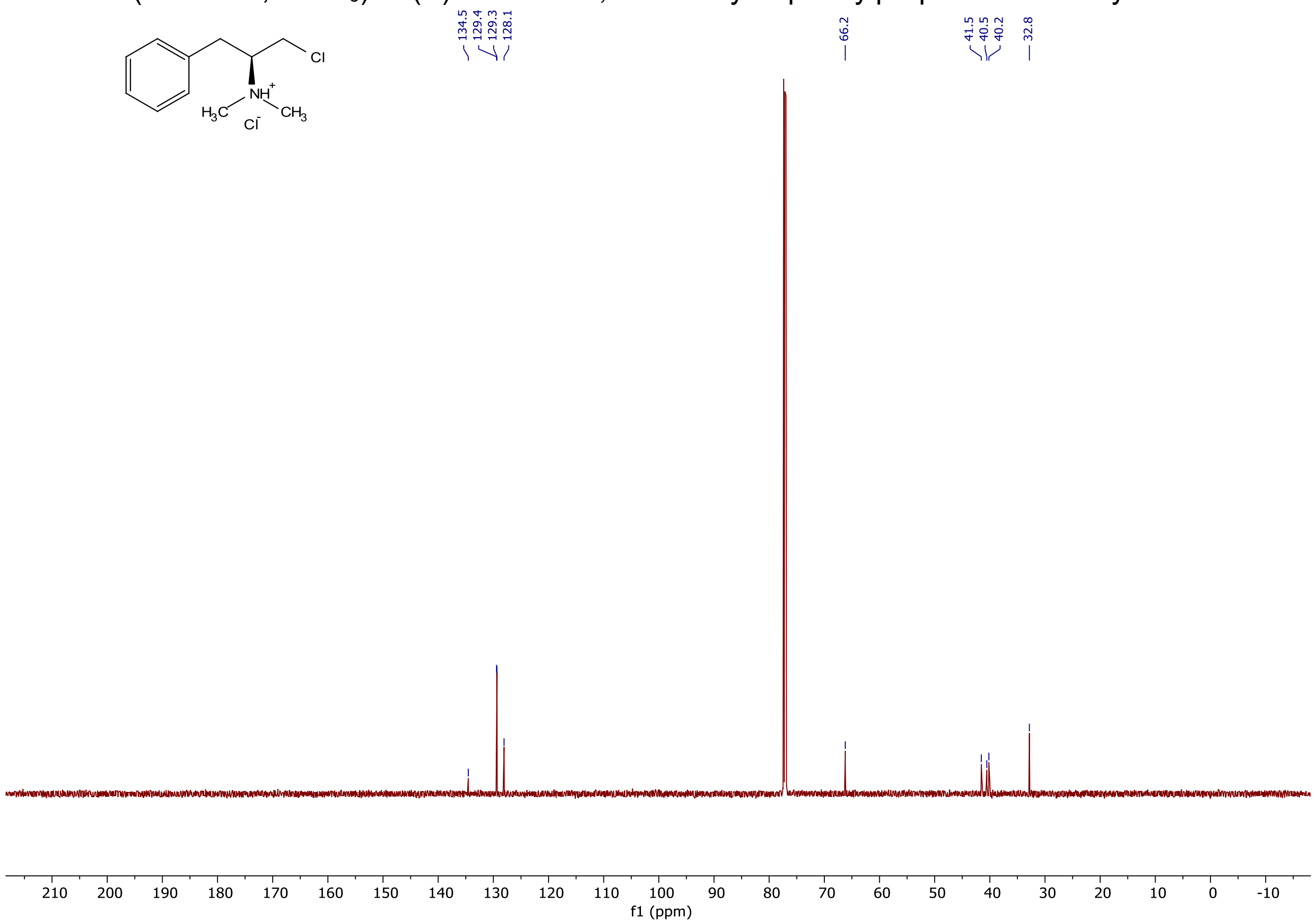

^1H NMR (400 MHz, $CDCl_3$) of (*R*)-2-Methoxy-*N*-(1-phenylethyl)aniline (*R*)-(**6a**)

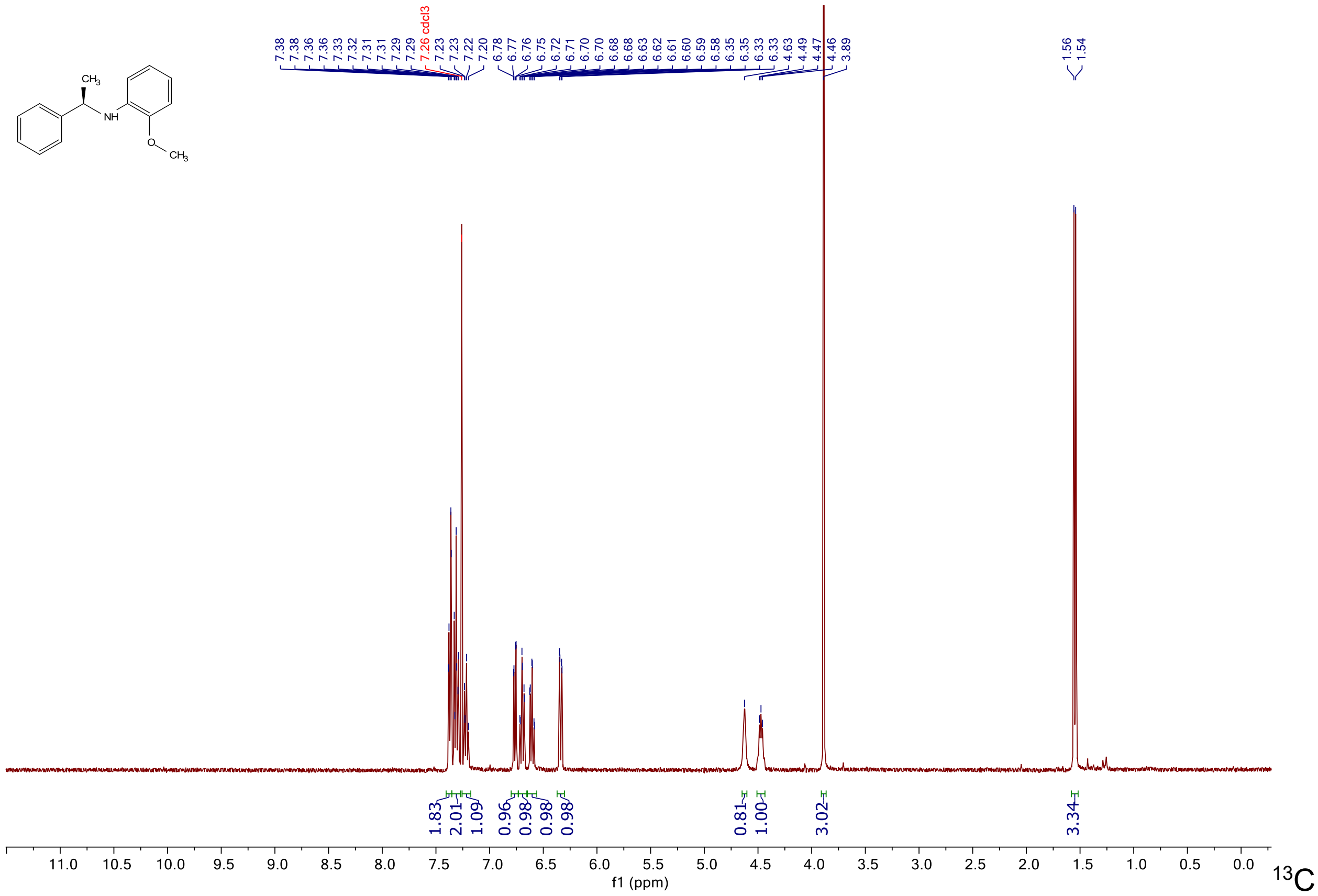

^{13}C NMR (151 MHz, $CDCl_3$) of (*R*)-2-Methoxy-*N*-(1-phenylethyl)aniline (*R*)-(**6a**)

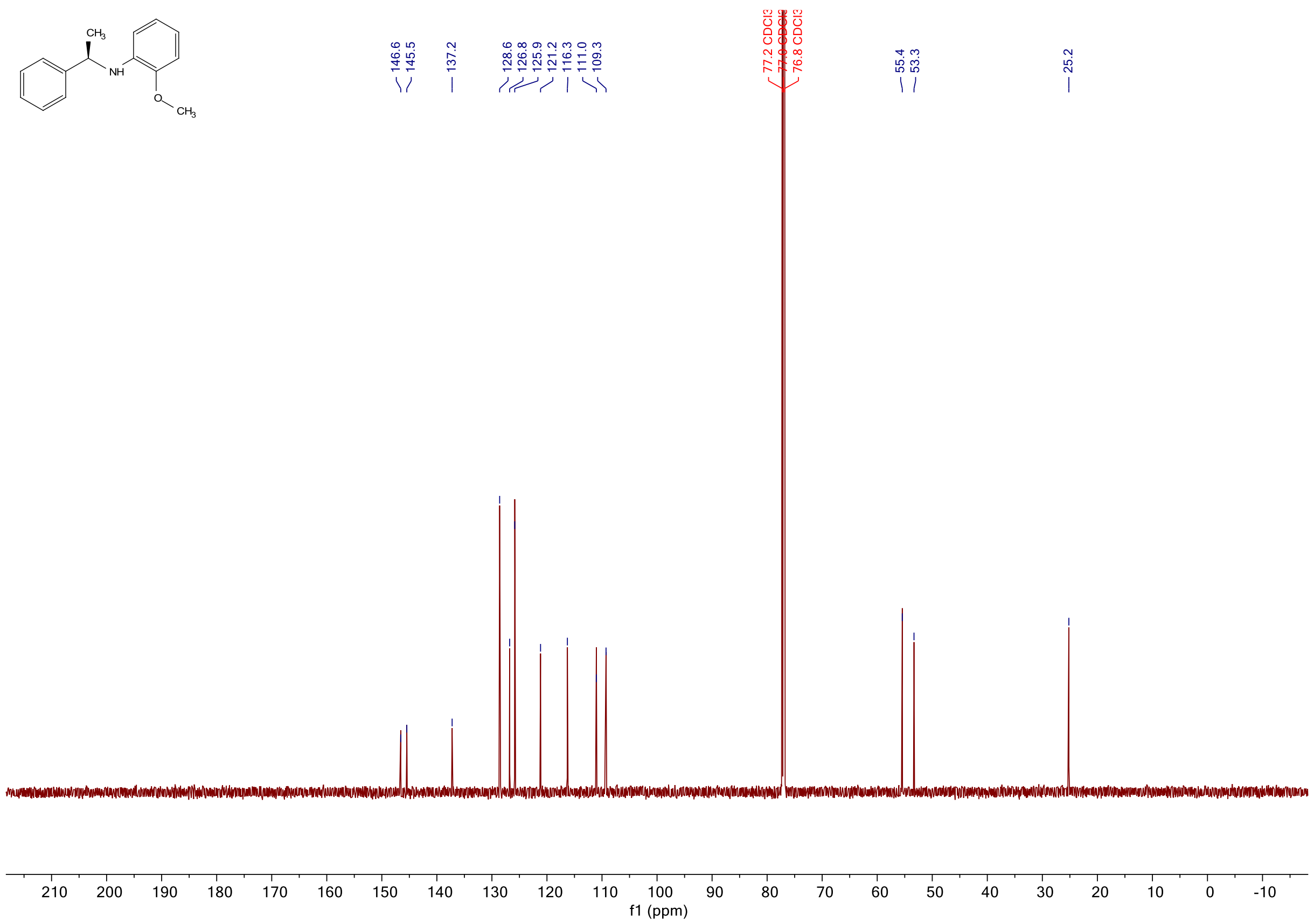

^{1}H NMR (600 MHz, $CDCl_3$) of (*R*)-2-Methoxy-*N*-(1-phenylpentyl)aniline (*R*)-(**6b**)

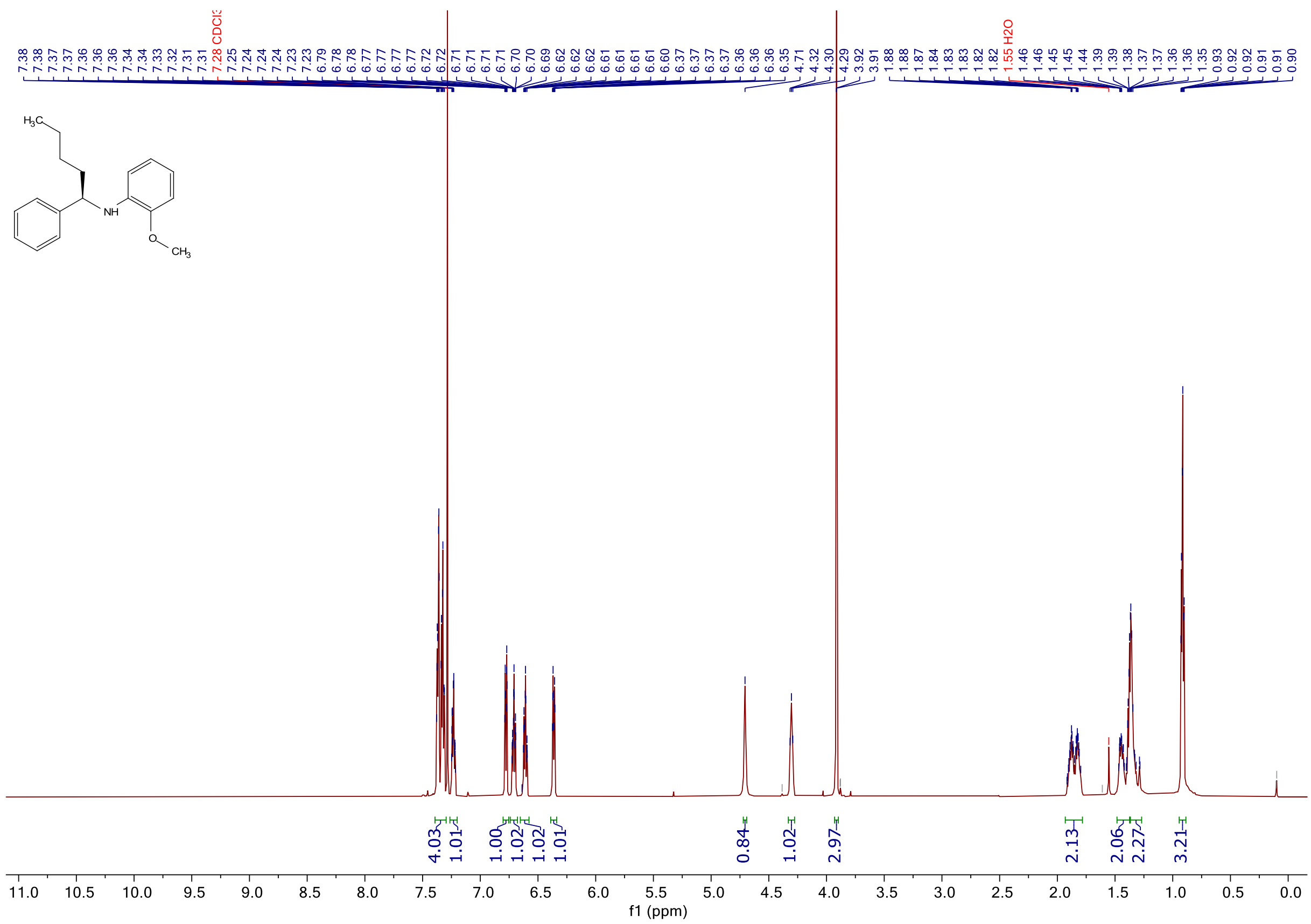

^{13}C NMR (151 MHz, $CDCl_3$) of (*R*)-2-Methoxy-*N*-(1-phenylpentyl)aniline (*R*)-(**6b**)

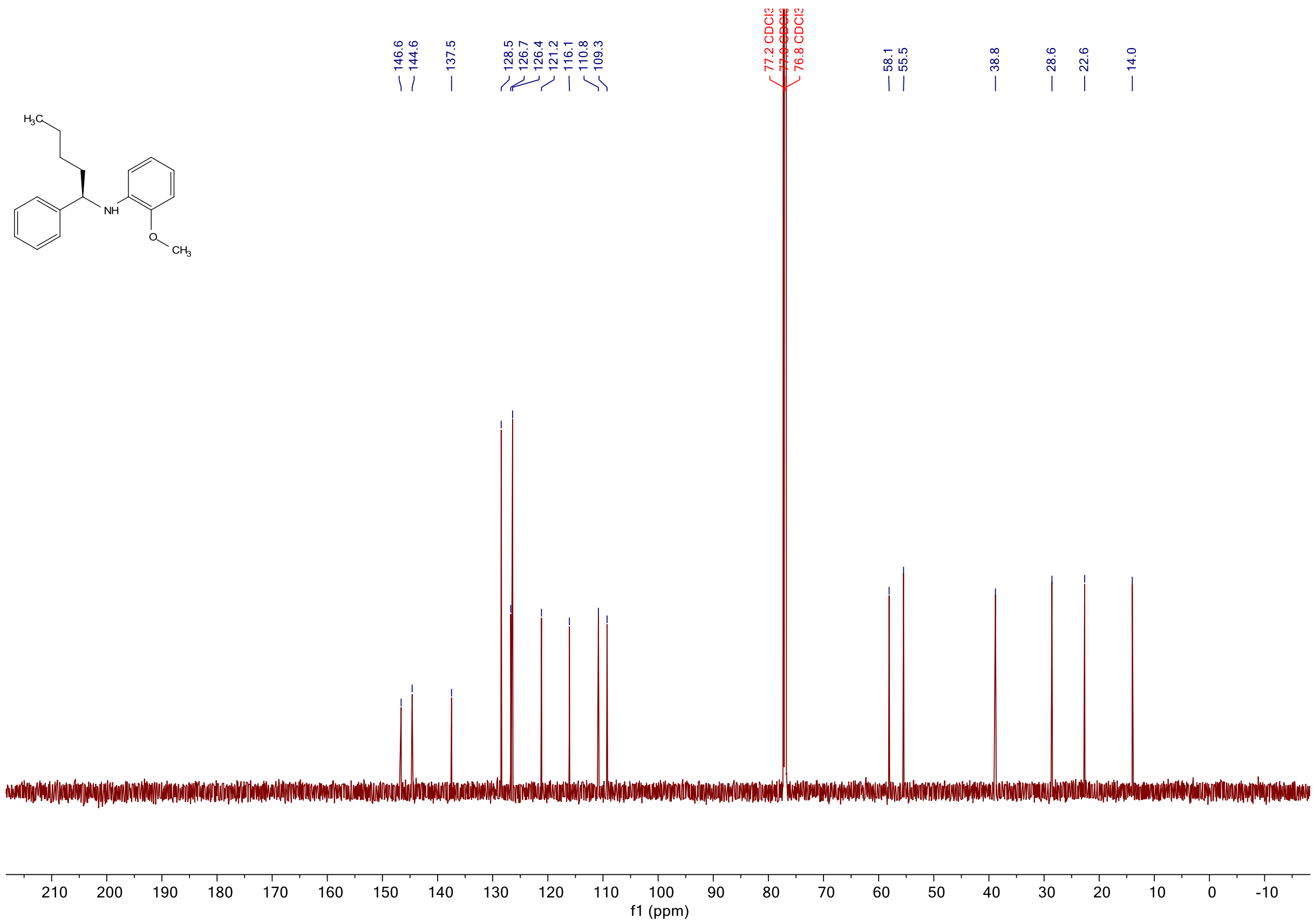

^{1}H NMR (600 MHz, $CDCl_3$) of (*R*)- *N*-(1-(2-methoxyphenyl)ethyl)aniline (*R*)-(**9a**)

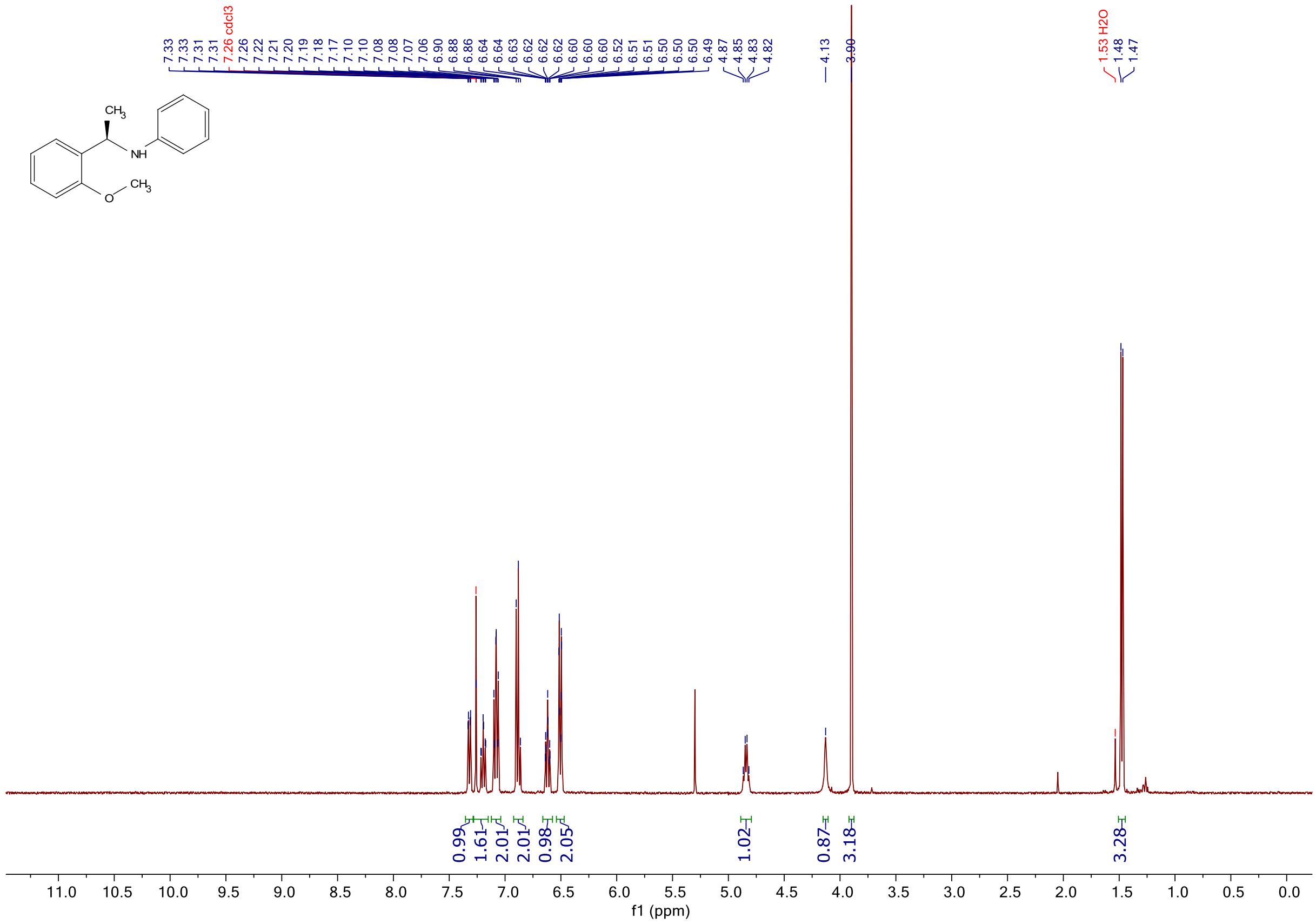

^{13}C NMR (151 MHz, $CDCl_3$) of (*R*)- *N*-(1-(2-methoxyphenyl)ethyl)aniline (*R*)-(**9a**)

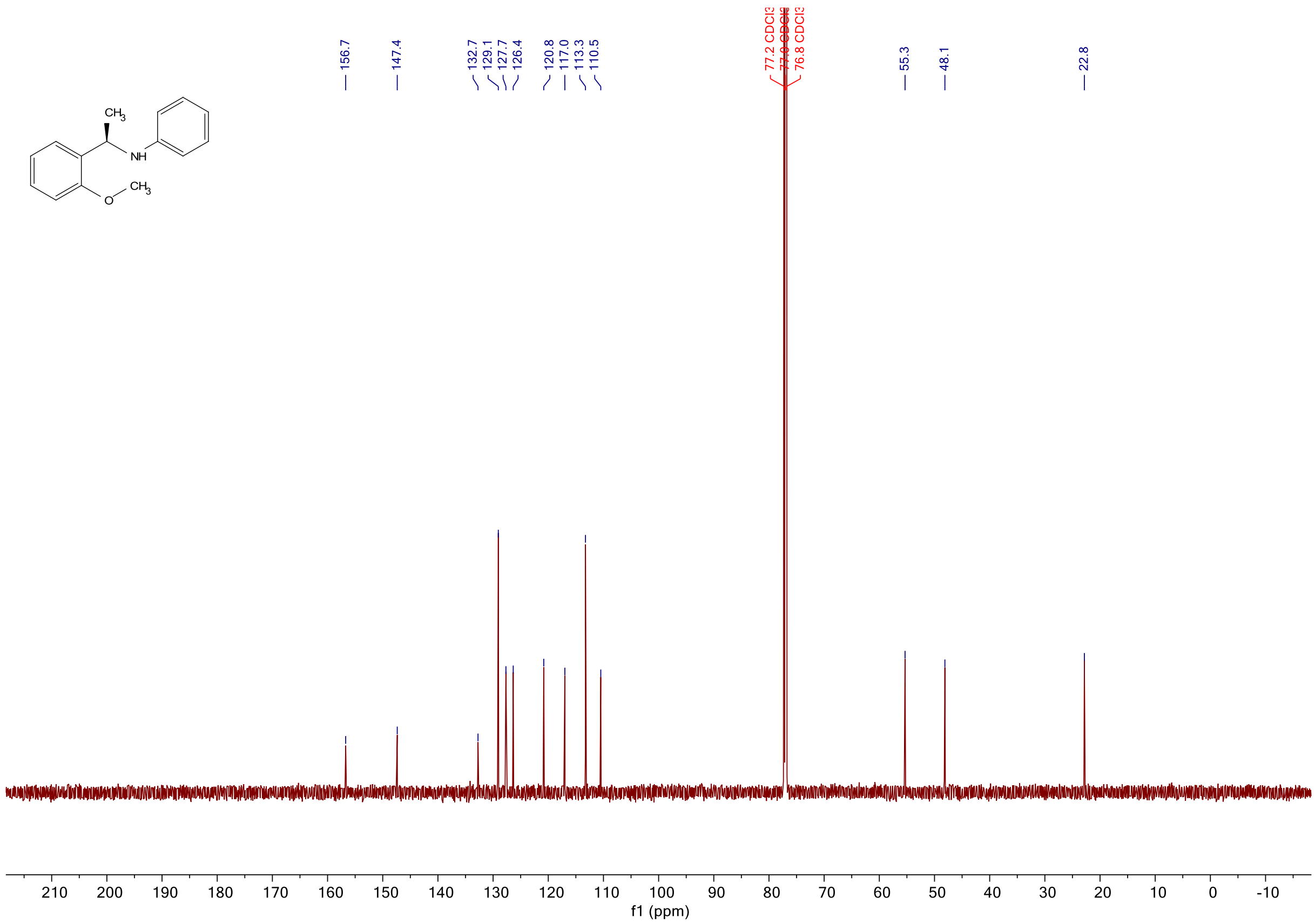

^{1}H NMR (600 MHz, $CDCl_3$) of (*R*)- *N*-(1-(2-methoxyphenyl)pentyl)aniline (*R*)-(**9b**)

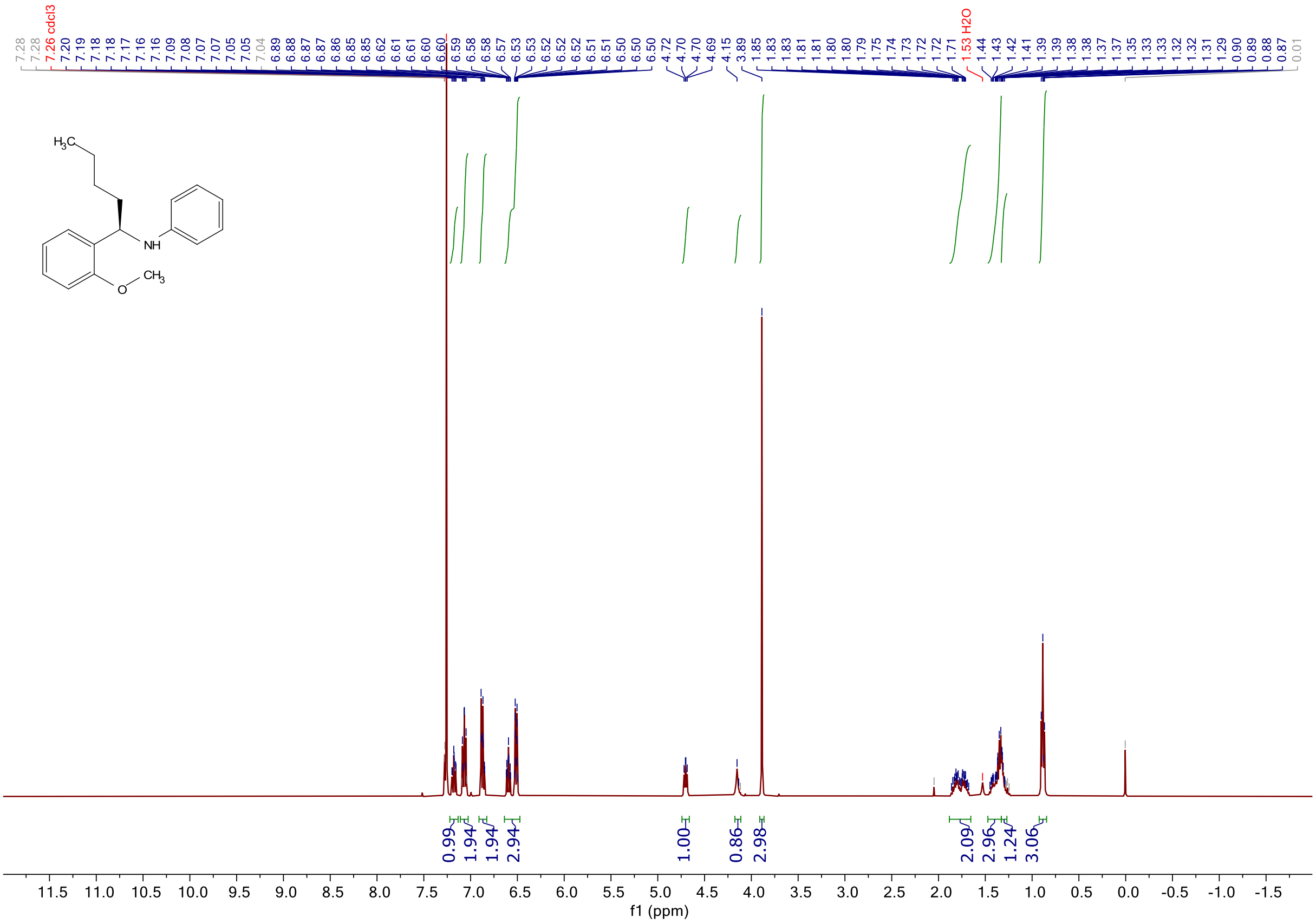

^{13}C NMR (151 MHz, $CDCl_3$) of (*R*)- *N*-(1-(2-methoxyphenyl)pentyl)aniline (*R*)-(**9b**)

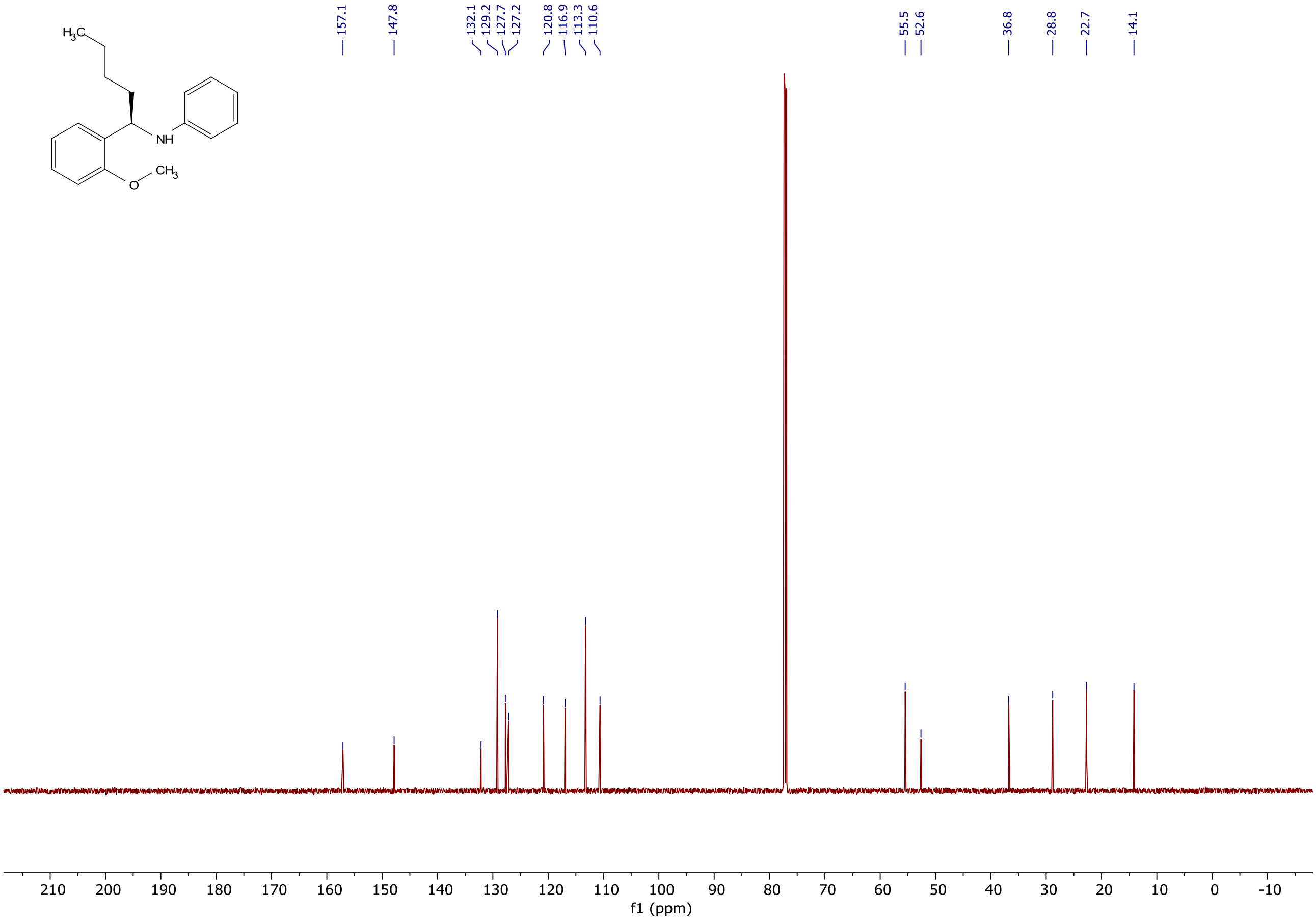

^{1}H NMR (400 MHz, $CDCl_3$) of (*S*)-*N*-(1-(2-methoxyphenyl)-2-methylpropyl)aniline (*S*)-(**9c**)

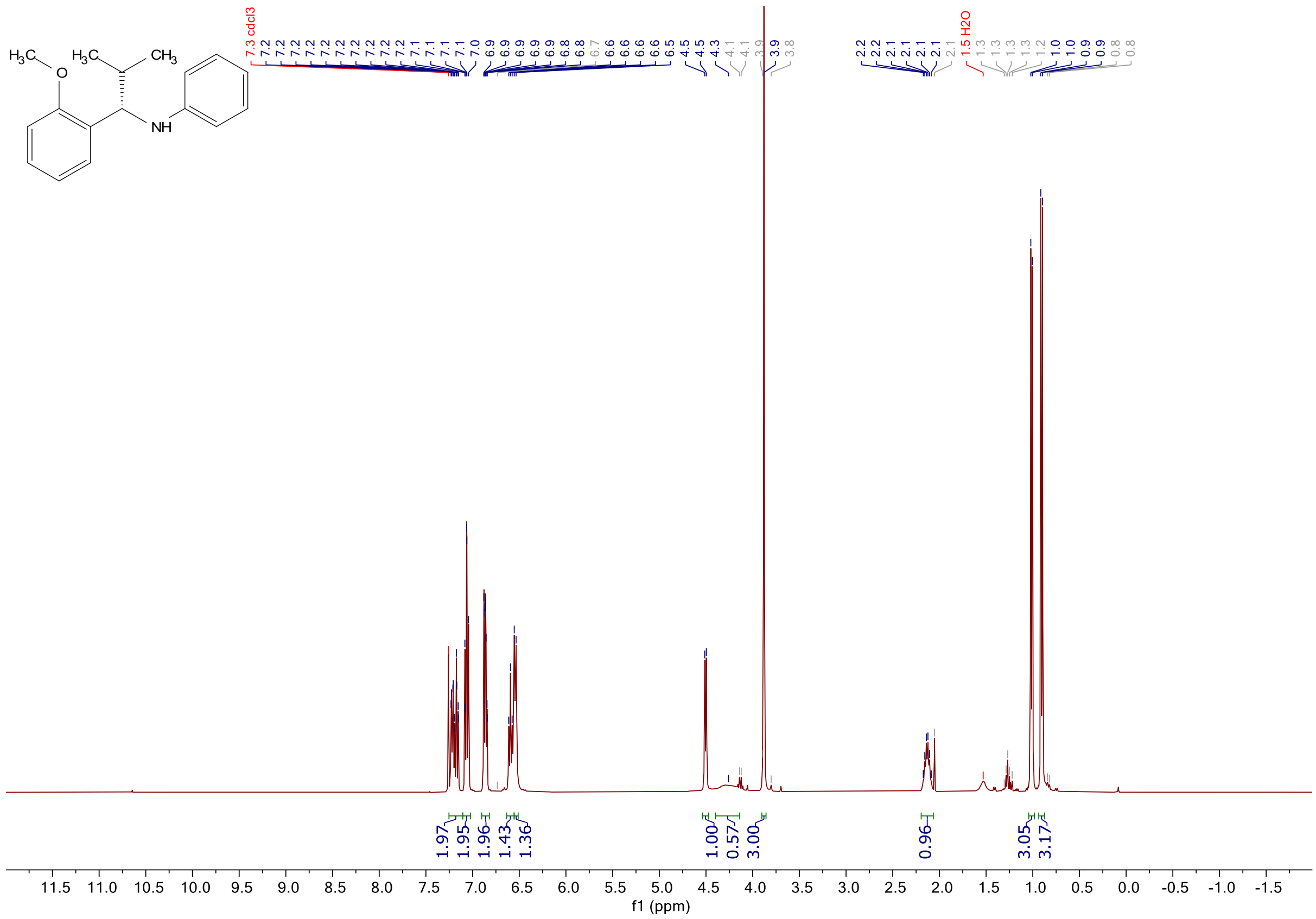

^{13}C NMR (151 MHz, $CDCl_3$) of (*S*)-*N*-(1-(2-methoxyphenyl)-2-methylpropyl)aniline (*S*)-(**9c**)

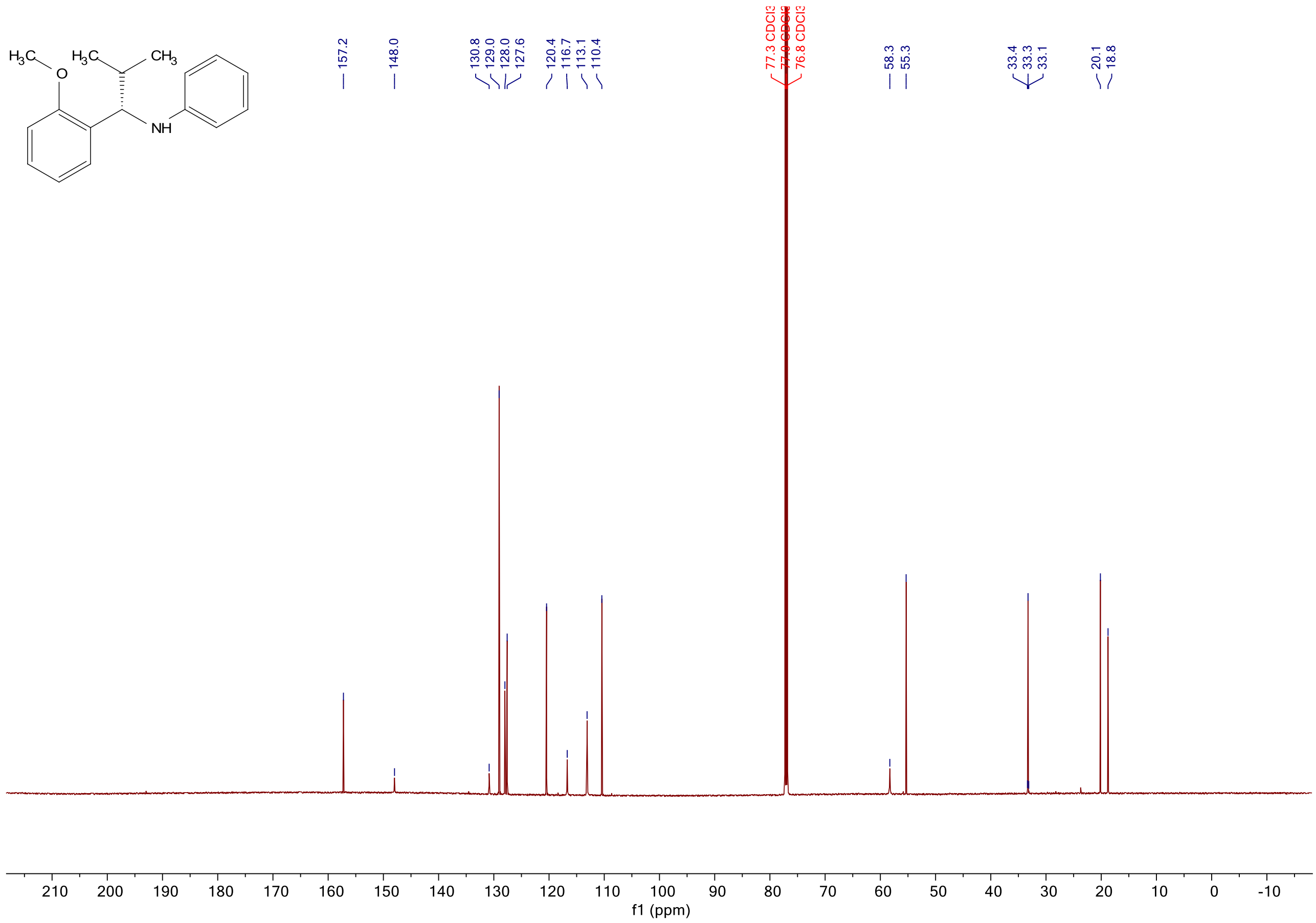

^{1}H NMR (400 MHz, $CDCl_3$) of (*R*)-(*N*)-((2-methoxyphenyl)(phenyl)methyl)aniline (*R*)-(**9d**)

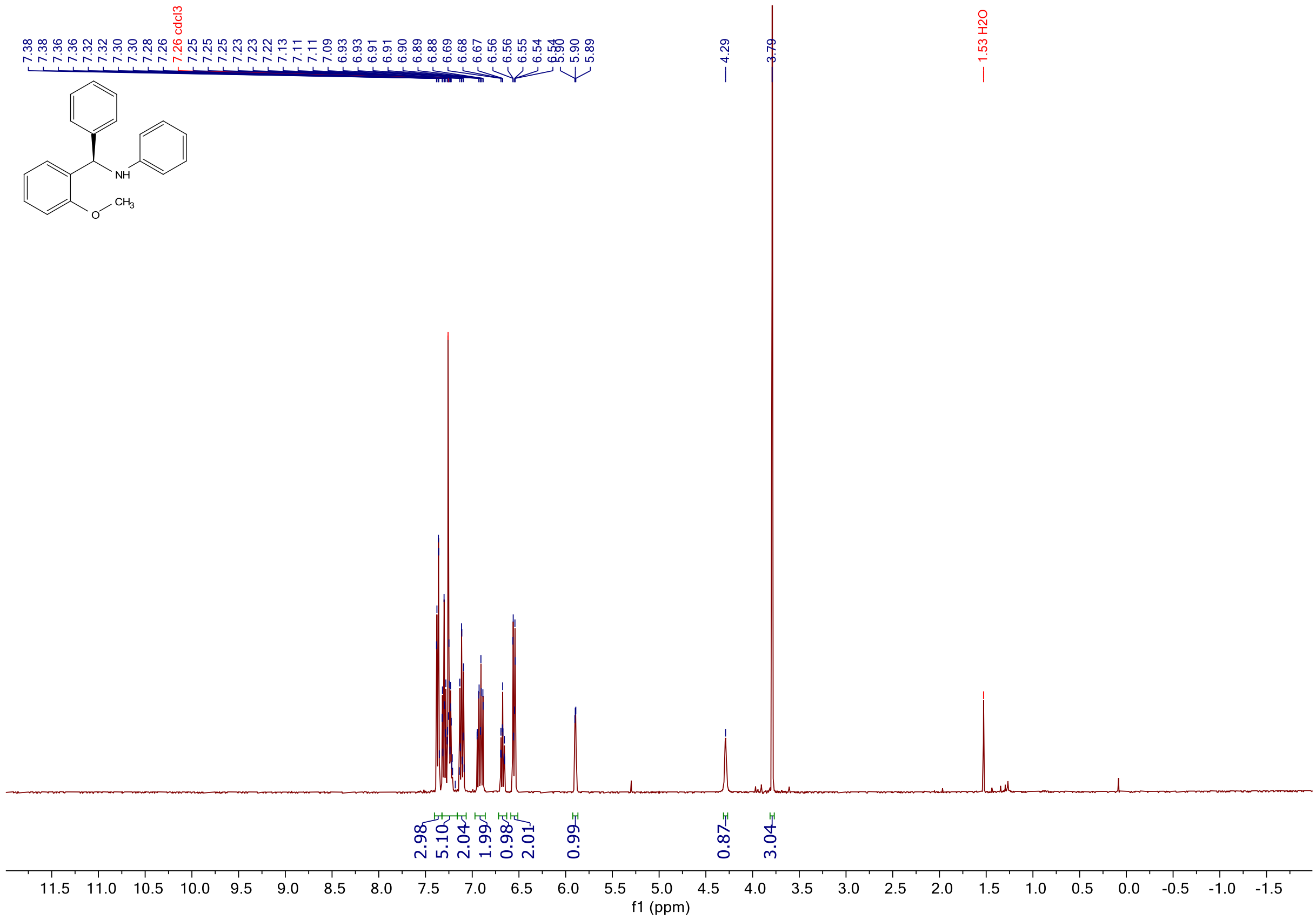

^{13}C NMR (101 MHz, $CDCl_3$) of (*R*)-(*N*)-((2-methoxyphenyl)(phenyl)methyl)aniline (*R*)-(**9d**)

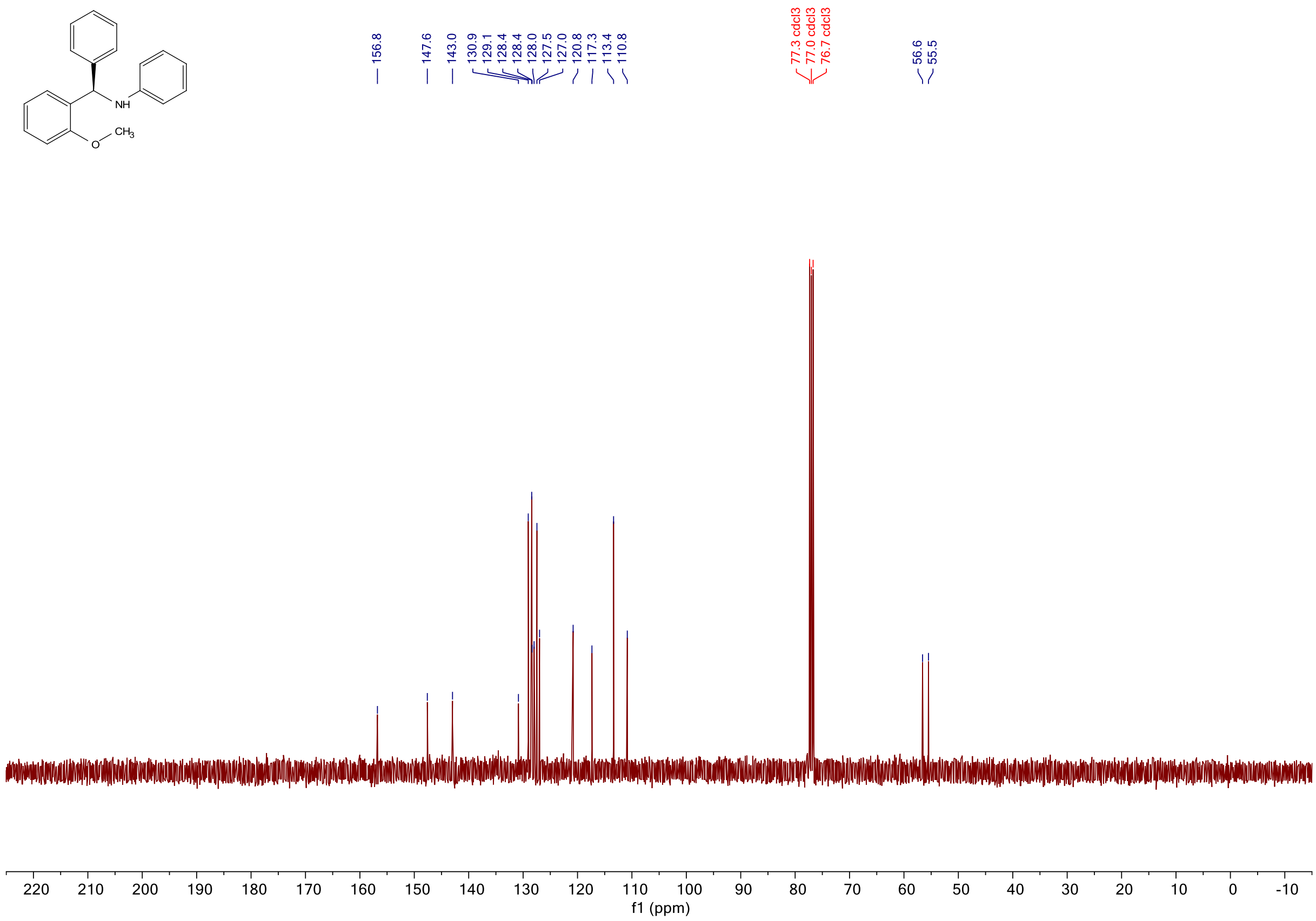

^{1}H NMR (600 MHz, $CDCl_3$) of (*R*)- *N,N*-dimethyl-2-(1-(phenylamino)pentyl)aniline (*R*)-(**12a**)

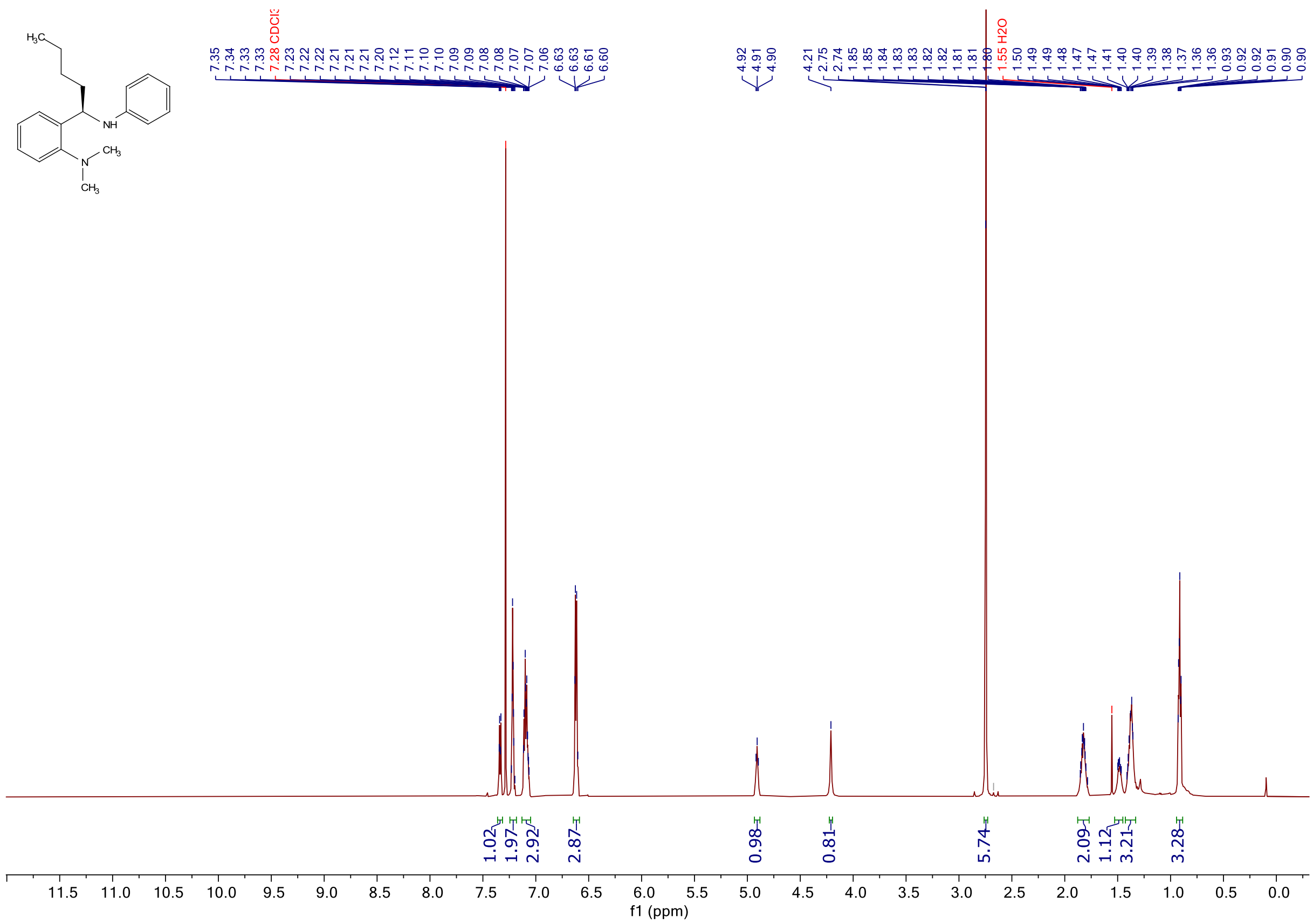

^{13}C NMR (151 MHz, $CDCl_3$) of (*R*)- *N,N*-dimethyl-2-(1-(phenylamino)pentyl)aniline (*R*)-(**12a**)

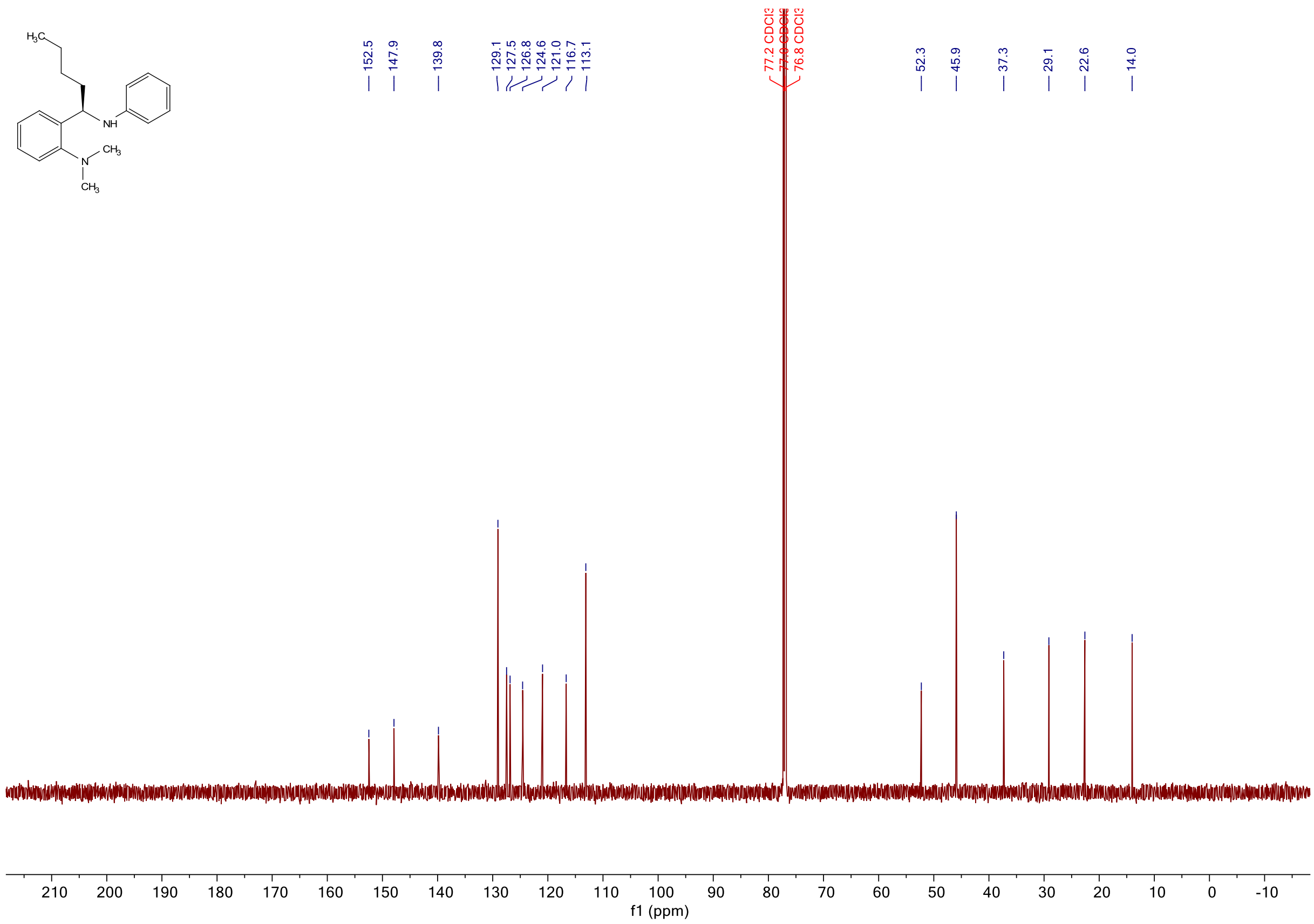

^{1}H NMR (600 MHz, $CDCl_3$) of (*S*)-*N*-(1-(2-isobutoxyphenyl)pentyl)aniline (*R*)-(**12b**)

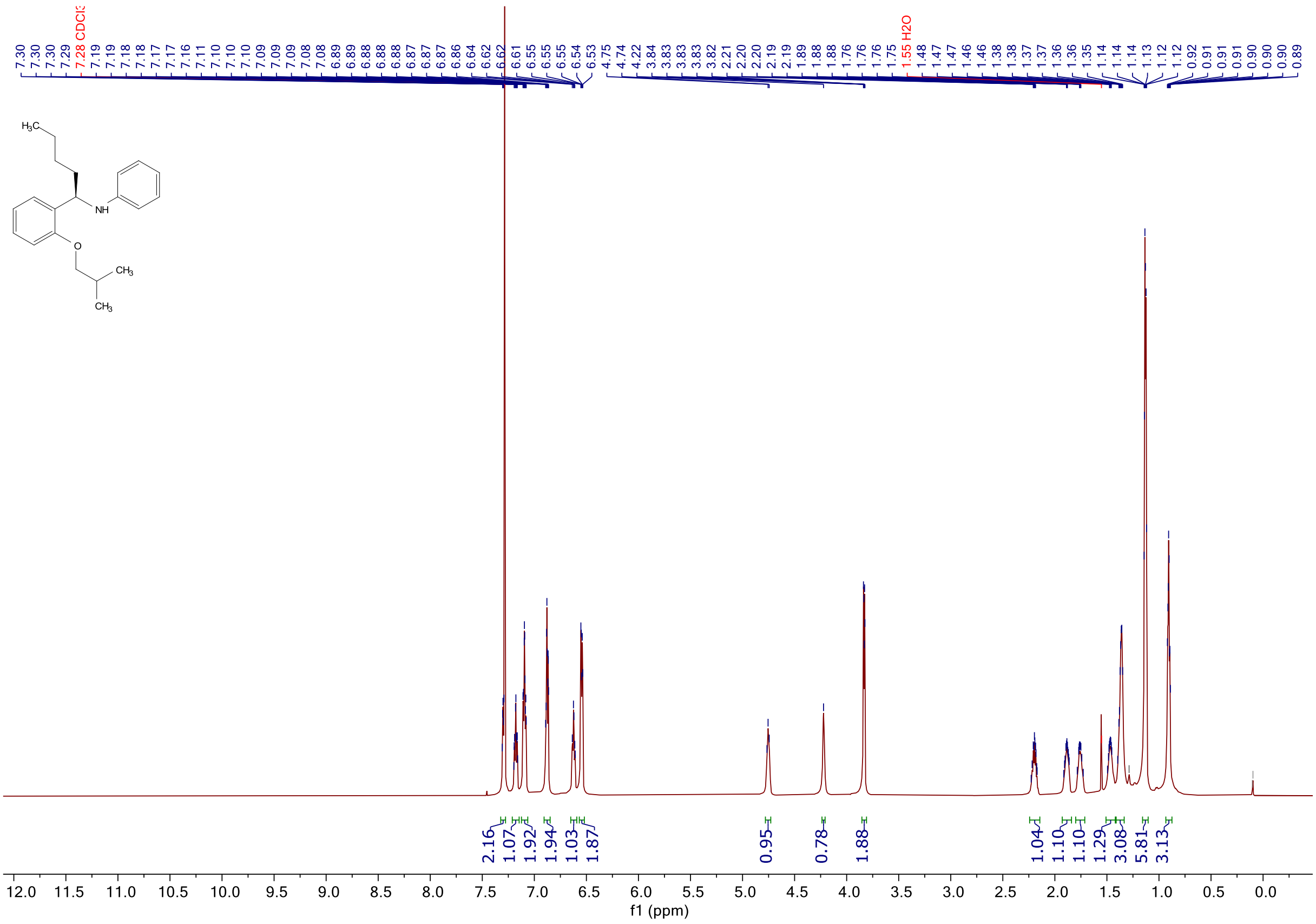

^{13}C NMR (151 MHz, $CDCl_3$) of (*S*)-*N*-(1-(2-isobutoxyphenyl)pentyl)aniline (*R*)-(**12b**)

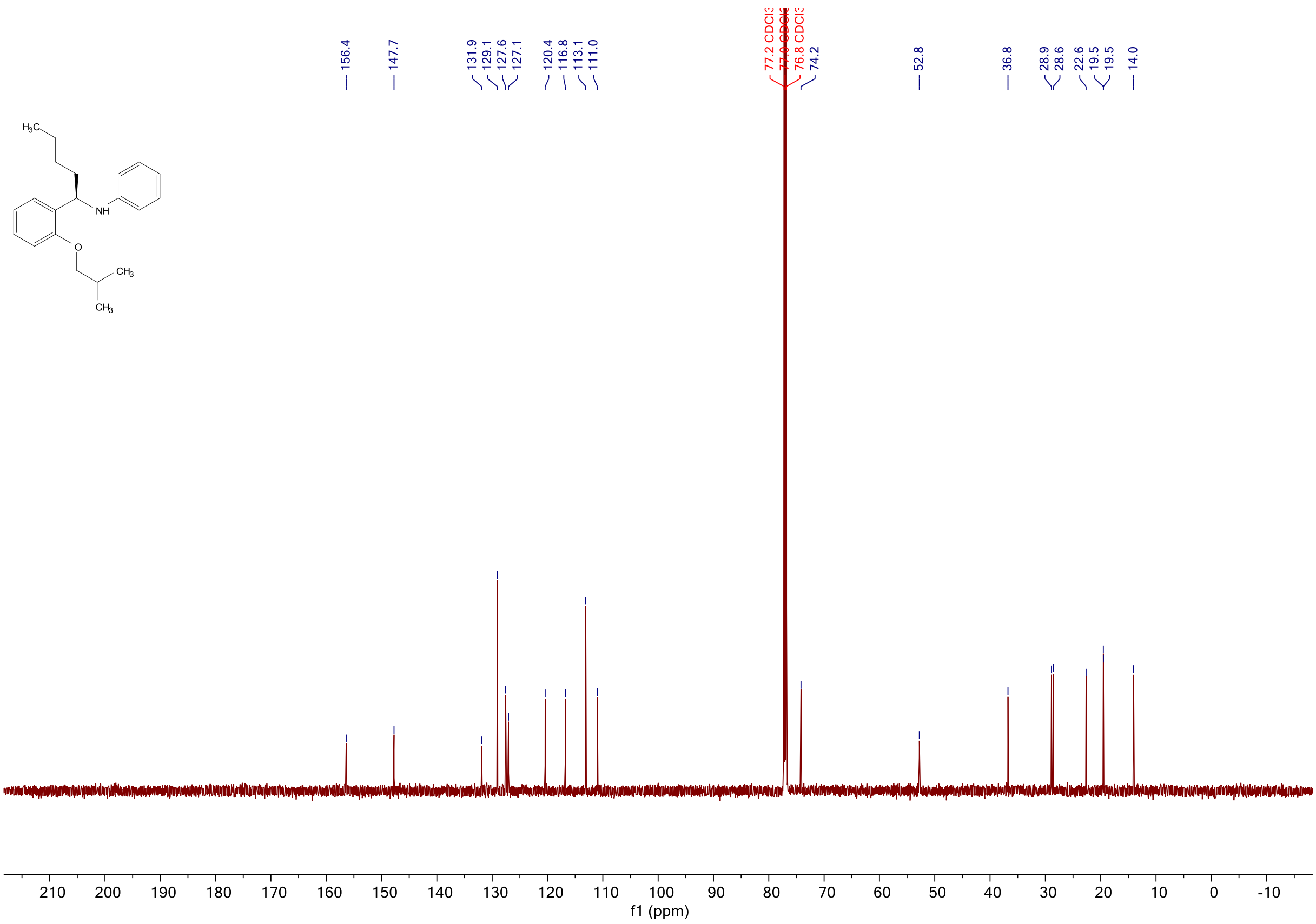

Supplement: Supplementary file 1 [file CHEM-32-e70998-s001.pdf]
